# Connexin43 Deficiency Leads to Ventricular Arrhythmias by Reprogramming Proline Metabolism

**DOI:** 10.1002/advs.202516090

**Published:** 2026-01-31

**Authors:** Hangying Ying, Hangping Fan, Yunhe Wang, Ruhong Jiang, Dongsheng Cai, Hui Cheng, Hao Wang, Chenyang Jiang, Ping Liang

**Affiliations:** ^1^ Department of Cardiology Sir Run Run Shaw Hospital Zhejiang University School of Medicine Hangzhou China; ^2^ Zhejiang Key Laboratory of Cardiovascular Intervention and Precision Medicine Hangzhou China; ^3^ Engineering Research Center for Cardiovascular Innovative Devices of Zhejiang Province Hangzhou China; ^4^ Key Laboratory of Combined Multi‐Organ Transplantation Ministry of Public Health the First Affiliated Hospital Zhejiang University School of Medicine Hangzhou China; ^5^ Institute of Translational Medicine Zhejiang University Hangzhou China; ^6^ Assisted Reproduction Unit Department of Obstetrics and Gynecology Sir Run Run Shaw Hospital Zhejiang University School of Medicine Hangzhou China

**Keywords:** connexin43, iPSC‐CMs, proline, ROS, ventricular arrhythmias

## Abstract

Ventricular arrhythmias (VAs) as life‐threatening heart rhythm disorders, reduced connexin43 (Cx43) is one of the mechanisms of VAs. Cx43 is the predominant ventricular gap junction protein essential for cardiac electrical conduction; the absence in the mouse heart results in sudden arrhythmic death. However, the mechanism linking Cx43 downregulation and VA formation remains unclear. Here it is aimed to elucidate the molecular mechanism by which Cx43 deficiency leads to VAs using Cx43 knockout (Cx43‐KO) induced pluripotent stem‐derived cardiomyocytes and cardiac‐specific conditional Cx43‐KO (Cx43‐cKO) mice. It is shown that Cx43‐KO induced arrhythmic phenotype and decreased proline content both in vitro and in vivo. Mechanistically, Cx43 interacts with the amino acid transporter SNAT2 (sodium‐dependent neutral amino acid transporter). Cx43 deficiency reduces SNAT2 expression, impairing proline transport and metabolism. This disruption leads to mitochondrial dysfunction, oxidative stress, abnormal calcium handling, and arrhythmias. Exogenous proline supplementation rescued the arrhythmic phenotype in Cx43‐cKO mice by restoring metabolic balance. In conclusion, it is suggested that Cx43 deficiency leads to VAs through SNAT2‐mediated proline metabolic reprogramming. Targeting proline metabolism may therefore offer novel therapeutic strategies for VAs.

## Graphical abstract

The study demonstrated that connexin43 (Cx43) knockout caused arrhythmic phenotype and decreased proline content in vitro and in vivo. Mechanistically, Cx43 interacts with the amino acid transporter SNAT2 (sodium‐dependent neutral amino acid transporter), and its deficiency disrupts proline transport and metabolism. This leads to mitochondrial dysfunction, oxidative stress, calcium‐handling defects, and arrhythmias. Targeting proline metabolism may offer novel therapeutic strategies for Cx43 deficiency–associated ventricular arrhythmias.

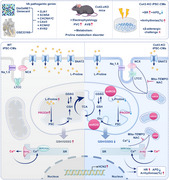

## 1 Introduction

Ventricular arrhythmias (VAs) are abnormal heart rhythms that originate from the ventricles, which are characterized primarily by disturbances in the ventricular electrical activation sequence, frequency, or rhythm. VAs that encompass premature ventricular contraction (PVC), ventricular tachycardia (VT), ventricular flutter (Vf), and ventricular fibrillation (VF) are common cardiac arrhythmias in clinical practice [1]. VAs may occur in patients with normal or structurally diseased hearts, and show a great variability of clinical manifestations [2]. While some patients with VAs may otherwise be asymptomatic, some may present with sudden cardiac death (SCD) [3]. VAs can be the sole or earliest manifestation of preclinical cardiac diseases, and most malignant arrhythmias occur on structurally diseased hearts [4]. Accumulating evidence indicates that preserving gap junctions or regenerating cardiac conduction systems via expression of connexin40 could prevent VAs [5, 6]. Connexin hemichannels serve as candidate targets for the antiarrhythmic treatment [7]. Gap junctional remodeling results in the appearance of re‐entry and potential electrical instability, and there has been a growing interest in how connexins serve as the substrate for VAs.

Connexin43 (Cx43), the predominant ventricular gap junction protein, is critical for maintaining normal cardiac electrical conduction. Reduced Cx43 expression is one of the mechanisms of VAs, and the absence of Cx43 in the mouse heart results in sudden arrhythmic death [8]. Previous studies on Cx43 deficiency–associated VAs have primarily focused on alterations in the distribution and function of Cx43, which lead to re‐entry and the onset of arrhythmias [9, 10]. Despite these specific findings, the molecular mechanisms underlying Cx43 deficiency have not been fully clarified. However, in a canine myocardial infarction model, lateralization and degradation of Cx43 in epicardial border zone cardiomyocytes have been reported to promote arrhythmia by facilitating reentrant circuits [9, 11]. Similarly, in a neonatal rat ventricular myocyte model, adenovirus‐mediated inhibition of Cx43 was shown to result in regional heterogeneity of Cx43 suppression, then promoting conduction delay and wave break, and ultimately leading to the formation of re‐entrant circuits [10]. Moderately severe reductions in Cx43 abundance are also associated with a slowing of impulse propagation and a dramatic increase in the susceptibility to inducible ventricular arrhythmias [12], and where electrical remodeling contributes to complex tachyarrhythmias in Cx43‐deficient mouse hearts [13]. One previous study has also recognized Cx43 as a scaffolding protein that modulates the functional attributes of its interacting proteins [14]. Our aim here is to provide clarification of the mechanistic link between Cx43 deficiency and VA formation.

Proline, a nonessential amino acid, serves not only as a fundamental component for protein synthesis but also participates in energy metabolism and the regulation of redox balance [15, 16]. Proline has recently received close attention for its importance in cardiac metabolism. Studies have demonstrated that proline ameliorates myocardial ischemic injury through its antioxidant capacity [17], and overexpression of proline dehydrogenase (PRODH) in cardiomyocytes reprograms proline metabolism to replenish tricarboxylic acid cycle intermediates and restore glutathione redox balance [16]. In this regard, proline catabolism is considered critical to the maintenance of normal mitochondrial function and ATP production during hypoxia in cardiomyocytes [18]. However, the role of proline metabolism in the arrhythmogenesis and its regulatory mechanism remain thus far unknown.

Through deep mining of the public databases, we identify the *GJA1* gene, encoding the predominant ventricular gap junction protein Cx43, as a crucial gene for VAs. Utilizing induced pluripotent stem cell–derived cardiomyocytes (iPSC‐CMs) and genetic mice as model systems, we demonstrated that Cx43 deficiency leads to arrhythmic phenotypes, along with reduced levels of proline. Mechanistically, Cx43 interacts with the amino acid transporter SNAT2 (sodium‐dependent neutral amino acid transporter, encoded by the *SLC38A2* gene) where Cx43 deficiency downregulates SNAT2 expression causing reduced proline transport into cardiomyocytes and resulting in proline metabolic dysfunction. This ultimately leads to disrupted mitochondrial dynamics and oxidative stress. On the one hand, disrupted proline metabolism fails to provide additional energy supply for the accelerated heart rate and impairs oxidative stress tolerance, leading to impaired mitochondrial dynamics and function. On the other hand, excessive oxidative stress causes calcium homeostasis imbalance, ultimately leading to arrhythmogenesis. Our findings elucidate the molecular mechanism of VA formation caused by Cx43 deficiency from the perspective of proline metabolic reprogramming independent of re‐entry mechanisms. We suggest that targeting proline metabolism could aid in the development of new effective treatment strategies for Cx43 deficiency–associated VAs.

## 2 Results

### 2.1 *GJA1* Is Identified as One of the Crucial Genes for VAs

**FIGURE 1:**
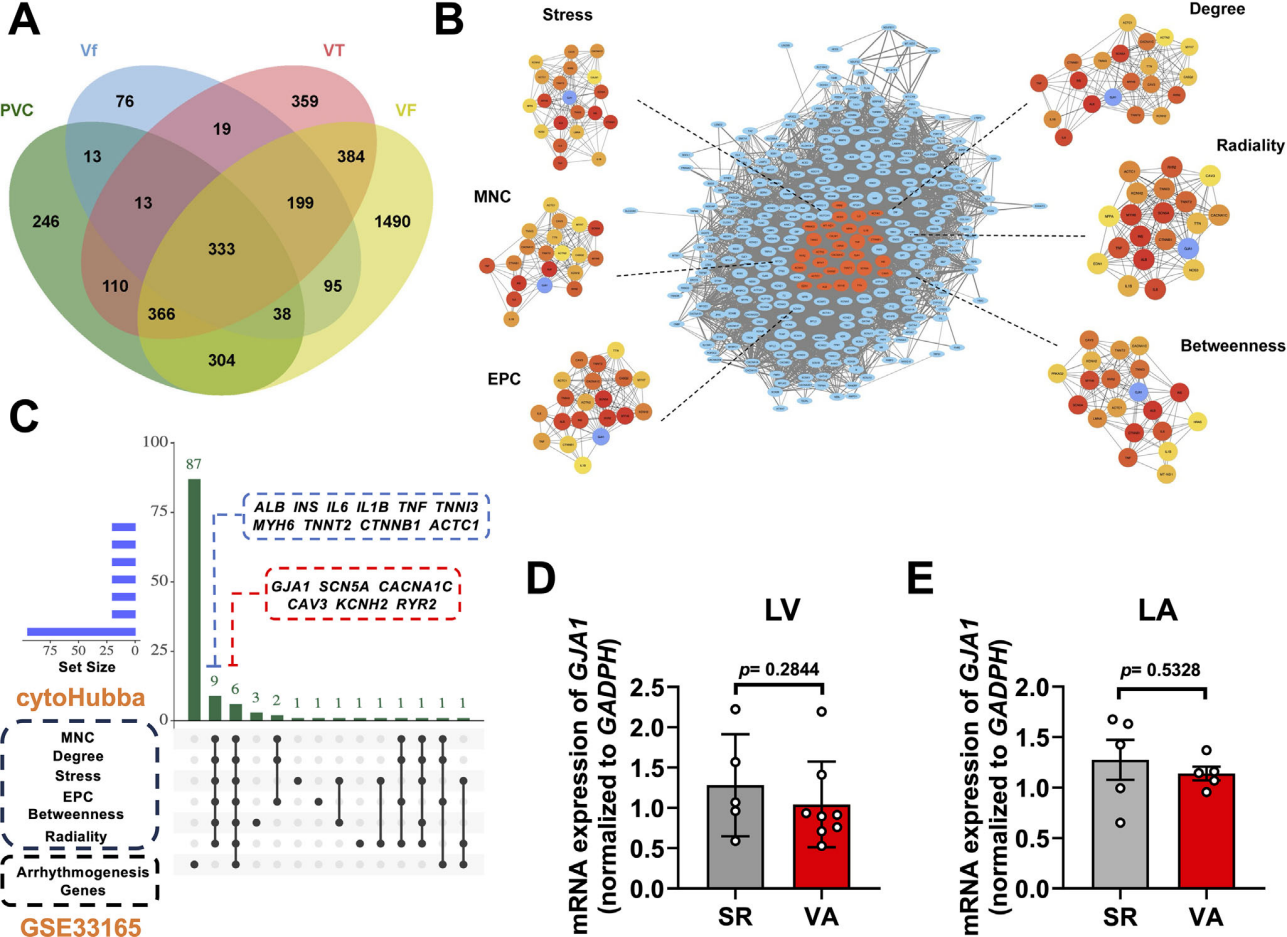
*GJA1* is identified as one of the crucial genes for ventricular arrhythmias. (A) Venn diagram of the ventricular arrhythmia (VA) putative genes (PVC: premature ventricular contraction; VT: ventricular tachycardia; Vf: ventricular flutter; and VF: ventricular fibrillation) obtained from the OMIM and DisGeNET databases. (B) Visualization of the protein–protein interaction (PPI) network of the obtained genes from Panel A, and top 20 hub genes identified by six algorithms including Stress, MNC, EPC, Degree, Radiality, and Betweenness. The list of genes included in the algorithm is provided in Table S1. (C) Upset plot displaying six VA genes identified by external data (GSE33165) and hub genes. Details of the external patient cohort are provided in Table S2. (D) Bar graph to compare the mRNA expression of *GJA1* in left ventricle (LV) between patients with sinus rhythm (SR, *n* = 5) and VA (*n* = 8). We averaged the epicardial and endocardial expression values from three nonischemic hearts in the VA group, and applied averaged values as the expression values for the hearts. (E) Bar graph to compare the mRNA expression of *GJA1* in left atrium (LA) between patients with SR (*n* = 5) and VA (*n* = 5). *p* values were calculated using (D) Mann–Whitney test and (E) unpaired two‐tailed Student's *t*‐test. Data were shown as mean ± SEM.

To assess the critical genes for VAs, we obtained the listed VAs (PVC, VT, Vf, and VF)‐related genes available in the DisGeNET and GeneCards databases. By taking the intersection, we jointly identified a total of 333 genes involved in VAs (Figure 1A), and then constructed the related protein–protein interaction (PPI) network using crossed genes (Figure 1B). The network consisted of 321 nodes and 5113 edges (Figure 1B). Six algorithms of CytoHubba, including Stress, EPC, Degree, Betweenness, Radiality, and MNC, were used to identify the top 20 hub genes in each algorithm (Figure 1B and Table S1). Fifteen genes were identified using the intersection of six algorithms, which were further narrowed down to six genes using the external data in the following sections [19] (Figure 1C). The external data (GSE33165) included messenger RNA (mRNA) expression levels of genes that encode ion channels, calcium‐handling proteins, and transcription factors, which are implicated in the arrhythmogenesis in the left atria (LA) and left ventricle (LV) of the failing human heart (Table S2). Compared to the hearts of the patients with sinus rhythm (SR), the hearts of VA patients exhibited a trend toward a decrease in *GJA1* mRNA levels albeit not statistically different (Figure 1D,E). These results suggest that the expression level of *GJA1* is downregulated in VA hearts.

### 2.2 Cx43 Knockout Causes Arrhythmias and Abnormal Action Potential Properties in iPSC‐CMs

**FIGURE 2:**
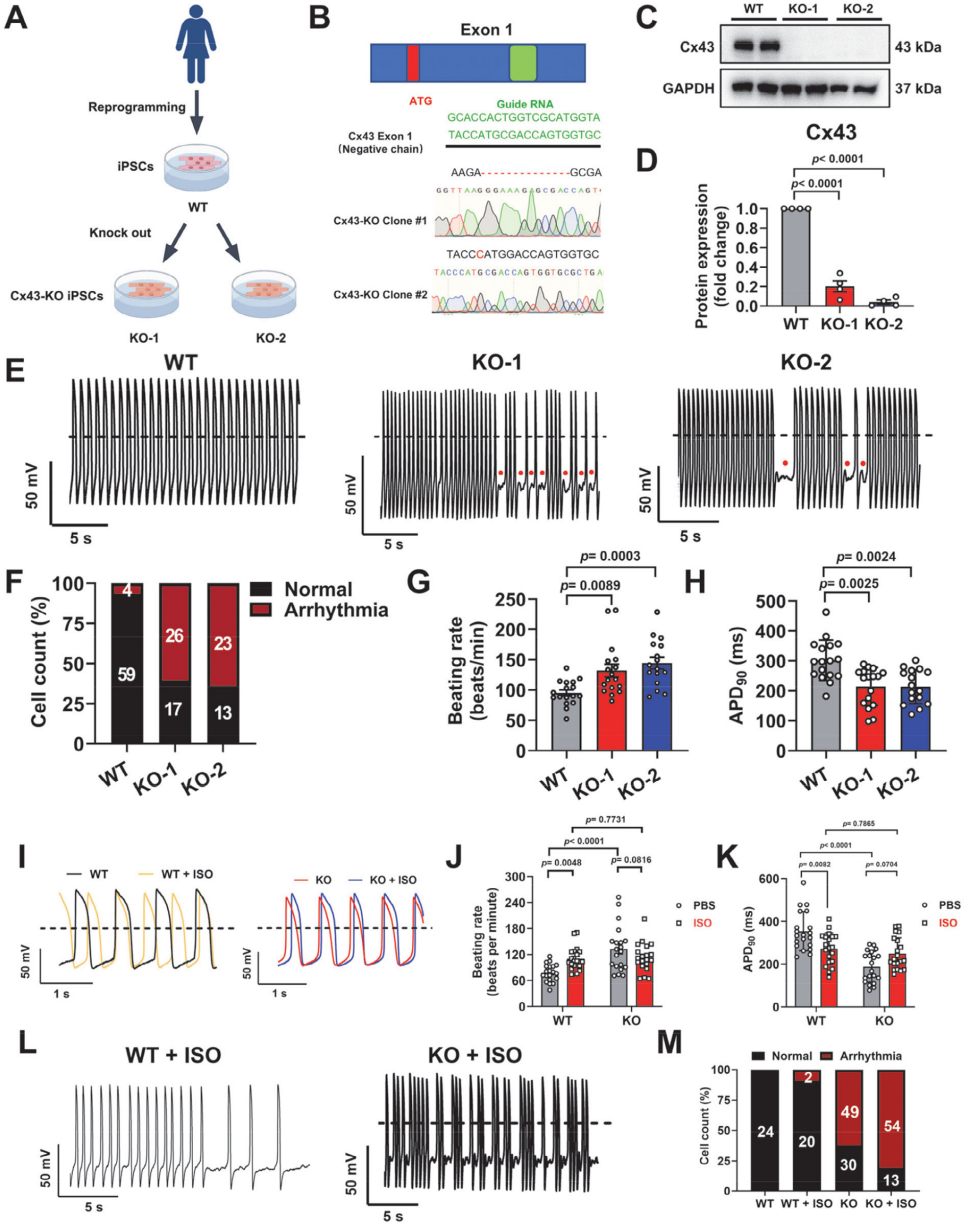
Connexin43 knockout causes arrhythmias and abnormal action potential properties in induced pluripotent stem cell–derived cardiomyocytes. (A) Schematic diagram of wild‐type (WT) and connexin43 knockout (Cx43‐KO) induced pluripotent stem cells (iPSCs) generated in this study. KO‐1 and KO‐2 denote two different Cx43‐KO iPSC clones. (B) The structure of the *GJA1* gene and the location of the guide RNA (gRNA) used for genome editing by CRISPR/Cas9. Sanger sequencing revealed a 14 base‐pair deletion in KO‐1 iPSCs, and a 1 base‐pair insertion in KO‐2 iPSCs, respectively. (C,D) Western blot analysis of Cx43 protein expression in WT and Cx43‐KO iPSC‐derived cardiomyocytes (iPSC‐CMs). *n* = 4 independently biological repeats. Glyceraldehyde‐3‐Phosphate Dehydrogenase is used as the loading control. (E) Representative action potential waveforms recorded by single‐cell patch clamp from WT and Cx43‐KO iPSC‐CMs. Dash lines indicate 0 mV. Red dots indicate arrhythmias. (F) Bar graph to compare the percentage of cells with arrhythmia between WT and Cx43‐KO iPSC‐CMs. (G,H) Bar graphs to compare the beating rate and action potential duration at 90% of repolarization (APD_90_) between WT and Cx43‐KO iPSC‐CMs. *n* = 16–17 cells. (I) Representative action potential waveforms recorded by single‐cell patch clamp from WT and Cx43‐KO iPSC‐CMs with or without isoproterenol (ISO, 100 nm, 2 h) stimulation. Dash lines indicate 0 mV. (J,K) Bar graphs to compare the beating rate and APD_90_ in WT and Cx43‐KO iPSC‐CMs with or without ISO stimulation. *n* = 20 cells. (L) Representative abnormal action potential waveforms recorded by single‐cell patch clamp from WT and Cx43‐KO iPSC‐CMs upon ISO stimulation. Dash lines indicate 0 mV. (M) Bar graph to compare the percentage of cells with arrhythmia in WT and Cx43‐KO iPSC‐CMs with or without ISO stimulation. *n* = 22–79 cells. “KO” in the figure panels refers to combined data from KO‐1 and KO‐2 analyzed in parallel in Panels (I–M). *p* values were calculated using one‐way Analysis of Variance followed by (D) Dunnett's multiple comparisons test, Brown–Forsythe ANOVA test/Welch ANOVA test followed by (G) Dunnett T3 multiple comparisons test, (H) Kruskal–Wallis test followed by Dunn's multiple comparison test, or (J) two‐way ANOVA followed by Uncorrected Fisher's LSD and (K) two‐way ANOVA followed by Tukey's multiple comparisons test. Data were shown as mean ± SEM.

To investigate the mechanisms of Cx43 in regulating VAs in human‐based cardiomyocytes, we generated the Cx43 knockout (Cx43‐KO) iPSC lines (Figure 2A). We designed a single‐guide RNA (sg‐RNA) targeting exon 1 of the *GJA1* gene locus using the CRISPR/Cas9 genome editing system and obtained two different Cx43‐KO iPSC clones (KO‐1 and KO‐2) (Figure 2B). Western blot analysis revealed significantly downregulated expression levels of Cx43 in two different KO iPSCs (Figure 2C,D). Moreover, Cx43‐KO iPSCs exhibited characteristic pluripotent stem cell morphology displaying alkaline phosphatase (ALP) activity (Figure S1A), pluripotency marker expression (Figure S1B–D), the maintenance of normal karyotypes (Figure S1E), and could differentiate into three germ layers (Figure S1F). No off‐target occurrences were observed in Cx43‐KO iPSCs (Figure S2). Wild‐type (WT) and Cx43‐KO iPSCs were subsequently differentiated into cardiomyocytes using a two‐dimensional (2D) small molecule‐based cardiomyocyte differentiation protocol (Figure S3A). Immunofluorescent staining and fluorescence‐activated cell sorting (FACS) analysis demonstrated that Cx43‐KO had not influenced cardiomyocyte differentiation ability or efficiency (Figure S3B–E).

Next, we assessed the electrophysiological properties at the single‐cell level using patch clamp. Action potentials were recorded from WT and Cx43‐KO ventricular‐like iPSC‐CMs, which revealed a greatly higher percentage of cells with arrhythmia (Figure 2E,F). Moreover, Cx43‐KO iPSC‐CMs exhibited significantly faster beating rate, hyperpolarized maximal diastolic potential (MDP), decreased overshoot, and shortened APD_50_ (action potential duration at 50% of repolarization) and APD_90_ (action potential duration at 90% of repolarization) in Cx43‐KO iPSC‐CMs, whereas action potential amplitude (APA) and maximal velocity rate (*V*_max_) were comparable between WT and Cx43‐KO iPSC‐CMs (Figure 2G,H and Table S3). Meanwhile, field potentials were recorded from iPSC‐CMs using multi‐electrode array (MEA) (Figure S4A). Consistent with the patch clamp data, we found significantly faster beating rate, and shortened field potential duration (FPD) and corrected FPD (FPDc) in Cx43‐KO iPSC‐CMs (Figure S4B–E). Notably, Cx43‐KO iPSC‐CMs also displayed a markedly slower conduction velocity (Figure S4F). The electrophysiological properties were comparable between myocytes from two different Cx43‐KO iPSC clones (Figure 2A–H and Figure S4). Taken together, these results demonstrate that Cx43‐KO causes arrhythmias and abnormal action potential properties in iPSC‐CMs.

### 2.3 β‐Adrenergic Stimulation Exacerbates the Arrhythmic Phenotype in Cx43‐KO iPSC‐CMs

Hyperactivation of cardiac β‐adrenergic signaling results in oxidative stress, hypertrophy, and fibrosis, and predisposes the heart to be more susceptible to malignant arrhythmias [20, 21]. In the clinic, short‐term isoproterenol (ISO) stimulation is utilized to evoke arrhythmias and elevate heart rate [21]. Immunofluorescence analysis of Cx43 revealed that ISO stimulation promoted the gap junction formation in WT iPSC‐CMs, but not in Cx43‐KO iPSC‐CMs (Figure S5A). It has been shown that gap junctions formed between cardiomyocytes via microtubule‐dependent transport can reduce the occurrence of arrhythmia [22]. We conducted α‐ and β‐tubulin staining in WT and Cx43‐KO iPSC‐CMs with or without ISO simulation, and found that neither Cx43‐KO nor ISO stimulation impaired the microtubule system of iPSC‐CMs (Figure S5B). Additionally, ISO stimulation failed to cause cell death in either WT or Cx43‐KO iPSC‐CMs (Figure S5C,D). Therefore, we investigated the electrophysiological changes in WT and Cx43‐KO iPSC‐CMs upon ISO stimulation (100 nm, 2 h). As expected, ISO stimulation caused markedly faster beating rate and shortened APD_90_ in WT iPSC‐CMs, with arrhythmias evoked in only 2 out of 22 cells (Figure 2I–K,M). This was not the case in Cx43‐KO iPSC‐CMs, where the beating rate was already preaccelerated. In this case, we found that Cx43‐KO iPSC‐CMs displayed a greatly higher proportion of cells with arrhythmia in response to ISO stimulation (54 out of 67 cells), with beating rate and APD_90_ remaining unchanged (Figure 2I–M). MEA recordings also revealed significantly less changes in beating rate, FPD, and conduction velocity in Cx43‐KO iPSC‐CMs upon ISO stimulation, as compared to WT iPSC‐CMs (Figure S5E–H). These results indicate that β‐adrenergic stimulation exacerbates the arrhythmic phenotype in Cx43‐KO iPSC‐CMs.

### 2.4 Cx43‐KO Leads to Metabolic Reprogramming in iPSC‐CMs

**FIGURE 3:**
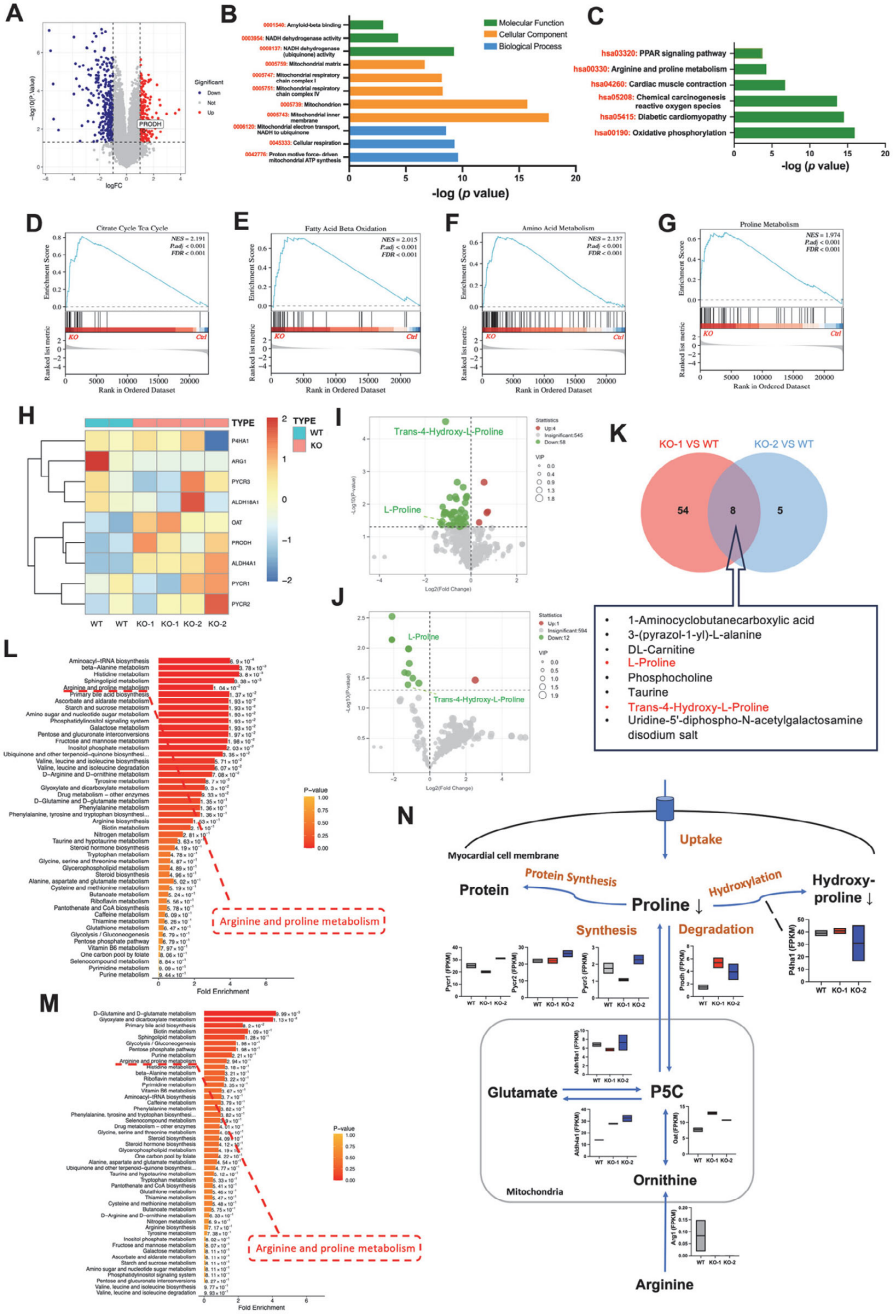
Cx43‐KO leads to metabolic reprogramming in iPSC‐CMs. (A) Volcano plot of differentially expressed genes (DEGs) between WT and Cx43‐KO iPSC‐CMs. (B) Gene ontology (GO) enrichment analysis of upregulated DEGs between WT and Cx43‐KO iPSC‐CMs. (C) Kyoto encyclopedia of genes and genomes (KEGG) enrichment analysis of upregulated DEGs between WT and Cx43‐KO iPSC‐CMs. (D–G) Gene set enrichment analysis (GSEA) demonstrating positively significant enrichment of metabolic‐related pathways including “tricarboxylic acid (TCA) cycle,” “fatty acid β oxidation,” “amino acid metabolism,” and “proline metabolism” in Cx43‐KO iPSC‐CMs. (H) Heatmap demonstrating the gene expression of proline metabolism between WT and Cx43‐KO iPSC‐CMs. (I,J) Volcano plots of differentially expressing metabolites (DEMs) between WT and KO‐1 iPSC‐CMs, as well as between WT and KO‐2 iPSC‐CMs. (K) Venn diagram of DEMs between WT and Cx43‐KO iPSC‐CMs. The DEMs are displayed in a box, and those involved in proline metabolism are highlighted in red. (L,M) Metabolite set enrichment analysis (MSEA) of DEMs between WT and KO‐1 iPSC‐CMs, as well as between WT and KO‐2 iPSC‐CMs. (N) Differential expression of key genes in arginine and proline metabolism pathway between WT and Cx43‐KO iPSC‐CMs. KO‐1 and KO‐2 represent two different Cx43‐KO iPSC clones. *n* = 2 independently biological replicates for WT, KO‐1, and KO‐2.

An elevated heart rate requires a large number of substrates and an adequate energy supply [23], we hypothesized that metabolic processes had been remodeled in Cx43‐KO iPSC‐CMs. To test this hypothesis, we first performed RNA sequencing (RNA‐Seq) using RNA samples from WT and Cx43‐KO iPSC‐CMs. Box plots showed no difference in the values and distribution of FPKM (fragments per kilobase of transcript per million mapped reads) among the groups (Figure S6A,B). Principal component analysis (PCA) and correlation analysis showed good reproducibility within the groups and significant differences between the groups (Figure S6C–E), indicating that the RNA‐Seq data were of good quality. We found a total of 852 differentially expressed genes (DEGs) in common in myocytes derived from two different Cx43‐KO iPSC clones (KO‐1 and KO‐2). The transcript of the *PRODH* gene, encoding the first enzyme of proline catabolism, was upregulated in Cx43‐KO iPSC‐CMs (Figure 3A). Gene ontology (GO) analysis revealed that genes were positively enriched in “Nicotinamide adenine dinucleotide dehydrogenase activity,” “mitochondrial respiratory chain complexes I and IV,” and “proton motive force‐driven mitochondrial ATP synthesis” in Cx43‐KO iPSC‐CMs (Figure 3B and Figure S6F,G). In addition, analysis using the Kyoto encyclopedia of genes and genomes (KEGG) revealed that “arginine and proline metabolism,” “chemical carcinogenesis reactive oxygen species,” and “oxidative phosphorylation (OXPHOS)” pathways were significantly enriched in the upregulated genes in Cx43‐KO iPSC‐CMs (Figure 3C and Figure S6H,I). Similarly, gene set enrichment analysis (GSEA) demonstrated positively significant enrichment of metabolic‐related pathways including “tricarboxylic acid (TCA) cycle,” “fatty acid β oxidation,” “amino acid metabolism,” and “proline metabolism” in Cx43‐KO iPSC‐CMs (Figure 3D–G). Genes involved in arginine and proline metabolism, *PRODH* and *ALDH4A1* (which encode a mitochondrial NAD‐dependent dehydrogenase that converts P5C to glutamate in proline catabolism), were clustered together and upregulated in Cx43‐KO iPSC‐CMs (Figure 3H).

After elucidating the metabolic alterations through RNA‐Seq in Cx43‐KO iPSC‐CMs, we further performed widely targeted metabolomics to determine metabolic substrates. Orthogonal partial least‐squares–discriminant analysis (OPLS‐DA) revealed distinct metabolite profile in each sample (Figure S7A–D). Hierarchical clustering trees indicated both similarities and differences between groups (Figure S7E,F). The volcano plots show the difference in metabolites between WT and Cx43‐KO iPSC‐CMs. Most of the differential metabolites were downregulated in Cx43‐KO iPSC‐CMs (Figure 3I,J). A total of 8 common metabolites were obtained by intersection of the two different comparisons (KO‐1 vs. WT and KO‐2 vs. WT), of which “l‐proline” and “*trans*‐4‐hydroxy‐l‐proline”, both involved in proline metabolism, were downregulated in Cx43‐KO iPSC‐CMs (Figure 3K and Figure S7G,H). Strikingly, the content of glutamate, the substrate for proline synthesis, did not significantly change between WT and Cx43‐KO iPSC‐CMs (Figure 3K and Figure S7G,H). Moreover, we conducted metabolite set enrichment analysis (MSEA) for top 50 pathways ranked based on *p* values, which revealed that “arginine and proline metabolism” was significantly enriched in Cx43‐KO iPSC‐CMs (Figure 3L,M). Similarly, KEGG analysis also found that “arginine and proline metabolism” was enriched in Cx43‐KO iPSC‐CMs (Figure S7I,J). The upregulation of the *PRODH* transcript suggested that Cx43‐KO may lead to abnormal proline catabolism in cardiomyocytes (Figure 3N). Altogether, these results demonstrate that Cx43‐KO leads to metabolic reprogramming in iPSC‐CMs.

### 2.5 Cx43‐KO iPSC‐CMs Exhibit Disrupted Mitochondrial Dynamics and Oxidative Stress

**FIGURE 4:**
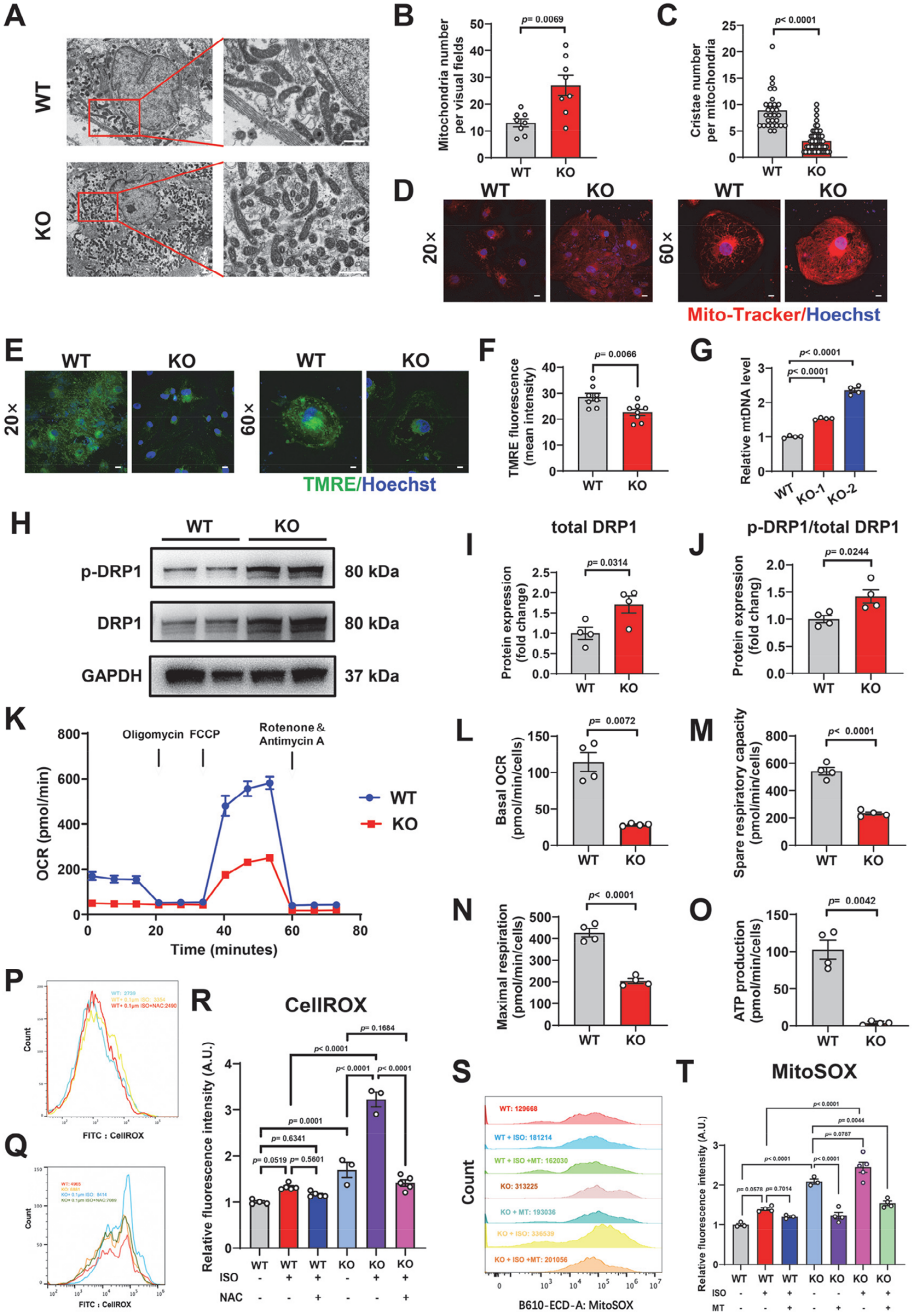
Cx43‐KO iPSC‐CMs exhibit disrupted mitochondrial dynamics and oxidative stress. (A) Representative graphs of mitochondrial ultrastructure in WT and Cx43‐KO iPSC‐CMs using transmission electron microscopy (TEM). Scale bar, 5 µm. (B,C) Bar graphs to compare the mitochondrial number and mitochondrial cristae number between WT and Cx43‐KO iPSC‐CMs. *n* = 8 random fields. (D) Representative graphs of mitochondrial morphology labeled with Mito‐Tracker in WT and Cx43‐KO iPSC‐CMs. Scale bar, 100 µm. (E) Representative graphs of mitochondrial membrane potential labeled with a fluorescent dye (tetramethylrhodamine ethyl ester, TMRE) in WT and Cx43‐KO iPSC‐CMs. Scale bar, 100 µm. (F) Bar graph to compare the TMRE fluorescence intensity between WT and Cx43‐KO iPSC‐CMs. *n* = 8 independently biological repeats. (G) Bar graph to compare the mitochondrial DNA (mtDNA) content between WT and Cx43‐KO iPSC‐CMs. *n* = 4 independently biological repeats. (H–J) Western blot analysis of the protein expression of total dynamin‐related protein 1 (DRP1) and phosphorylated DRP1 (DRP1‐Ser616) in WT and Cx43‐KO iPSC‐CMs. GAPDH is used as the loading control. *n* = 4 independently biological repeats. (K) The diagram depicting the traces of oxygen consumption rate (OCR) on WT and Cx43‐KO iPSC‐CMs after sequentially administration of 1.5 µm oligomycin (ATP synthase inhibitor), 4 µm FCCP (uncoupler of oxidative phosphorylation in mitochondria) and 1 µm antimycin A (electron transport chain blocker), respectively. (L–O) Bar graphs to compare a series of fundamental parameters of mitochondrial function between WT and Cx43‐KO iPSC‐CMs, including basal OCR, spare respiratory capacity, maximal respiration and ATP production. *n* = 4 independently biological repeats. (P–R) Measurement of cellular reactive oxygen species (ROS) levels in WT and Cx43‐KO iPSC‐CMs with or without ISO (100 nm, 2 h) stimulation and/or *N*‐acetyl‐l‐cysteine (NAC, 5 mm, 4 hours) treatment. *n* = 3–5 independently biological repeats. (S,T) Measurement of mitochondrial ROS levels in WT and Cx43‐KO iPSC‐CMs with or without ISO (100 nm, 2 h) stimulation and/or Mito‐TEMPO (MT, 5 µm, 72 h) treatment. *n* = 3–5 independently biological repeats. “KO” in the figure panels refers to combined data from (A–F,H–T) KO‐1 and KO‐2 analyzed in parallel. (B,L,O) *p* values were calculated using unpaired t test with Welch's correction, (C) Mann–Whitney test, (F,I,J,M,N) unpaired two‐tailed Student's *t*‐test, (G) Brown–Forsythe ANOVA test/Welch ANOVA test followed by Dunnett T3 multiple comparisons test, and (R,T) one‐way ANOVA followed by Tukey's multiple comparisons test. Data were shown as mean ± SEM.

Given that DEGs were positively enriched in mitochondria‐related terms, including “mitochondrial respiratory chain complexes I and IV,” “proton motive force‐driven mitochondrial ATP synthesis,” and “OXPHOS” (Figure 3B,C), we next determined mitochondrial characteristics and oxidative stress levels in Cx43‐KO iPSC‐CMs. Transmission electron microscopy (TEM) analysis showed that Cx43‐KO significantly increased the mitochondrial number and reduced the mitochondrial cristae number (Figure 4A–C). Such mitochondrial morphology phenotypes were verified using Mito‐Tracker staining (Figure 4D). To further explore the mitochondrial function, we conducted mitochondrial membrane potential (MMP) detection using a fluorescent dye (tetramethylrhodamine ethyl ester, TMRE). We observed that the MMP was significantly reduced in Cx43‐KO iPSC‐CMs compared to WT iPSC‐CMs (Figure 4E,F). Moreover, real‐time quantitative polymerase chain reaction (qPCR) analysis revealed a marked increase in the mitochondrial DNA (mtDNA) content in Cx43‐KO iPSC‐CMs (Figure 4G). These results indicate that Cx43‐KO causes impaired mitochondrial dynamics in iPSC‐CMs. Dynamin‐related protein 1 (DRP1) is a critical regulator of mitochondrial fission–fusion dynamics [24]. We found significantly higher levels of total and phosphorylated DRP1 in Cx43‐KO iPSC‐CMs (Figure 4H–J), thereby shifting the balance toward mitochondrial fission. Next, we assessed mitochondrial respiratory function using a Seahorse bioscience XF‐96 extracellular flux analyzer. We observed significantly reduced basal oxygen consumption rates (OCRs), spare respiratory capacity, maximal respiration, and ATP production in Cx43‐KO iPSC‐CMs (Figure 4K–O), indicating an impaired OXPHOS due to Cx43‐KO.

Mitochondrial dysfunction is closely related to excessive reactive oxygen species (ROS) production, with mitochondria being considered to be the predominant cellular source of ROS [25]. We therefore quantitatively assessed the cellular and mitochondrial ROS contents using the fluorescent probes CellROX and MitoSOX combined with flow cytometry. At baseline, both the cellular and mitochondrial ROS levels were significantly elevated in Cx43‐KO iPSC‐CMs, when compared to WT iPSC‐CMs (Figure 4P–T). Following ISO stimulation, the differences of ROS levels between WT and Cx43‐KO iPSC‐CMs became further increased (Figure 4P–T). Notably, treatment of *N*‐acetyl‐l‐cysteine (NAC, an intracellular ROS scavenger) or Mito‐TEMPO (MT, a mitochondrial superoxide scavenger) in Cx43‐KO iPSC‐CMs significantly reversed the cellular or mitochondrial ROS level, respectively, regardless of ISO stimulation (Figure 4P–T). Collectively, these results indicate that Cx43‐KO causes disrupted mitochondrial dynamics and oxidative stress, which are further aggravated under β‐adrenergic stimulation.

### 2.6 ROS‐Mediated Abnormal Calcium Handling Confers the Arrhythmic Phenotype of Cx43‐KO iPSC‐CMs

**FIGURE 5:**
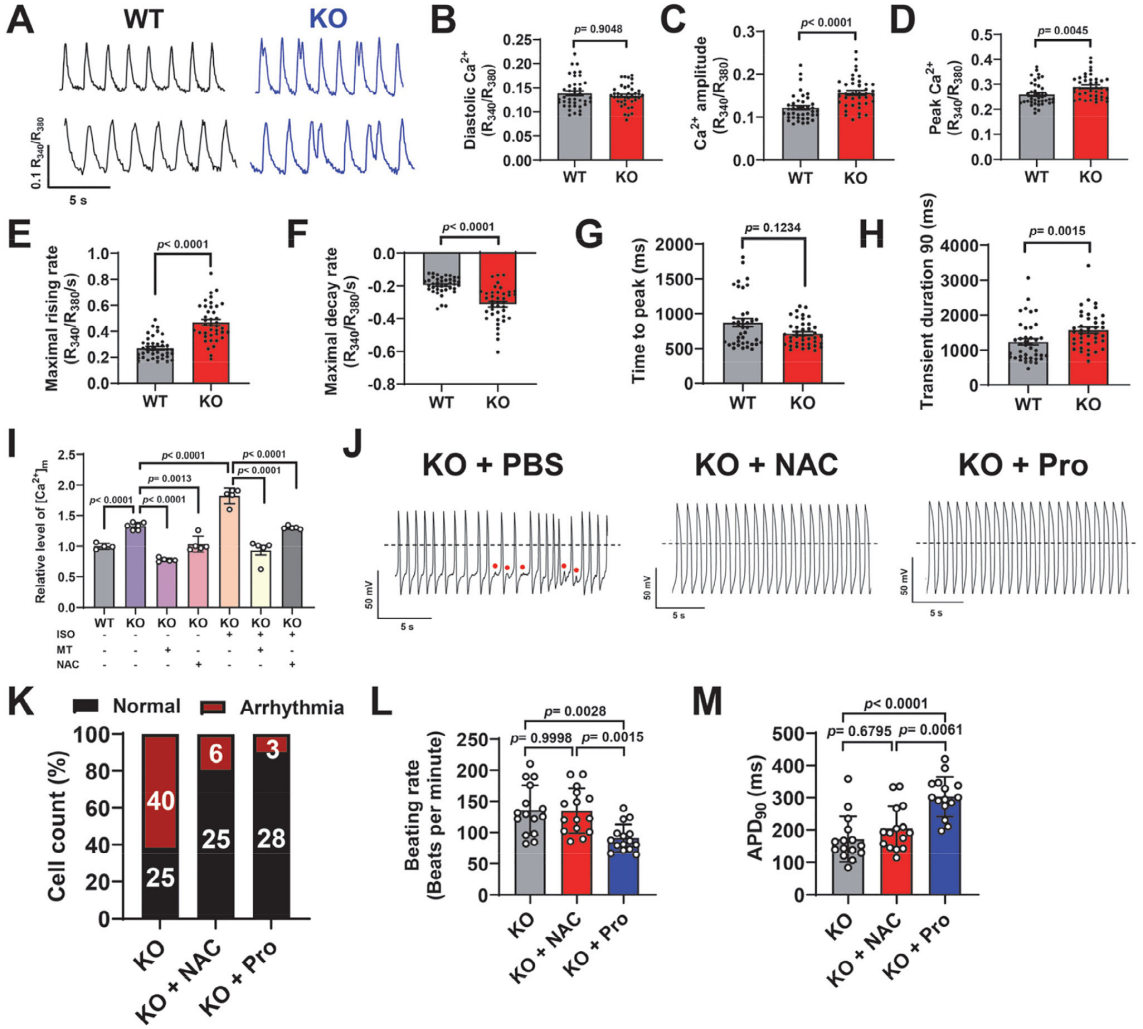
ROS‐mediated abnormal calcium handling confers the arrhythmic phenotype of Cx43‐KO iPSC‐CMs. (A) Representative calcium (Ca^2+^) transient waveforms recorded by ratiometric Fura‐2 imaging from WT and Cx43‐KO iPSC‐CMs. (B–H) Bar graphs to compare key parameters of calcium transients between WT and KO iPSC‐CMs, including diastolic calcium, calcium amplitude, peak calcium, maximal rising rate, maximal decay rate, time to peak, and transient duration 90. *n* = 40 cells. (I) Mitochondrial calcium analysis through Rhod‐2 staining in WT and Cx43‐KO iPSC‐CMs with or without ISO (100 nm, 2 h) stimulation, and with or without treatment of either Mito‐TEMPO (MT, 5 µm, 72 h) or *N*‐acetyl‐l‐cysteine (NAC, 5 mm, 4 h). *n* = 4–6 independently biological repeats. (J) Representative action potential waveforms recorded by single‐cell patch clamp from Cx43‐KO iPSC‐CMs treated with phosphate‐buffered saline (PBS, vehicle), NAC (5 mm, 4 h) or proline (Pro, 100 nm, 24 h). Dash lines indicate 0 mV. Red dots indicate arrhythmias. (K) Bar graph to compare the percentage of cells with arrhythmia among different groups in Panel J. *n* = 31–65 cells. (L,M) Bar graphs to compare the beating rate and APD_90_ among different groups in Panel J. *n* = 15 cells. (A–M) “KO” in the figure panels refers to combined data from KO‐1 and KO‐2 analyzed in parallel. (B,C,E–H) *p* values were calculated using Mann–Whitney test, (D) unpaired two‐tailed Student's *t*‐test, (I) one‐way ANOVA followed by Tukey's multiple comparisons test, (L) Brown–Forsythe ANOVA test/Welch ANOVA test followed by Dunnett T3 multiple comparisons test, and (M) Kruskal–Wallis test followed by Dunn's multiple comparisons test. Data were shown as mean ± SEM.

Intracellular calcium homeostasis plays a prominent role in the electrical and mechanical functions of the heart [26], and the function and localization of Cx43 are essential for maintaining calcium homeostasis and normal cardiac rhythm. Therefore, we next conducted ratiometric Fura‐2 calcium imaging to assess the calcium transient properties in iPSC‐CMs (Figure 5A and Figure S8A). Consistent with the patch clamp data, we observed a greatly higher proportion of cells with arrhythmia‐like irregular calcium transients in Cx43‐KO iPSC‐CMs, when compared to WT iPSC‐CMs (Figure S8B). Cx43‐KO iPSC‐CMs also exhibited significantly higher calcium transient amplitude and peak calcium, increased maximal rising and decay rates, shortened time to peak, and prolonged transient duration 90, whereas diastolic calcium levels were comparable between WT and Cx43‐KO iPSC‐CMs (Figure 5B–H and Figure S8C–I). Moreover, western blot analysis revealed significantly lower expression levels of calcium handling–associated proteins (ryanodine receptor 2 (RYR2), L‐type calcium channel Ca_v_1.2, sodium–calcium exchanger 1 (NCX1), and sarco/endoplasmic reticulum calcium ATPase 2a (SERCA2a)) in Cx43‐KO iPSC‐CMs, along with reduction of Cx43 level (Figure S8J–O). In line with the previous study [27], the cardiac sodium channel Na_v_1.5 expression was also reduced in Cx43‐KO iPSC‐CMs (Figure S8J,P). Given that the mitochondrial calcium and ROS are closely related to mitochondrial metabolism, we further used a Rhod‐2 dye to measure the mitochondrial calcium content. We found that the mitochondrial calcium level was significantly elevated in Cx43‐KO iPSC‐CMs in comparison with WT iPSC‐CMs (Figure 5I). The observed abnormal calcium handling, decreased expression of Na_v_1.5, and calcium handling–associated proteins, and elevated mitochondrial calcium in Cx43‐KO iPSC‐CMs also gained further prominence when stimulated with ISO (Figure S8A–P and Figure 5I). Treatment of NAC or MT significantly alleviated these abnormalities in Cx43‐KO iPSC‐CMs, regardless of ISO stimulation (Figure S8A–P). Notably, NAC treatment significantly rescued the arrhythmic phenotype in Cx43‐KO iPSC‐CMs (Figure 5J,K), but did not affect beating rate or APD_90_ (Figure 5L,M). We also found that after supplementation with proline, the arrhythmic phenotype in Cx43‐KO iPSC‐CMs was greatly alleviated, accompanied by significantly reduced beating rate and longer APD_90_ (Figure 5J–M), suggesting the functional role of proline metabolism in maintaining electrophysiological stability. The faster beating rate in Cx43‐KO iPSC‐CMs was not considered to have been caused by oxidative stress, but more likely associated with the upregulated level of the *HCN2* transcript (Figure S8Q). Taken together, these results suggest that ROS‐mediated abnormal calcium handling confers the arrhythmic phenotype upon Cx43‐KO iPSC‐CMs.

### 2.7 Downregulation of the Cx43‐Interacting Protein SNAT2 Leads to Disorders of Proline Metabolism and Disturbances in Redox Balance of Cx43‐KO iPSC‐CMs

**FIGURE 6:**
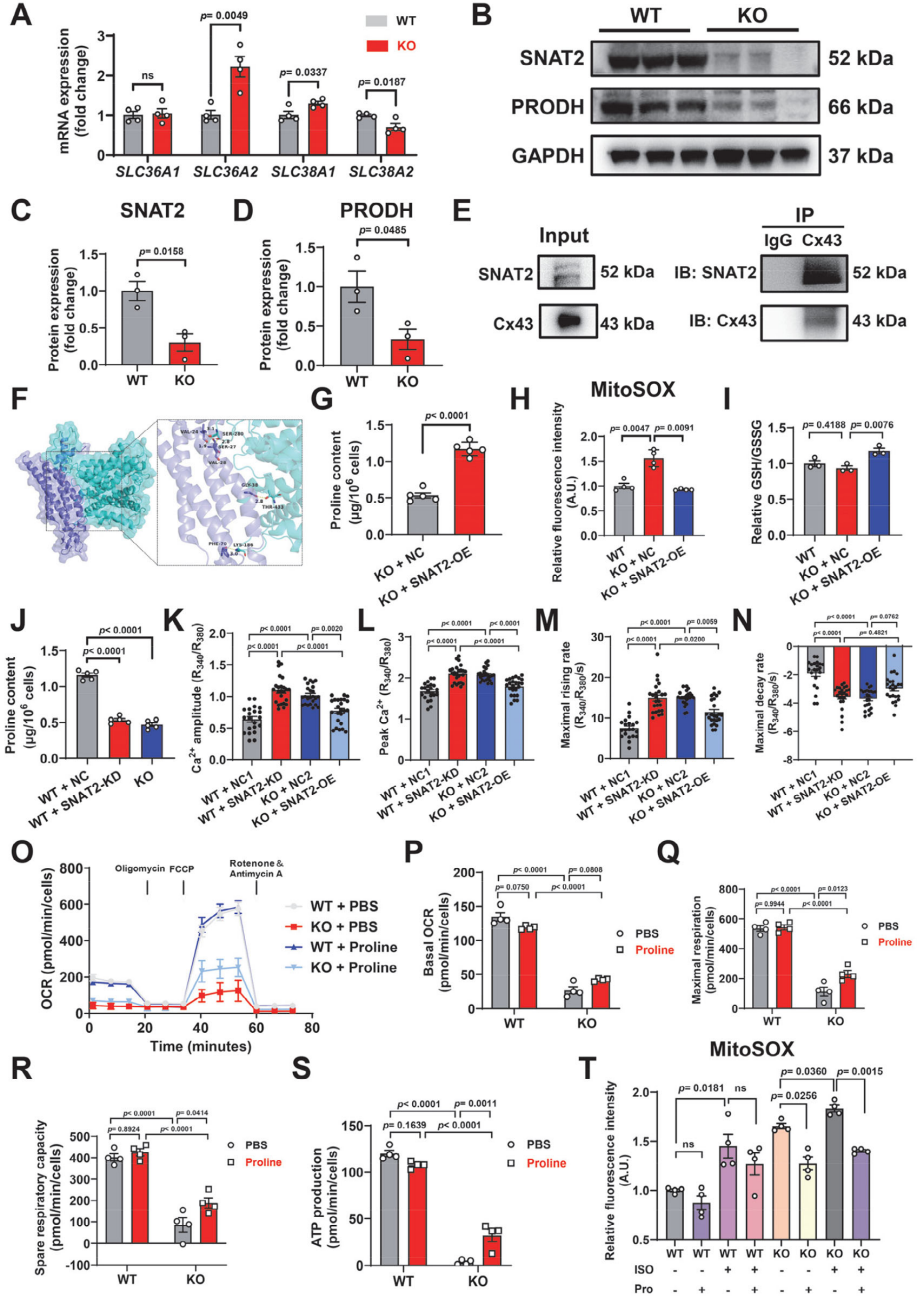
Downregulation of the Cx43‐interacting protein SNAT2 leads to disorders of proline metabolism and disturbances in redox balance of Cx43‐KO iPSC‐CMs. (A) Bar graph to compare the mRNA expression of the proline transporters (*SLC36A1*, *SLC36A2*, *SLC38A1*, and *SLC38A2*) between WT and Cx43‐KO iPSC‐CMs. *n* = 4 independently biological repeats. (B–D) Western blot analysis of the protein expression of SNAT2 (sodium‐coupled neutral amino acid transporter) and PRODH (proline dehydrogenase) in WT and Cx43‐KO iPSC‐CMs. GAPDH is used as the loading control. *n* = 3 independently biological repeats. (E) Co‐immunoprecipitation (co‐IP) assay showing that SNAT2 was detected in anti‐Cx43 immunoprecipitates in WT iPSC‐CMs. (F) Molecular docking simulation using AutoDockTools and GRAMM. Cx43 and SNAT2 are represented as slate and cyan cartoon models, respectively. (G) Measurement of proline contents through a proline assay kit in Cx43‐KO iPSC‐CMs transfected with green fluorescent protein (GFP) only (negative control, NC) (KO + NC) or SNAT2 protein labeled with GFP (SNAT2‐OE) (KO + SNAT2‐OE). OE, overexpressing. *n* = 5 independently biological repeats. (H) Bar graph to compare the mitochondrial ROS level among WT, KO + NC, and KO + SNAT2‐OE iPSC‐CMs. *n* = 4 independently biological repeats. (I) Bar graph to compare the GSH/GSSG among WT, KO + NC, and KO + SNAT2‐OE iPSC‐CMs. *n* = 3 independently biological repeats. (J) Bar graph to compare the proline content among WT iPSC‐CMs transfected with scrambled siRNA (WT + NC), WT iPSC‐CMs transfected with SNAT2 small interfering RNA (siRNA) (WT + SNAT2‐KD), and KO iPSC‐CMs. KD, knockdown. *n* = 5 independently biological repeats. (K–N) Bar graphs to compare the calcium amplitude, peak calcium, maximum rising rate, and maximum decay rate among WT + NC1, WT + SNAT2‐KD, KO + NC2, and KO + SNAT2‐OE iPSC‐CMs. *n* = 21–24 cells. (O) The diagram depicting the traces of OCR on WT and Cx43‐KO iPSC‐CMs treated with PBS (vehicle) or proline (100 nm, 24 h) after sequentially administration of 1.5 µm oligomycin, 4 µm FCCP, and 1 µm antimycin A, respectively. (P–S) Bar graphs to compare a series of fundamental parameters of mitochondrial function among different groups in Panel O, including basal OCR, spare respiratory capacity, maximal respiration, and ATP production. *n* = 2–4 independently biological repeats. (T) Measurement of mitochondrial ROS levels in WT and Cx43‐KO iPSC‐CMs with or without ISO (100 nm, 2 h) stimulation and/or proline (100 nm, 24 h) supplementation. *n* = 4 independently biological repeats. (A–D,G–T) “KO” in the figure panels refers to combined data from KO‐1 and KO‐2 analyzed in parallel. (A,C,D,G) *p* values were calculated using unpaired two‐tailed Student's *t*‐test, (H) Brown–Forsythe ANOVA test/Welch ANOVA test followed by Dunnett T3 multiple comparisons test, (I) one‐way ANOVA followed by Dunnett's multiple comparisons test, (J) one‐way ANOVA followed by Dunnett's multiple comparisons test, (K,M,N) Kruskal–Wallis test followed by Dunn's multiple comparisons test, (L,T) one‐way ANOVA followed by Tukey's multiple comparisons test, and (P–S) two‐way ANOVA followed by Tukey's multiple comparisons test. Data were shown as mean ± SEM.

Previous studies have shown that restoring proline metabolism via PRODH overexpression anaplerotically replenishes the TCA cycle and normalizes glutathione redox balance [16]. Therefore, we turned to investigating defects in proline transport, examining the expression of the amino acid transporter genes in our transcriptomic data. It has been reported that multiple transporters encoded by *SLC38A1*, *SLC38A2*, *SLC36A1*, *SLC6A20*, and *SLC38A4* participate in proline transport [28]. Among these, *SLC38A1* and *SLC38A2* had a high abundance in iPSC‐CMs, and where only the *SLC38A2* level was downregulated in Cx43‐KO iPSC‐CMs based on our RNA‐Seq data (Figure S9A). qPCR data confirmed the significantly reduced mRNA expression of *Slc38a2* in Cx43‐KO iPSC‐CMs compared to WT cells (Figure 6A). Western blot analysis revealed that the expression levels of SNAT2 and PRODH were significantly lower in Cx43‐KO iPSC‐CMs than those in WT iPSC‐CMs (Figure 6B–D). This suggested that Cx43‐KO had caused a decrease in proline transport into cardiomyocytes. It has been previously reported that Cx43 can serve as a scaffolding protein that modulating the function attributes of its interacting proteins [14]. Through co‐immunoprecipitation (co‐IP) assay and molecular docking simulation, we found that Cx43 interacted with SNAT2 in WT iPSC‐CMs (Figure 6E,F and Table S4). Moreover, immunofluorescence experiments were conducted to co‐stain Cx43 and SNAT2 in WT iPSC‐CMs. SNAT2 was detected on the cell membrane and in the cytoplasm, with marked perinuclear enrichment (Figure S9B). Importantly, co‐localization of Cx43 and SNAT2 was observed at the perinuclear and intercellular regions, further supporting the interaction between Cx43 and SNAT2 in the iPSC‐CMs (Figure S9B–D).

We next hypothesized that suppressed SNAT2‐proline‐PRODH signaling may lead to mitochondrial dysfunction and oxidative stress, thereby contributing to the arrhythmic phenotype caused by Cx43‐KO. To test this notion, we first constructed an SNAT2 overexpressing (OE) plasmid with a GFP tag. Either the empty vector plasmid (negative control, NC) or the SNAT2 plasmid was then transfected in Cx43‐KO iPSC‐CMs (Figure S9E,F). The proline content was largely restored by SNAT2‐OE in Cx43‐KO iPSC‐CMs (Figure 6G). SNAT2‐OE in Cx43‐KO iPSC‐CMs also alleviated mitochondrial oxidative stress and improved antioxidant capacity, as evidenced by significantly lower level of mitochondrial ROS and larger ratio of reduced and oxidized glutathione (GSH/GSSG, a measure of glutathione's reduced to oxidized form) (Figure 6H,I). Conversely, we applied small interfering RNA (siRNA) to genetically suppress the SNAT2 expression in WT iPSC‐CMs. Knockdown (KD) efficiency was verified by qPCR and western blot in HEK293 (human embryonic kidney 293) cells (Figure S9G–I). We found that SNAT2‐KD caused significant reduction of the proline contents in both HEK293 cells and WT iPSC‐CMs (Figure 6J and Figure S9J). The activity of ProDH was significantly reduced in WT iPSC‐CMs with SNAT2‐KD (Figure S9K). Altogether, these results indicate that Cx43 interacts with the amino acid transporter SNAT2, and Cx43 deficiency downregulates SNAT2 expression to reduce proline transport into cardiomyocytes. This leads to disorder of proline metabolism and disturbance of redox balance in Cx43‐KO iPSC‐CMs.

### 2.8 Supplementation of Proline Alleviates Oxidative Stress and Improves Mitochondrial Function

We next sought to investigate whether the proline content reduction can give rise to any functional consequence. We found that WT iPSC‐CMs with SNAT2‐KD showed markedly abnormal calcium transients, whereas SNAT2‐OE significantly ameliorated the calcium transient abnormalities in Cx43‐KO iPSC‐CMs (Figure 6K–N). Moreover, proline supplementation significantly improved the mitochondrial function in Cx43‐KO iPSC‐CMs, manifesting increased basal OCRs, maximum respiratory capacity, spare respiratory capacity, and ATP production (Figure 6O–S). When supplemented with proline, the mitochondrial ROS levels were observed to be significantly reduced in Cx43‐KO iPSC‐CMs at baseline and under ISO stimulation (Figure 6T), therefore, pointing to alleviation of oxidative stress. These results suggest that supplementation of proline alleviates oxidative stress and improves mitochondrial function.

### 2.9 Exogenous Proline Supplementation Alleviates the Proline Metabolic Dysfunction and Suppresses the Stress‐Induced Arrhythmias in Heterozygous Cardiac‐Specific Conditional Cx43‐KO Mice

**FIGURE 7:**
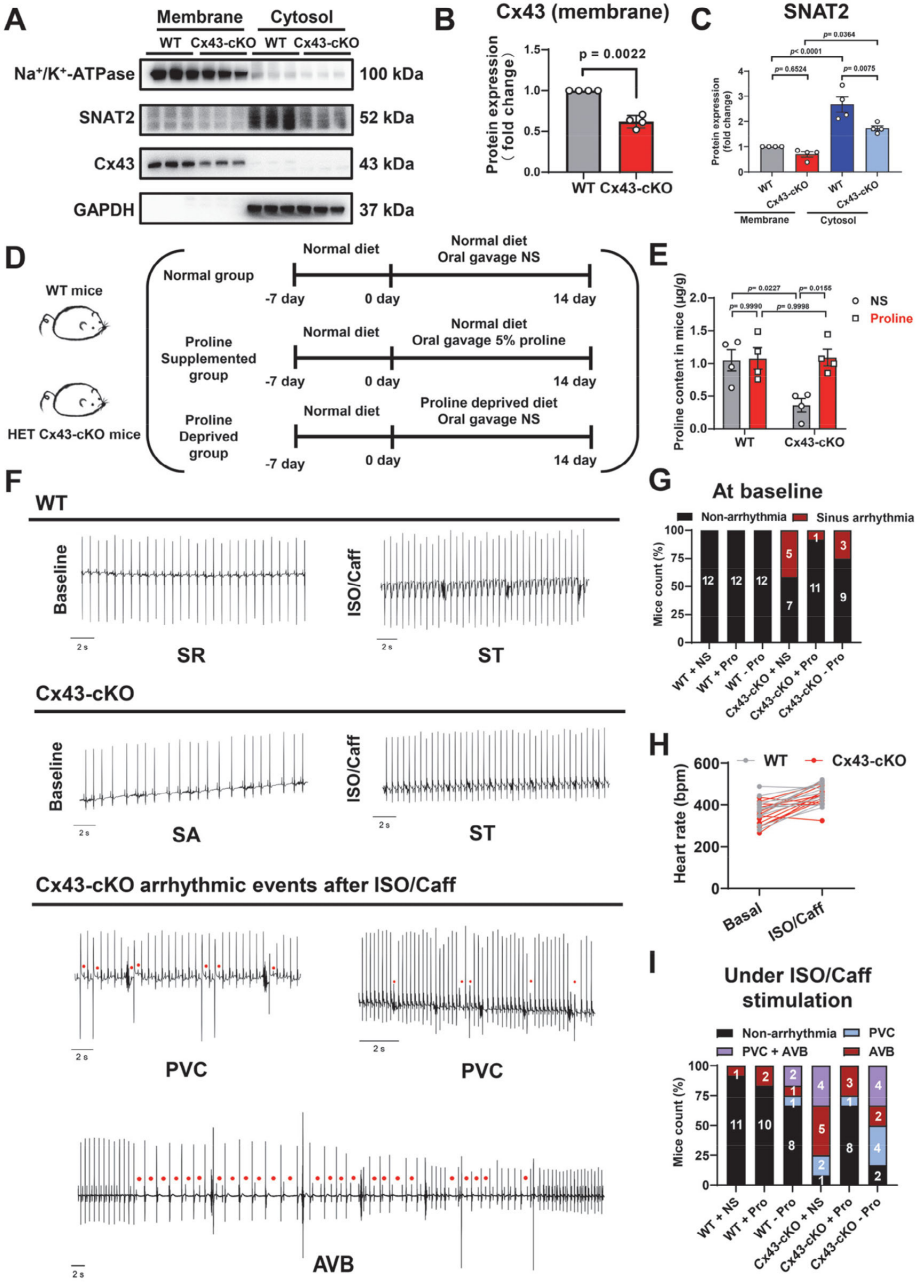
Exogenous proline supplementation alleviates the proline metabolic dysfunction and suppresses the stress‐induced arrhythmias in heterozygous cardiac‐specific conditional Cx43‐KO mice. (A–C) Western blot analysis of the protein expression of membrane and cytosol Cx43 and SNAT2 in ventricular tissues isolated from 2‐month‐old Myh6‐Cre^−^‐*Gja1* ^flox/+^ (WT) and heterozygous (HET) Myh6‐Cre^+^‐*Gja1* ^flox/+^ cardiac‐specific conditional Cx43‐KO (Cx43‐cKO) mice. Na^+^/K^+^‐ATPase and GAPDH are used as the membrane and cytosol loading controls, respectively. *n* = 4 mice. (D) Schematic representation of animal experiments. 2‐month‐old WT and HET Cx43‐cKO mice were used in the study. Normal group was received daily oral gavage of normal saline (NS); proline supplemented group was fed on normal diet and received daily oral gavage of NS supplemented with 5% proline for 14 days; proline deprived group was fed on proline‑deprived diet and received daily oral gavage of NS for 14 days. The mice were euthanized with carbon dioxide after the experiments. (E) Bar graph to compare the proline content in ventricle among 2‐month‐old WT and HET Cx43‐cKO mice with or without proline supplementation. *n* = 4 mice. (F) Representative surface electrocardiogram (ECG) recordings of 2‐month‐old WT and HET Cx43‐cKO mice at baseline and during stimulation by ISO (2 mg/kg) and caffeine (Caff, 120 mg/kg). SR, sinus rhythm; ST, sinus tachycardia; SA, sinus arrhythmia. Red dots indicate the arrhythmias. (G) Bar graph to compare the incidence of SA at baseline level among 2‐month‐old WT and HET Cx43‐cKO mice fed on normal, proline‐supplemented, or proline‐deprived diet. “Pro” denotes proline. *n* = 12 mice. (H) Line graph to compare the heart rate among 2‐month‐old WT and HET Cx43‐cKO mice with or without ISO/caffeine stimulation. *n* = 12 mice. (I) Bar graph to compare the incidence of VA (PVC, atrioventricular block (AVB), PVC + AVB) under ISO/caffeine stimulation among 2‐month‐old WT and HET Cx43‐cKO mice fed on normal, proline‐supplemented, or proline‐deprived diet. “Pro” denotes proline. *n* = 12 mice. *p* values were calculated using unpaired *t* test with (B) Welch's correction, (C) one‐way ANOVA followed by Tukey's multiple comparisons test, and (E) two‐way ANOVA followed by Tukey's multiple comparisons test. Data were shown as mean ± SEM.

To investigate the regulation role of Cx43 in VAs in vivo, cardiac‐specific conditional Cx43‐KO (Myh6‐Cre^+^‐*Gja1* ^flox/+^, hereafter named Cx43‐cKO) mice were employed (Figure S10A). In line with the previous reports [29, 30], all homozygous (HOM) Cx43‐cKO mice died during the embryonic stage, while heterozygous (HET) Cx43‐cKO had no effect on mouse survival during a 56‐day observation window (Figure S10B). WT and HET Cx43‐cKO mice at 2 months old were, therefore, used for all the in vivo functional experiments in this study. Western blot analysis revealed a significant reduction (50%–60%) of Cx43 expression in HET mouse ventricular tissues as compared to WT (Myh6‐Cre^−^‐*Gja1* ^flox/+^) (Figure S10C,D). After separating the cell membrane and cytoplasm of the isolated mouse ventricular tissues we found that Cx43 was mainly expressed on the cell membrane, and that the expression of membrane located Cx43 was significantly reduced in the HET group as compared to WT (Figure 7A,B). Meanwhile, the expression levels of SNAT2 on the cell membrane were significantly higher than that in the cytoplasm, with both membrane and the cytosol SNAT2 levels being lower in the HET group than in the WT group (Figure 7A,C). Immunofluorescence experiments were performed to co‐stain Cx43 and SNAT2 in ventricular tissues collected from WT and HET mice. Co‐localization of Cx43 and SNAT2 was detected in the WT group (Figure S11A). Compared to the WT group, both the Cx43 and SNAT2 protein levels were significantly reduced in the HET group, with an absence of Cx43 lateralization (Figure S11A–C). Reciprocal co‐IP assays using either Cx43 or SNAT2 antibody revealed the interaction between Cx43 and SNAT2 on the cell membrane and in the cytoplasm of the mouse ventricular tissues (Figure S11C). Moreover, abnormal mitochondrial structures were observed in HET mouse ventricular tissues, as evidenced by disrupted and dissolved mitochondrial cristae (Figure S12A). When supplemented with proline, the mitochondrial ultrastructure was largely improved in HET mouse ventricular tissues (Figure S12A). Western blot analysis revealed that proline supplementation significantly increased the Cx43 levels in HET mouse ventricular tissues (Figure S12B,C).

Next, the 2‐month‐old WT and HET mice were divided into three groups (normal diet group, proline‐supplemented group, and proline‐deprived group) according to whether they were gavaged with proline (Figure 7D). Impressively, the level of proline was significantly diminished in HET mouse ventricles, which was effectively restored upon oral administration of exogenous proline (Figure 7E). Proline supplementation did not affect the proline content in WT mouse ventricles (Figure 7E).

Moving forward, we sought to investigate the arrhythmic phenotypes and the antiarrhythmic effect of proline in HET mice. The in vivo electrophysiological analysis in mice comprised 3‐min recordings at baseline, and 15‐min recordings under ISO (2 mg/kg) and caffeine (120 mg/kg) stimulation. Compared to WT mice, HET mice exhibited a higher incidence of sinus arrhythmia (SA) at baseline, which could be reduced by proline supplementation (WT + normal saline (NS): 0/12; HET + normal saline: 5/12; HET + proline: 1/12) (Figure 7F,G and Figure S13A). When the mice were subjected to stimulation by ISO and caffeine, distinct differences emerged between WT and HET groups, with accelerated heart rates being noted in both groups (Figure 7H). WT mice had a low incidence of VA under ISO/caffeine stimulation, with 1 out of 12 mice displaying atrioventricular block (AVB) (Figure 7F,I and Figure S13B). In the HET group, 11 out of 12 mice displayed stress‐induced VAs. Specifically, two mice developed PVC, five mice developed AVB, and four mice developed both PVC and AVB (Figure 7F,I and Figure S13B). Notably, proline supplementation greatly reduced the incidence of VA in HET mice under ISO/caffeine stimulation, with 4 out of 12 mice exhibiting VAs (Figure S13B). Among these mice, one developed PVC and three developed AVB (Figure 7I). In contrast, proline deprivation increased the incidence of VA in WT mice under the stimulation condition (Figure S13B). Out of 12 mice, 1 developed PVC, 1 developed AVB, and 2 developed both PVC and AVB (Figure 7I). In the HET group, 10 out of 12 mice displayed stress‐induced VAs upon proline deprivation, with a higher incidence of PVC (HET + normal saline: 6/12; HET—proline: 8/12) (Figure 7I and Figure S13B). Moreover, key parameters of baseline electrocardiograms (ECGs) were analyzed and compared between WT and HET mice. The interval of QRS interval (QRS) complex was significantly prolonged in HET mice, whereas QT and corrected QT (QTc) intervals were comparable between WT and HET mice (Figure S13C–E). Proline supplementation had no significant effect on QRS complex, QT and QTc intervals in either WT or HET mice (Figure S13C–E). However, upon proline deprivation, HET mice demonstrated significant prolongation of QRS complex, QT and QTc intervals in comparison with WT mice (Figure S13C–E).

## 3 Discussion

VAs are common cardiac rhythm disturbances presented to clinical practice. Accumulating evidence has confirmed gap junction remodeling and altered connexin expression to be contributory factors to VA development [5, 6, 7]. However, the detailed molecular mechanisms for this remain to be clarified. Our study established iPSC‐CM and mouse models with Cx43‐KO, exhibiting proarrhythmic activity and disrupted proline metabolism. We further uncovered that Cx43‐KO in iPSC‐CMs exhibited a pathogenic signature of mitochondrial dysfunction and oxidative stress, resulting from reduced expression of the Cx43‐interacting protein SNAT2. Downregulation of SNAT2 caused decreased intracellular proline contents, serving as an alternative metabolic and antioxidant substrate. Elevated ROS generated from impaired mitochondria eventually promoted abnormal calcium handling and proarrhythmic activity. These findings elucidate that the deficiency of Cx43 could facilitate the development of arrhythmias through the reprogramming of proline metabolism independent of re‐entry mechanisms, and indicate that the molecular pathway of Cx43–SNAT2–ROS plays an essential role in maintaining normal cardiac rhythm (Figure S14).

As a crucial gap junction protein, deficiency of Cx43 contributes to arrhythmias through conduction heterogeneity and re‐entry [31, 32]. We showed that Cx43‐KO iPSC‐CMs exhibited fast beating rate that required more energy and antioxidant production for maintaining balance in cardiomyocytes. As in the previously reported observations in hearts of mice with ischemia/reperfusion injury stimulation [33], here impaired mitochondria dynamics and damaged OXPHOS in Cx43‐KO iPSC‐CMs were also evident. We found that the mitochondria were diminutive and abundant, while the number of mitochondrial cristae had decreased beyond KO. Similarly, under β‐adrenergic receptor agonist (ISO) stimulation, Cx43‐KO exacerbated the arrhythmic phenotype, but failed to lead to an increase in beating rate. These results indicate that in such a case mitochondria could not meet energy demands and redox balance. Through transcriptome and metabolome analyses for Cx43‐KO iPSC‐CMs, differential gene and metabolic substrates were noted as enriched in “arginine and proline metabolism,” where “l‐proline” was decreased in two different clones.

We observed enhanced calcium dynamics in the context of reduced levels of key calcium‐handling proteins in Cx43‐KO iPSC‐CMs. Our data lead us to propose that the elevated ROS in Cx43‐KO iPSC‐CMs could be critical reconciling these observations. While the total expression levels of proteins like RYR2 and SERCA2a were decreased, the function of those proteins could be alternatively regulated. A possible mechanism could be that elevated ROS can promote hyperphosphorylation or direct oxidative modification (e.g., of RYR2), which can increase channel open probability and sarcoplasmic reticulum calcium leak. This functional “hyperactivation” could effectively over‐ride the impact of their lower overall abundance, leading to the observed larger and faster calcium transients. This hypothesis is strongly supported by our key finding that antioxidant treatment (NAC or MT) significantly normalized the calcium‐handling abnormalities. Therefore, we posit that the paradoxical calcium‐handling phenotype is likely driven by an ROS‐mediated shift in the function of calcium‐handling proteins, rather than their expression levels alone. Elucidating this precise mechanism will be a major focus of our future work.

As an essential nutritional molecule, proline is one of the important nonessential amino acids in protein synthesis [15]. As nonessential amino acids can be synthesized by the human body, they are often in danger of not being granted sufficient attention [15]. However, several recent studies have revealed that proline serves as an important signaling function in the modulation of metabolic processes and immunity [34, 35]. Our study extends such an application of proline to this aspect of cardiac arrhythmias. Firstly, proline is confirmed as a safeguard against hypoxia and stress conditions [36]. While tachycardia triggered by Cx43‐KO is equivalent to equivalent cases of stress stimulation attributed to lack of nutrition or other factors, the replenishment of proline behaves as an alternative substrate for the TCA cycle and against oxidative damage in iPSC‐CMs. Oral gavage of proline thereby reduced the occurrence of VAs in HET Cx43‐KO mice. Secondly, the biosynthesis and degradation catabolism of proline are tightly regulated [15]. One recent study reported that impaired mitochondria dynamics could influence mitochondrial oxidative phosphorylation and biosynthetic selectively [37]. Mechanistically, pyrroline‐5‐carboxylate synthetase (P5CS), the rate‐limiting enzyme in the reductive synthesis of proline and ornithine, exerts strict control over the categorization of mitochondrial subpopulations [37]. Therefore, the deficiency of Cx43 in cardiomyocytes results in proline deficiency, which then subsequently impairs the mitochondrial ability against oxidative stress.

The mitochondrial dysfunction caused by Cx43 deficiency could not only lead to an energy shortage, but also generate excessive ROS. This high oxidative stress state then acts as a trigger, leading to an increased heart rate as a maladaptive stress response. However, in the long run, the energy supply cannot meet the sustained high demand of this accelerated rate. This mismatch creates a vicious cycle, where the increased heart rate further worsens the energy depletion, ultimately exacerbating the condition. We speculate that the energy deficiency is at a cost of increased ROS, which is responsible for a higher rate.

Disruption of exogenous proline uptake in immune cells via dietary restriction or deficiency of the proline transporter *SLC6A7* exacerbates colitis in mice [34]. Inhibiting proline transport through deletion of *SLC6A20* attenuates depression‐like behaviors induced by external stress in *Drosophila melanogaster* [38]. In our study, given that effective improvement of proline supplementation, there is a problem for proline transport under the deficiency of Cx43. By screening through literature reports and experimental validation, SNAT2 was selected [28], which is encoded by *SLC38A2* and enables neutral l‐amino acid symporter activity [39]. One previous study revealed that SNAT2 acts as a competitive checkpoint for the immune function of tumor cells and conventional dendritic cells (cDC1s), confirming also that cDC1s obtain glutamate through SNAT2 to improve immune activation [40]. Similarly, we showed in our study that proline content could be regulated by gene manipulation of SNAT2 in iPSC‐CMs. Overexpression of SNAT2 and supplementation of proline in Cx43‐KO iPSC‐CMs both exerted beneficial effects against oxidative stress.

Our co‐IP and immunofluorescence analyses indicate that SNAT2 is not restricted to the cell membrane, which is consistent with its regulated intracellular trafficking cycle. We propose that cytoplasmic SNAT2 signaling reflects dynamic intracellular transport of SNAT2, rather than suggesting a nonmembrane function. A substantial intracellular pool of SNAT2 is known to reside in cytoplasmic vesicles and can be rapidly mobilized to the plasma membrane in response to metabolic or signaling stimuli. It has been recently reported that UBE2C‐mediated monoubiquitination regulates the membrane abundance of SNAT2 [41], thereby regulating amino acid uptake in response to metabolic demand, which supports that Cx43 may influence amino acid transport and metabolic homeostasis in cardiomyocytes by modulating SNAT2 trafficking dynamics rather than its static localization.

The formation of intercellular gap junctions by Cx43 to mediate cell communication has long been a research hotspot for connexins. However, recent studies have revealed that Cx43 possesses a broader range of biological functions beyond its canonical role in gap junction‐mediated intercellular communication [14]. Cx43 not only presents in the cell membrane but also performs biological functions within the cell. In particular, it can serve as a scaffolding protein that modulates the function of the proteins such as PKP2 and Na_v_1.5, which it interacts with [42, 43]. Notably, we found a molecular pathway of Cx43–SNAT2 in the development of VAs. Cx43 deficiency leads to VAs through SNAT2‐mediated proline metabolic dysfunction. Insufficient proline impairs mitochondrial function and induces oxidative stress, culminating in calcium dyshomeostasis and arrhythmias.

Nakagami et al. reported that arrhythmia susceptibility depends on the degree and pattern of Cx43 reduction [10]. Cx43 levels gradually decreased with age, and severe conduction slowing and inducible VT were only observed after >80% reduction, whereas an ∼40% decrease produced minimal conduction changes [12]. This temporal model resembles our systems at different stages: the early stage corresponds to our heterozygous Cx43‐cKO mice (30%–50% reduction), whereas the late stage more closely resembles our Cx43‐KO iPSC‐CMs (80%–90% reduction). At the late stage, when Cx43 expression is profoundly reduced, heterogeneous gap junction loss likely promotes conduction heterogeneity and facilitates re‐entry. However, at the early stage, when Cx43 expression is only partially reduced, additional mechanisms may predominate. Our findings suggest that metabolic remodeling, mitochondrial dysfunction, oxidative stress, and calcium‐handling abnormalities can create arrhythmogenic vulnerability even before conduction slowing becomes prominent. Notably, the methods of arrhythmia induction differ substantially between the Nakagami et al. study and our study. The Nakagami et al. study relied on programmed electrical stimulation to probe re‐entry‐based arrhythmias, while we used an ISO/caffeine stimulation, which preferentially unmasks the calcium‐driven triggered activities (TAs). The methodological distinction may contribute to the observed differences in arrhythmia inducibility at moderate levels of Cx43 reduction.

There are multiple pathogenic mechanisms of VA, which may not necessarily be accompanied by the reduced Cx43 expression. VAs can arise from ion channel dysfunction without changes in Cx43. Cardiac channelopathies, for example, long QT syndrome, short QT syndrome, Brugada syndrome, and catecholaminergic polymorphic ventricular tachycardia, are primarily driven by ion channel mutations, but not gap junction remodeling. In the arrhythmogenic cardiomyopathy mouse model, Cx43 levels are preserved, but impaired trafficking and mislocalization of Cx43 at the intercalated discs are observed, which correlate with the manifestation of VA [44]. In the mdx mouse model of Duchenne muscular dystrophy, the cardiac Cx43 protein expression is significantly upregulated and exhibits prominent lateral redistribution away from the intercalated discs. Genetically restoring Cx43 expression to physiological levels and reducing its lateral mislocalization have been shown to mitigate key pathological features, including cellular stress responses and the propensity for arrhythmias [45]. It is important to note that the molecular mechanism and therapeutic strategy described in our study are specifically applicable to Cx43‐deficiency–associated VAs.

Our study has several limitations. Firstly, it is unclear why Cx43 deficiency caused tachycardia. Based on the increased mRNA level of *HCN2* in the transcriptome data, this may be related to the transcriptional inhibition of Cx43, which requires further investigation. Secondly, we did not investigate the detailed Cx43 activity by phosphorylation, lateralization/internalization, and the impact these have on the interaction with SNAT2 and resultant proline metabolism, and elucidating the precise mechanisms will be a major focus of our future work. Thirdly, tissue‐level conduction mapping and measurement of proline content in human tissue were not conducted in our study. In future work, applying the optical mapping technique to our model would provide important evidence to support the re‐entrant mechanism of VA, and incorporating tissue‐level metabolic profiling in clinical cohorts would offer definitive translational validation. It would be meaningful to apply the optical mapping technique to our model in future work. Finally, our use of a constitutive Myh6‐Cre driver represents another limitation. In this model, Cre recombinase is active during embryonic cardiac development, resulting in prenatal Cx43 deletion in both heterozygous and homozygous mice. Consequently, developmental remodeling or compensatory adaptations cannot be excluded when interpreting the adult phenotypes. An inducible cardiomyocyte‐specific Cre system would provide temporal control of Cx43 deletion and more precisely evaluate the effects of adult‐onset Cx43 reduction or loss without confounding developmental influences. Future studies employing inducible models will be essential to validate and extend the findings.

In conclusion, we anticipate that Cx43 deficiency facilitates the VA formation through SNAT2‐mediated proline metabolism reprogramming independent of re‐entry mechanisms. Our findings suggest that the molecular pathway of Cx43–SNAT2–ROS plays an essential role in maintaining the normal cardiac rhythm. Targeting proline metabolism could aid in the development of new effective treatment strategies for Cx43 deficiency–associated VAs.

## 4 Methods

### 4.1 Screen Pathogenic Genes for Ventricular Arrhythmias

VAs include PVC, Vf, VT, and VF. The pathogenic targets of VAs were searched using Online Mendelian Inheritance in Man (OMIM, http:// omim.org/) [46] and DisGeNET databases [47]. The intersection of PVC‐, Vf‐, VT‐, and VF‐related genes was imported into the Search Tool for the Retrieval of Interacting Genes (STRING, v12.0) to generate a PPI network [48]. Results were visualized using Cytoscape software (v3.8.2), with CytoHubba as a plugin to identify hub nodes by ranking nodes in a co‐expression network based on their network characteristics. In this study, we employed the CytoHubba plugin with algorithms including Stress, MNC, Degree, Radiality, and Betweenness to identify hub genes [49].

The GSE33165 database contains the mRNA expression profiles of ion channels, calcium‐handling proteins, and transcription factors, comparing individuals with VAs to those with normal cardiac rhythms. Through the Upset package, we identified the intersection of genes obtained from the CytoHubba algorithm and those in GSE33165. *GJA1*, *SCN5A*, *CACNA1C*, *CAV3*, *KCNH2*, and *RYR2* were considered pathogenic genes associated for VAs.

### 4.2 Generation of Cx43‐KO iPSC Lines by CRISPR/Cas9

An sgRNA targeting the exon 1 of *GJA1* was designed using the CRISPR online design tool (http://crispr.mit.edu). The guide RNA (gRNA) sequence of *GJA1* is shown in Figure 2A. The gRNA‐expressing plasmid was generated as previously reported. 1 × 10^5^ WT iPSCs were seeded in 12‐well plates to 80% confluence. Briefly, the recombinant plasmid was transfected into WT iPSCs using Lipofectamin 3000 Transfection Reagent (Invitrogen, L3000001) according to the manufacturer's instructions. The puromycin‐resistant clones were screened within mTeSR1 (STEMCELL Technologies, 85850), supplemented at a concentration of 4 µg/mL puromycin (Gibco, A1113803). After 48‐h treatment, the resistant clones were picked and verified by DNA sequencing (Sangon Biotech). Experiments were designed to include two independently generated Cx43‐KO iPSC clones (KO‐1 and KO‐2). For analyses intended to demonstrate clone‐independent reproducibility, data from KO‐1 and KO‐2 were pooled into a combined KO group, resulting in a higher total *n* relative to WT.

### 4.3 Culture and Maintenance of iPSCs

The iPSCs were cultured in feeder‐free mTeSR1 media on Matrigel‐coated plates at 37°C with 5% (vol/vol) CO_2_. The media were changed daily and iPSCs were passaged every 3–4 days using Accutase (STEMCELL Technologies, 07920), then resuspended and seeded in mTeSR1 containing 10 µm Y27632 ROCK inhibitor (Selleck, S1049).

### 4.4 Karyotyping

Chromosome analysis by G‐banding was achieved using iPSCs at passage 20 at the Prenatal Diagnosis Center of the Sir Run Run Shaw Hospital, Zhejiang University School of Medicine. At least 20 metaphase cells were analyzed at the 300–400 band level.

### 4.5 ALP Staining

ALP staining was performed using a VECTOR Blue Alkaline Phosphatase Substrate Kit (Vector Laboratories, SK‐5300) following the manufacturer's instructions.

### 4.6 Teratoma Formation

5‐week‐old female Non‐obese Diabetic/severe combined immune deficency mice were purchased (GemPharmatech) and utilized for teratoma formation assay. Approximately 1 × 10^6^ iPSCs at passage 20 were digested using Accutase and suspended in 0.5 mL mTeSR1 supplemented with 10 µm Y27632 ROCK inhibitor. After mixed with 0.5 mL Matrigel, cells were injected into the armpits and back of the mice. 4–6 weeks after cell delivery, teratomas were dissected and fixed with 4% paraformaldehyde (PFA) (Beyotime Biotechnology, P0099) for hematoxylin and eosin (H&E) staining.

### 4.7 Cardiac Differentiation

The iPSC‐CMs were generated using a 2D monolayer differentiation protocol. Briefly, ∼10^5^ undifferentiated iPSCs were dissociated and replated into Matrigel‐coated 6‐well plates. These iPSCs were cultured, expanded to 85% confluence, and then treated for 2 days with 6 µm CHIR99021 (Axon Medchem, 1386) in RPMI 1640 (Gibco, C11875500BT) plus a B27 supplement without insulin (Gibco, A1895601) (RPMI+B27‐insulin) to activate the Wnt signaling pathway. On day 2, cells were placed in RPMI+B27‐insulin with CHIR99021 removal. On days 3–4, cells were treated with 5 µm IWR‐1 (Millipore, 681669) to inhibit the Wnt signaling pathway. On days 5–6, cells were removed from IWR‐1 treatment and placed in RPMI+B27‐insulin. From day 7 onward, cells were placed and cultured in RPMI 1640 and B27 supplement with insulin (Gibco, 17504044) (RPMI + B27 + insulin) until beating was observed. Cells were glucose‐starved for 3 days with RPMI + B27 + insulin for purification. Cardiomyocytes obtained 30–40 after cardiac differentiation were utilized for downstream functional assays.

### 4.8 FACS Analysis of iPSC‐CMs

Monolayer iPSC‐CMs were dissociated into single cells using TypleE for 15 min at 37°C. The cells were pelleted and fixed with 2% PFA for 10 min at room temperature. At each step, samples were washed with Dulbecco's phosphate‐buffered saline (DPBS) containing 5% fetal bovine serum (FBS) and re‐centrifuged to remove residual reagents. The cells were stained with TNNT2 (Abcam, ab8295, 1:200) at room temperature for 30 min and washed as above. They were and then labeled with AlexaFluor 488‐conjugated secondary antibody (Abcam, ab150113, 1:200) for 30 min at room temperature. Detection was performed using a CytoFLEX LX flow cytometer following the manufacturer's instructions.

### 4.9 Immunofluorescent Staining

Immunofluorescence analysis was divided into cell staining and tissue staining. For cell staining, iPSC‐CMs were fixed with 4% PFA for 10 min, permeabilized with 0.1% Triton X (Sangon Biotech, A110694) for 10 min at room temperature and blocked with 3% bovine serum albumin (BSA) (Sigma–Aldrich, A1933) for 1 h. For the staining of paraffin‐embedded tissue, the slices underwent deparaffinization, antigen retrieval, and marking with a histochemical pen to delineate regions of interest. The cells or tissues were subsequently stained with appropriate primary antibodies and AlexaFluor‐conjugated secondary antibodies (Life Technologies). Nuclei were stained with 4',6‐diamidino‐2‐phenylindole (Roche Diagnostics, 1023276001, 1 µg/mL) or Hoechst (Solarbio, HOE 33258). The primary antibodies were OCT4 (Cell Signaling Technology, 2750S, 1:200), NANOG (Santa Cruz Biotechnology, sc‐33759, 1:200), SSEA‐4 (Abcam, ab16287, 1:200), SOX2 (Abcam, ab171380, 1:200), TNNT2 (Abcam, ab8295, 1:500), α‐Actinin (Cell Signaling Technology, 6487P, 1:100), Cx43 (Santa Cruz Biotechnology, sc‐271837, 1:200; Abcam, ab11370, 1:500), and SNAT2 (Santa Cruz Biotechnology, sc‐514037, 1:200, Invitrogen, PA5‐106786, 1:200). Secondary antibodies were AlexaFluor 647(Abcam, ab150079, 1:200), AlexaFluor 594 (Abcam, ab150080, 1:500; Abcam, ab150108, 1:500), and AlexaFluor 488 (Abcam, ab150113, 1:500; Invitrogen, A11008,1:500). The primary antibodies of α‐tubulin in microtubule network were stained with Tubulin‐Tracker Deep Red (Sigma–Aldrich, T34077) and β‐tubulin was stained with Tubulin‐Tracker green staining kit (Beyotime, C22135). Images were obtained with 60× objective on a confocal microscope (Nikon, A1) using NIS‐Elements AR software (Nikon).

### 4.10 qPCR

Total RNA isolation was performed using TRIzolReagent (Life Technologies, 15596018CN). complementary DNA was obtained using the 4 × EZscript Reverse Transcription Mix II (EZB, EZB‐RT2GQ). qPCR was conducted using SYBR Green PCR Master Mix (Takara, RR420A). The mRNA expression values were normalized to *GAPDH*. The sequences of primers are listed in Table S5.

### 4.11 Western Blot

The iPSC‐CMs were detached with TrypLE and then pelleted at 1000 rpm for 5 min at 4°C. After washing with phosphate‐buffered saline (PBS), pellets were re‐suspended in 50–100 µL lysis buffer. The ventricular tissues of the mice were dissected and homogenized using a tissue disruptor in lysis buffer. Lysates were placed on ice for 30 min and supernatants collected after centrifuging at 12 000 rpm for 15 min. Membrane proteins were extracted using a Mem‐PER Plus Membrane Protein Extraction Kit (Thermo, 89842) according to the manufacturer's instruction. Protein concentrations were measured via a BCA kit (Pierce, 23227). Western blot was performed using standard protocol with the following antibodies: Cx43 (Abcam, ab11370, 1:1000), DRP1 (Proteintech, 12957‐1‐AP, 1:500), phospho‐DRP1 (Ser616) (Cell signaling, 3455S, 1:1000), PRODH (Proteintech, 22980‐1‐AP, 1:500), SNAT2 (Santa Cruz Biotechnology, sc‐514037, 1:500), Na^+^/K^+^‐ATPase (Abcam, ab7671, 1:1000), Na_v_1.5 (Alomone labs, ASC005, 1:500), Ca_v_1.2 (Abcam, ab84814, 1:1000), NCX1 (Proteintech, 55075‐1‐AP, 1:1000), SERCA2a (Santa Cruz Biotechnology, sc‐53010, 1:200), and GAPDH (Abmart, M200006, 1:5000). Intensity values for each band were determined as the integrated density (sum of pixel values) within a fixed area using Quantity One software (Biorad).

### 4.12 Patch Clamp Recordings from iPSC‐CMs

The iPSC‐CMs were mechanically and enzymatically dissociated to obtain single cells. These were seeded on Matrigel‐coated glass coverslips. Cells with spontaneous beating were selected and action potentials were recorded using an EPC‐10 patch clamp amplifier (HEKA). Continuous extracellular solution perfusion was achieved using a rapid solution exchanger (Bio‐logic Science Instruments, RSC‐200). All signals were acquired using PatchMaster software (HEKA), filtered at 1 kHz and digitized at 10 kHz. Data analyses were performed using Igor Pro (Wavemetrics) and GraphPad Prism (GraphPad Software). A TC‐344C dual channel heating system (Warner Instruments) was used to maintain the temperature at 35.5°C –37°C. Tyrode's solution was used as the external solution containing 140 mm NaCl, 5.4 mm KCl, 1 mm MgCl_2_, 10 mm glucose, 1.8 mm CaCl_2_, 1.0 mm Na‐pyruvate, and 10 mm N‐2‐Hydroxyethylp‐iperazine‐N‐2‐Ethane Sulfonic Acid (pH 7.4 with NaOH). The internal solution contained 140 mm KCl, 5.0 mm NaCl, 10 mm HEPES, 5 mm Mg‐ATP, and 5 mm Ethylene Glycol Tetraacetic Acid (pH 7.2 with KOH). Key action potential parameters were quantified including MDP, overshoot, APA, APD_50_, APD_90_, *V*_max_, and beating rate. Ventricular‐like iPSC‐CMs were distinguished based on action potential morphology and action potential parameters, which exhibited a clear plateau phase, larger APA and *V*_max_ values, more negative MDP values, APD_30‐40_/APD_70–80_ > 1.5, and APD_90_/APD_50_ ≤ 1.3. Beating rate was calculated from interspike intervals measured from spontaneous action potentials over a stable 10‐s window. Spontaneously beating iPSC‐CMs were recorded at physiological temperature under steady‐state conditions for at least 30–60 consecutive beats. Arrhythmia‐like events were defined as follows: early after depolarization (EAD) was defined as abnormal depolarization interruptions or oscillatory depolarizations occurring during phase 2 or 3 of repolarization; delayed after depolarization (DAD) was defined as subthreshold oscillatory depolarizations arising after complete repolarization (phase 4); TA was defined as extra action potentials triggered by preceding EADs or DADs; APD alternan was defined as beat‐to‐beat alternation in APD (long–short–long–short pattern) over consecutive cycles. The arrhythmia‐like events observed in our Cx43‐KO iPSC‐CM model manifested as DAD, and the outcome was expressed as the percentage of cells exhibiting arrhythmia relative to the total number of cells analyzed. Experiments included two independently generated Cx43‐KO iPSC clones (KO‐1 and KO‐2). Because the Cx43‐KO iPSC‐CMs represented a newly established pathological model with previously uncharacterized electrophysiological phenotypes, a larger number of cells were recorded to assess the action potential characteristics of the Cx43‐KO iPSC‐CMs. In contrast, the WT iPSC‐CMs have been extensively characterized in our prior studies, and exhibit stable and reproducible electrophysiological properties, a moderate number of patch clamp recordings was therefore sufficient to determine the action potential characteristics of the WT iPSC‐CMs. For analyses aimed at demonstrating clone‐independent reproducibility, data from KO‐1 and KO‐2 were pooled into a combined KO group, resulting in a higher total *n* relative to WT.

### 4.13 MEA

For cell preparation, a 20 µL droplet of coating solution (Matrigel) was applied to the area of the electrodes of the MEA probes (Multi Channel Systems, 60MEA200/30iR‐Ti‐gr), which were then incubated at 37°C in 5% CO_2_ for at least 1 h. The iPSC‐CMs were subsequently dissociated from the 6‐well plates using TrypLE, and reseeded onto MEA probes at a density of 1–1.5 × 10^5^ cells with 20 µL bead of cell suspension per well. After incubation with 5% CO_2_ at 37°C for 1 h to promote adhesion, each MEA probe was filled with culture media to a final volume. The culture medium was changed after 2 days, and then every 2 or 3 days throughout 5–7 days of culturing period. Field potentials were recorded from spontaneously beating iPSC‐CMs using the MEA2100 data acquisition system (Multi Channel Systems) with sampling at 10 kHz. All experiments were performed at 37°C and begun after a 20‐min equilibration period. The data were analyzed using Cardio 2D^+^ software (Multi Channel Systems). Steady‐state parameters were averaged.

### 4.14 RNA Sequencing and Bioinformatic Analysis

The transcriptome sequencing and bioinformatic analysis were conducted by OE Biotech Co. Ltd. (Shanghai, China). The clean reads were mapped to the reference genome using HISAT2. FPKM was calculated for each gene and the read counts of each were obtained by HTSeq‐count. PCA was performed using R (v 3.2.0) to evaluate the biological duplication of samples. Differential expression analysis was performed using the DESeq2. *Q* value < 0.05 and foldchange > 2 or foldchange < 0.5 was set as the threshold for significantly DEGs. Based on the hypergeometric distribution, GO, KEGG pathway, and analysis of DEGs were performed to screen the significant enriched term using R (v3.2.0), respectively. Gene set enrichment analysis was performed using GSEA software.

### 4.15 Widely Targeted Metabolite Profiling and Bioinformatic Analysis

The metabolome analysis was performed by Wuhan Maiwei Biotechnology Co. Ltd. The iPSC‐CMs samples were collected according to the manufacturer's standard protocol for subsequent liquid chromatography‐mass spectrometry. In our study, differential metabolites (DEMs) were determined by VIP (VIP > 1) and *p* value (*p* < 0.05, Student's *t* test). VIP values were extracted from orthogonal partial least‐squares‐discriminant analysis (OPLS‐DA) results, which also contained score plots and permutation plots generated using R package MetaboAnalystR. The data were log transform (log_2_) and mean centering before OPLS‐DA. In order to avoid overfitting, a permutation test (200 permutations) was performed. DEMs were annotated and mapped to using the KEGG database. Pathways with significantly regulated metabolites mapped to were then fed into MSEA (metabolite sets enrichment analysis), their significance determined by hypergeometric test's *p* values.

### 4.16 Transmission Electron Microscopy

For cell experiments, iPSC‐CMs were dissociated with Tripsin–Ethylenediaminetetraacetic Acid (Gibco, 25200072), scraped into a 1.5‐mL microcentrifuge tube, centrifuged, and then fixed with 2.5% glutaraldehyde (Sigma–Aldrich, G5882) in 0.1 m phosphate buffer overnight at 4°C. For animal experiments, fresh ventricular tissues measuring approximately 1 mm^3^ were collected from each experimental group. Then samples fixed with 2.5% glutaraldehyde (Sigma–Aldrich, G5882) in 0.1 m phosphate buffer overnight at 4°C. Specimens were post‐fixed with 1% osmium tetroxide (OsO_4_) in phosphate buffer and subsequently dehydrated through a graded ethanol series with each step being maintained for 15–20 min. The samples were then transitioned to absolute acetone for 20 min. For resin infiltration, specimens were sequentially immersed in a 1:1 mixture of absolute acetone and spur resin mixture for 1 h at room temperature, followed by a 1:3 acetone–resin mixture for 3 h, then finally transferred to spur resin mixture overnight. The infiltrated specimens were embedded in spur resin within 1.5 mL tubes, polymerized at 70°C for more than 9 h, and sectioned using a LEICA EM UC7 ultratome. Sections were then stained with uranyl acetate and alkaline lead citrate for 5–10 min. Pictures were captured using a transmission electron microscope (Hitachi, Model H‐7800).

### 4.17 Measurement of Cellular and Mitochondrial ROS

For cellular or mitochondrial ROS measurement, iPSC‐CMs were treated with 5 µm CellROX (Life Technologies, C10492) or 5 µm MitoSOX (Life Technologies, M36008) at 37°C for 20 min, followed by three washes. Analysis was performed using a CytoFLEX LX flow cytometer. Data were analyzed with FlowJo (FlowJo V10, LLC) by measuring the mean fluorescence intensity of each sample.

### 4.18 Mitochondrial Copy Number Quantification

Mitochondrial DNA and genomic DNA were extracted using the TIANamp Genomic DNA Kit (Tiangen) according to the manufacturer's instructions. The copy numbers of mtDNA and nuclear DNA (nDNA) were quantified using TB Green Premix Ex Taq (Takara) on an ABI Prism 7500 system. Primers specific for ND1, ND5, SLCO2B1, and SERPINA1 were used to quantify mtDNA and nDNA (Takara, 7246), respectively. The difference in the 2*ΔCt* values for the ND1/SLCO2B1 and ND5/SERPINA1 pairs was determined in the same manner, with the average of the two values being used as the mtDNA copy number.

### 4.19 Mitochondrial Morphological Analysis

Cells were stained with 1 µm MitoTracker Red FM (Life Technologies, M36008) in RPMI 1640 medium without phenol red (Gibco, 11835030) for 20 min at 37°C, followed by three washes. Images were captured using a 60× objective on a confocal microscope (Nikon, A1) with NIS‐Elements AR software (Nikon). The images were then analyzed with the Mitochondrial Analyzer plugin in Fiji.

### 4.20 Measurement of Mitochondrial Membrane Potential

For mitochondrial membrane potential measurement, cells were stained with TMRE (MCE, HY‐D0985A) at 37°C for 20 min under light‐protected conditions. After staining, the cells were washed three times and analyzed using a CytoFLEX LX flow cytometer. Data were analyzed with FlowJo by measuring the mean fluorescence intensity of each sample.

### 4.21 Mitochondrial Stress Analysis

Real‐time OCRs were measured using an XFe96 extracellular flux analyzer (Seahorse Bioscience). Cells were seeded at 8 × 10^4^ cells per well in XFe96 cell culture microplates (Seahorse Bioscience) and incubated for 5–7 days. OCRs were measured in Seahorse assay medium (10 mm glucose, 10 mm pyruvate, and pH 7.4). Different mitochondrial respiratory states were assessed through the sequential addition of compounds that modulate or inhibit mitochondrial respiration in distinct ways.

First, the “basal OCR” was measured to assess cellular respiration under normal physiological conditions. Then, oligomycin (1.5 µm) was added to determine mitochondrial “proton leak” and “ATP‐linked OCR.” Following this, the uncoupling agent carbonyl cyanide 4‐(trifluoromethoxy) phenylhydrazone (FCCP; 4 µm) was added to obtain the maximum respiratory rate. The difference between the maximum capacity and basal OCR indicates the “reserve capacity” of the electron transport chain. Finally, a combination of rotenone (0.5 µm) and antimycin A (0.5 µm) was added to inhibit the activity of complexes I and III, respectively, in order to measure nonmitochondrial OCR and correct for nonmitochondrial sources of OCR.

### 4.22 Measurement of Mitochondrial Calcium Content

Mitochondrial calcium concentration was measured with 5 µm Rhod‐2 AM (Invitrogen, R1245MP). Cells were incubated for 15 min at room temperature in the dark. After washing twice with preheated RPMI 1640 medium without phenol red, iPSC‐CMs were incubated in imaging buffer for 30 min prior to imaging. Images were captured using a 63× objective on a Leica TCS SP8 LSCM (Leica Microsystems CMS GmbH, Mannheim, Germany) and analyzed with Fiji.

### 4.23 Cell Viability Assay

The iPSC‐CMs were plated into a 96‐well plate with 4.5 × 10^5^ cells per well. After drug treatment, cell viability assay was conducted using the Cell Counting Kit‐8 (CCK‐8) kit (Beyotime, C0038) following manufacturer's instructions. Absorbance was measured using a microplate reader (BioRad iMark Microplate Reader) at 450 nm.

### 4.24 Calcium Imaging

The iPSC‐CMs grown on coverslips were loaded with RPMI 1640 medium without phenol red, supplemented with 5 µm Fura‐2 AM (Invitrogen, F14185), for 30 min in the dark at room temperature. After washing twice with prewarmed RPMI 1640, the iPSC‐CMs were immersed in imaging buffer for 30 min before imaging experiments. For imaging, iPSC‐CMs were placed in a chamber equipped with a temperature controller, under constant perfusion of 37°C imaging buffer. Calcium signaling was made by recording the fluorescence of cells using an Ultra High Speed Wavelength Switcher (Lambda DG‐4, Sutter Instruments) with a CCD camera (Zyla, Andor) mounted on an inverted microscope (Eclipse Ti, Nikon). Fluorescent signals were obtained upon excitation at 340 nm (F_340_) and 380 nm (F_380_). The amplitude of a calcium transient was defined as the ratio of *F*_340_/*F*_380_.

### 4.25 Co‐IP

The co‐IP assay was conducted utilizing a pierce classic magnetic IP/Co‐IP kit (Thermo Scientific, 88804). The iPSC‐CMs and mouse ventricular tissues were first lysed and lysates then incubated with the primary antibodies (Cx43 (Abcam, ab11370, 1:1000) and SNAT2 (Santa Cruz Biotechnology, sc‐514037, 1:500)) at 4°C overnight. The antigen/antibody complexes were bound to protein A/G magnetic beads at room temperature for 1 h and subsequently washed with IP lysis/wash buffer. The antigen/antibody complexes were eluted for subsequent protein separation by gel electrophoresis.

### 4.26 Molecular Docking

The X‐ray crystal structures of *GJA1* (7XQF) were retrieved from the Protein Data Bank. The structures of SNAT2 were generated using SWISS‐MODEL. To ensure the accuracy of the docking results, the protein was prepared using the AutoDockTools (1.5.7), with water molecules manually eliminated from the protein and the polar hydrogen added. Docking Web Server (GRAMM) was used for protein–protein docking. The resulting protein–protein complex was also manually optimized by removing water and adding polar hydrogen using AutoDockTools (1.5.7). Protein–protein interactions were finally predicted and the protein–protein interaction figure was generated by PyMOL. The Cx43 protein is represented as a slate cartoon model and the SNAT2 protein is shown as a cyan cartoon model. Their binding sites are shown as the corresponding colored stick structure.

### 4.27 Genetic Manipulation of SNAT2 in iPSC‐CMs

SNAT2 siRNA was purchased from GenePharma and transfected into iPSC‐CMs using the Lipofectamine RNAiMAX (Invitrogen, 13778075) according to the manufacturer's instructions. The culture medium was replaced 12 h after transfection. A significant reduction in SNAT2 expression was observed after 3 days of culture.

The human SNAT2‐tagged Open reading frame clone (Origene, RG201892) was obtained from Origene and transfected into iPSC‐CMs using the Lipofectamine 3000 Transfection Reagent (Invitrogen, 3153034) according to the manufacturer's instructions. The culture medium was replaced 12 h after transfection, and a marked increase in SNAT2 expression was confirmed after 3 days by immunofluorescence analysis.

### 4.28 Measurement of Proline Content

The proline content measurement was conducted using a commercial kit (Solarbio, BC0295). The iPSC‐CMs and mouse ventricular tissues were processed according to the manufacturer's protocol. The samples were placed in boiling water for 10 min followed by cooling and supernatant extraction. The test reagents were then added, and the mixture incubated in boiling water for 30 min. Absorbance at 520 nm was subsequently measured using a spectrophotometer with proline content normalized to the sample mass.

### 4.29 Measurement of ProDH Activity

The ProDH activity measurement was conducted using a commercial kit (Solarbio, BC4160). According to the manufacturer's protocol, iPSC‐CMs samples were processed. The extraction solution was supplemented, and the mixture was gently vortexed for 30 min on ice. Subsequently, the supernatant was isolated by centrifugation at 15,000 × *g* for 20 min at 4°C, followed by incubation at 37°C for 5 min. Absorbance at 600 nm was subsequently measured using a spectrophotometer, with ProDH activity being normalized to the sample mass.

### 4.30 Detection of GSH/GSSG

To determine antioxidant capacity under various conditions, the GSH/GSSG was measured using a commercial kit (Abcam, ab138881). Briefly, cell lysates were subjected to high‐speed centrifugation to collect the supernatant. This supernatant was combined with an equal volume of assay reagent and the mixture incubated at room temperature for 1 h. Fluorescence intensity was then measured at 520 nm. Based on a standard curve, the concentrations of GSH and GSSG were quantified and the GSH/GSSG calculated.

### 4.31 Separation of Membrane and Cytosolic Proteins

Membrane and cytosolic proteins were extracted using a commercially available kit (Mem‐PER Plus, Thermo Scientific, 89842). The mouse ventricular tissue (40 mg) was washed, minced, and homogenized in permeabilization buffer. After incubation at 4°C for 10 min with agitation, the sample was centrifuged at 16,000 × *g* for 15 min at 4°C. The cytosolic supernatant was collected and the pellet resuspended in solubilization buffer, homogenized, and then incubated at 4°C for 30 min with agitation. Following centrifugation at 16,000 × *g* for 15 min at 4°C, the supernatant containing membrane proteins was collected for analysis.

### 4.32 Experimental Animals and Protocols

All animal experiments were followed the Institutional Animal Care Guidelines and were approved by the Animal Experimentation Ethics Committee of Zhejiang University (20240720‐38). The floxed Cx43 model (B6.129S7‐*Gja1* ^tm1Dlg^/J (JAX Stock #008039)) was generously provided by Dr. Yu Nie from Fuwai Hospital. Given that HOM mice for the *Gja1* ^tm1Dlg^/J knockout died at birth, HET mice were used for subsequent experimental investigations. The flox/+ mice were crossed with Myh6‐iCre mice to generate the Cx43‐cKO mice, with the flox/+ Cre^−^ mice serving as controls. All functional experiments in this study were performed using 2‐month‐old male mice. For the survival analysis, animals were monitored for up to 56 days, and no signs of cage fighting or injury‐related deaths were observed during the entire observation period. For the normal diet group, mice were fed on normal diet and administered NS through oral gavage for a duration of 14 days. For the proline‐supplemented group, mice were fed on normal diet and received daily oral gavage of NS supplemented with 5% proline for 14 days. For the proline‐deprived group, mice were fed on proline‐deprived diet and received daily oral gavage of NS for 14 days. The mice were euthanized with carbon dioxide after the experiments.

### 4.33 In Vivo Electrophysiological Analysis

Mice were anesthetized with 1% isoflurane vapor. Electrode needles were inserted subcutaneously into the left, right upper limb, and right lower limb for ECG recording (iWorx system, IX‐RA‐B3G). ECG waveforms were continuously monitored until a stable baseline and consistent heart rate were established. Baseline ECG was recorded for 3 min, followed by the intraperitoneal injection of ISO (2 mg/kg) and caffeine (120 mg/kg). ECG waveforms were then continuously recorded for another 15 min. ECG traces were analyzed using the LabScribe 4.361 (iWorx system).

For the in vivo electrophysiological analysis, VA was defined according to the Lambeth Conventions II [50]. To minimize confounding factors, PVC was defined as ≥3 premature ventricular complexes within 30 s. VT was defined as ≥5 consecutive premature ventricular complexes in ECG recording. VF was defined as the occurrence on the ECG of coarse and irregular oscillations without discernible QRS complexes or T waves. AVB was defined as PR interval prolongation (first‐degree), intermittent ventricular dropped beats (second degree), or complete dissociation of P‐waves and QRS complexes (third‐degree). To avoid transient variations, AVB was only considered significant if it persisted continuously for at least 10 s. The heart rate was determined by counting the number of complete *R*‐wave complexes over a 60‐s interval after ECG signal has stabilized. No VT or VF episodes were detected under our experimental conditions, and therefore these categories were not plotted in the figures.

### 4.34 Compounds and Solutions

All the chemicals used in the electrophysiological experiments were purchased from Sigma–Aldrich. Fura‐2 AM was purchased from Invitrogen and stock solutions were both prepared in 1 mm in 20% Pluronic F‐127 (Sangon Biotech, A600750) dissolved in ‌dimethyl sulfoxide‌ (DMSO) (Sigma–Aldrich, D2650). NAC was purchased from Sigma–Aldrich (HY‐B0215) and stock solutions were prepared in 500 mm in PBS. MT was purchased from Sigma–Aldrich (HY‐112879) and stock solutions were prepared in 10 mm in DMSO. Proline was purchased from Sigma–Aldrich (P8865) and stock solutions were prepared in 100 mm in PBS.

### 4.35 Statistical Analysis

Normality was examined by the Shapiro–Wilk test (*n* < 10) and the Kolmogorov–Smirnov test (*n* ≥ 10). Homogeneity of variance was examined by the *F*‐test (two groups) or Brown–Forsythe test/Bartlett's test (multiple groups). For data normally distributed and having equal variances, statistical significance was determined by unpaired two‐tailed Student's *t*‐test to compare two groups, and by one‐way or two‐way ANOVA to compare multiple groups. For data normally distributed but having unequal variances, statistical significance was determined by unpaired two‐tailed Welch's *t*‐test to compare two groups, and by Brown–Forsythe ANOVA test/Welch's ANOVA test followed by Dunnett's T3 posthoc test to compare multiple groups. For non‐normally distributed data, statistical significance was determined by Mann–Whitney test to compare two groups, and by Kruskal–Wallis test followed by Dunn's posthoc test to compare multiple groups. A *p* value of <0.05 was considered statistically significant. Data were shown as mean ± SEM (standard error of the mean) and analyzed using GraphPad Prism (GraphPad Software).

## Author Contributions

P.L. and C.J. designed and supervised the study. H.Y., H.F., Y.W., R.J., D.C., H.C., and H.W. performed the experiments and analyzed data. H.Y. and P.L. wrote the manuscript.

## Conflicts of Interest

The authors declare no conflicts of interest.

## Supporting information




**Supporting File 1**: advs74099‐sup‐0001‐SuppMat.pdf.

**Supporting File 2**: advs74099‐sup‐0002‐Data.zip.

## Data Availability

The accession numbers for the RNA‐Seq and Metabolomics data reported in this study are PRJNA1265096 (https://www.ncbi.nlm.nih.gov/) and MTBLS13302 (https://www.ebi.ac.uk/metabolights/), respectively. The data that supports our findings in this study remains available from the corresponding author upon reasonable request.
