# Supplementary material for: Connexin43 Deficiency Leads to Ventricular Arrhythmias by Reprogramming Proline Metabolism
Source: Adv Sci (Weinh). 2026 Jan 31;13(19):e16090. doi: 10.1002/advs.202516090 (PMC13045320; doi:10.1002/advs.202516090)
Supplement: Supplementary file 1 — Supporting File 1: advs74099‐sup‐0001‐SuppMat.pdf. [file ADVS-13-e16090-s002.pdf]

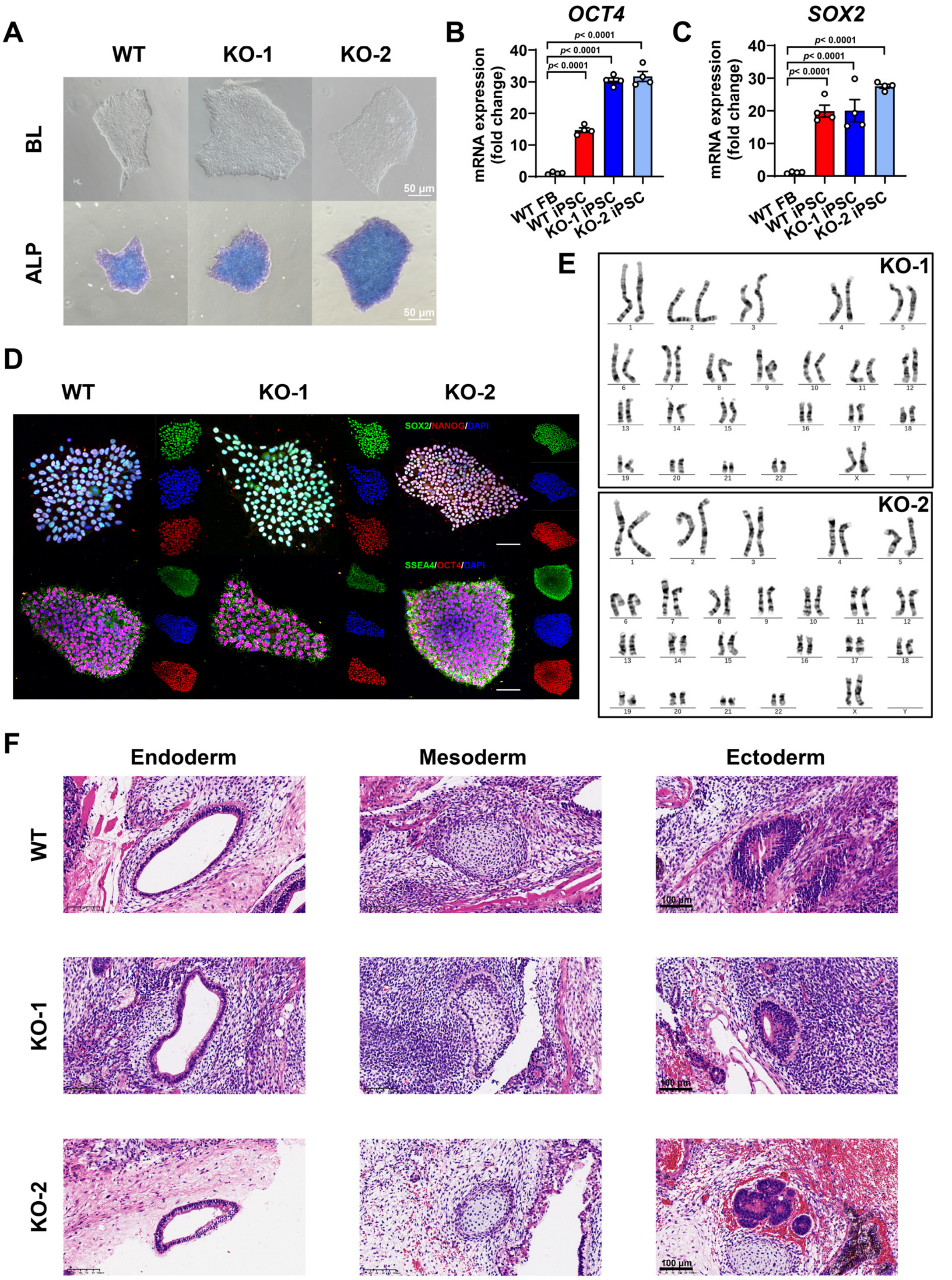

Supplemental Figure 1. Generation and characterization of connexin43 knockout (Cx43-KO) induced pluripotent stem cells (iPSCs) and iPSC-derived cardiomyocytes (iPSC-CMs). A. Representative graphs of bright light (BL) and alkaline phosphatase (ALP) staining of wild-type (WT) and Cx43-KO iPSCs. Scale bar, 50 μm. **B-C.** Bar graphs to compare the mRNA expression of the pluripotency genes *OCT4* and *SOX2*. n= 4 independently biological repeats. **D.** Representative graphs of pluripotent staining of WT and Cx43-KO iPSCs using SOX2 (green), NANOG (red), SSEA-4 (green) and OCT4 (red). DAPI indicates nuclear staining (blue). Scale bar, 10 μm. **E.** Karyotype analysis of Cx43-KO iPSCs. **F.** Teratoma formation assay using WT, KO-1 and KO-2 iPSCs showing derivations of three germ layers. Scale bar, 100 μm.

KO-1 and KO-2 represent two different Cx43-KO iPSC clones. p values were calculated using One-way ANOVA followed by Sidak multiple comparisons test (B), and One-way ANOVA followed by Dunnett's multiple comparisons test (C). Data were shown as mean ± sem.

A

Off-targets		
**Location**	Number of mismatches	Sequence (including mismatches)
**Chr5:109051703**	3	GggCCAgTGGTCGCATGGTAAGG

B

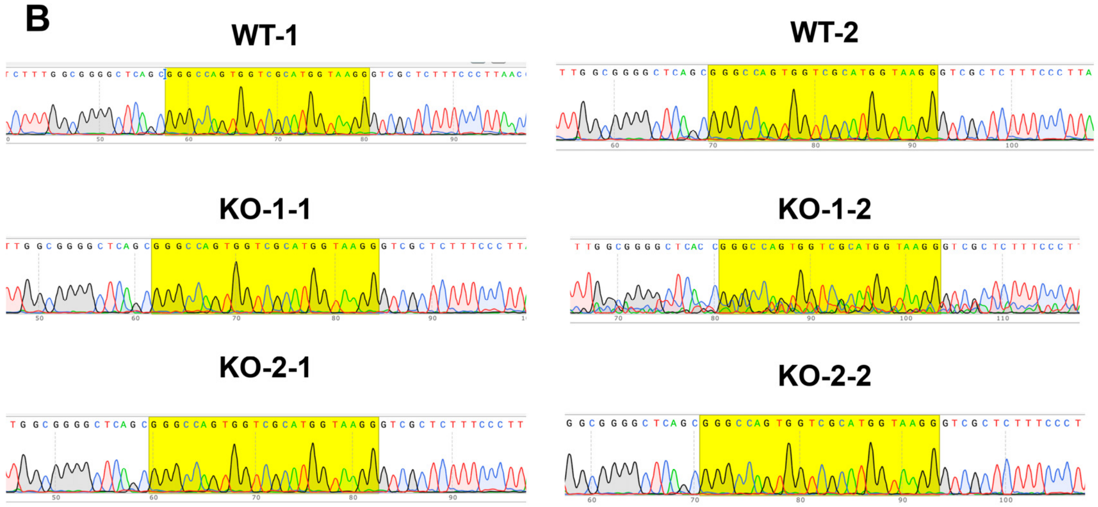

Supplemental Figure 2. No off-target occurrences in Cx43-KO iPSCs. A. The sequences of *GJA1*-guide RNA (gRNA) potential off-target sites. **B.** Sanger sequencing results of WT, KO-1, and KO-2 iPSCs. WT-1 and WT-2 represent two independently sequenced WT iPSC samples; KO-1-1 and KO-1-2 represent two independently sequenced KO-1 iPSC samples; KO-2-1 and KO-2-2 represent two independently sequenced KO-2 iPSC samples. These data indicate the absence of detectable off-target editing at the predicted loci.

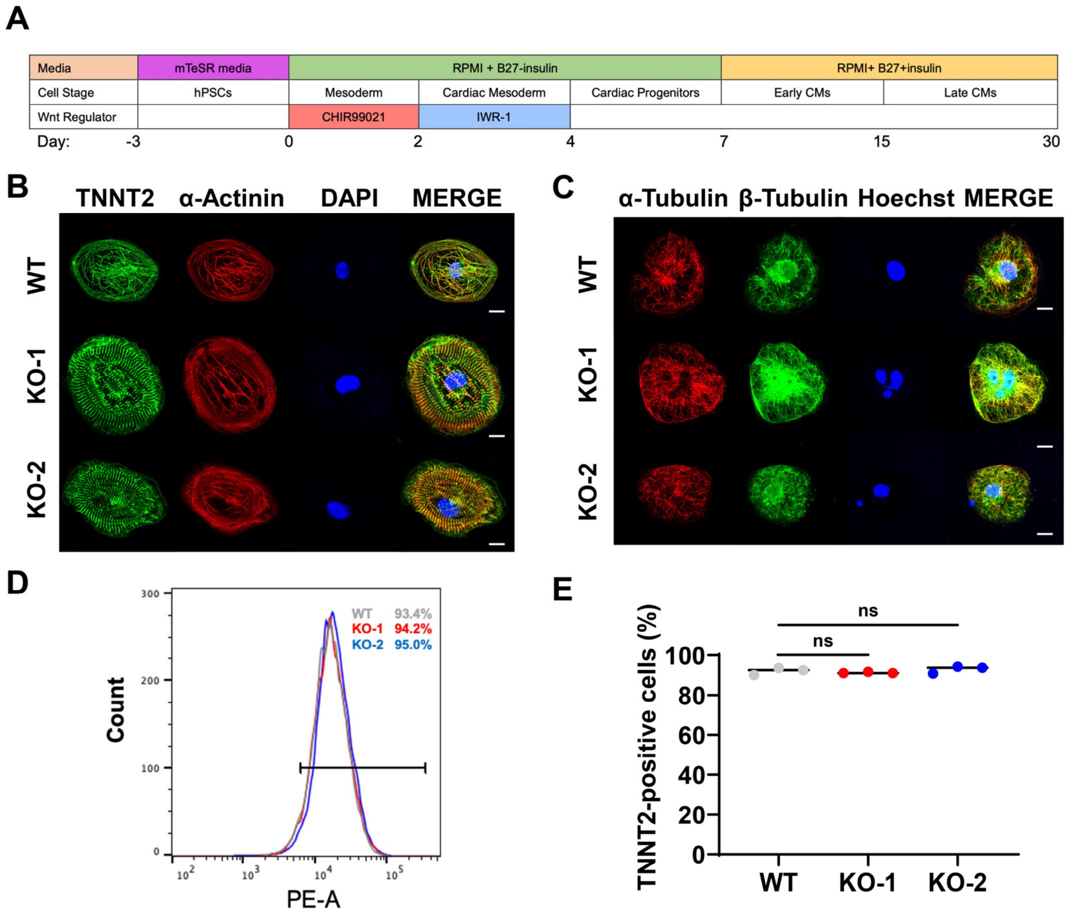

Supplemental Figure 3. Characterization of iPSC-derived cardiomyocytes (iPSC-CMs). A. The schematic diagram showing the two-dimensional (2D) small molecule-based cardiomyocyte differentiation. **B.** Representative graphs of cardiac-specific staining of TNNT2 (green) and α-Actinin (red) in WT and Cx43-KO iPSC-CMs. DAPI indicates nuclear staining (blue). Scale bar, 10 μm. **C.** Representative graphs of microtubule-specific staining of α-tubulin (red) and β-tubulin (green) in WT and Cx43-KO iPSC-CMs. Hoechst indicates nuclear staining (blue). Scale bar, 10 μm. **D-E.** Fluorescence-activated cell sorting (FACS) analysis of the percentage of TNNT2-positive cells in WT and Cx43-KO iPSC-CMs. n= 3 independently biological repeats. *p* values were calculated using One-way ANOVA followed by Dunnett's multiple comparisons test (E). Data were shown as mean ± sem.

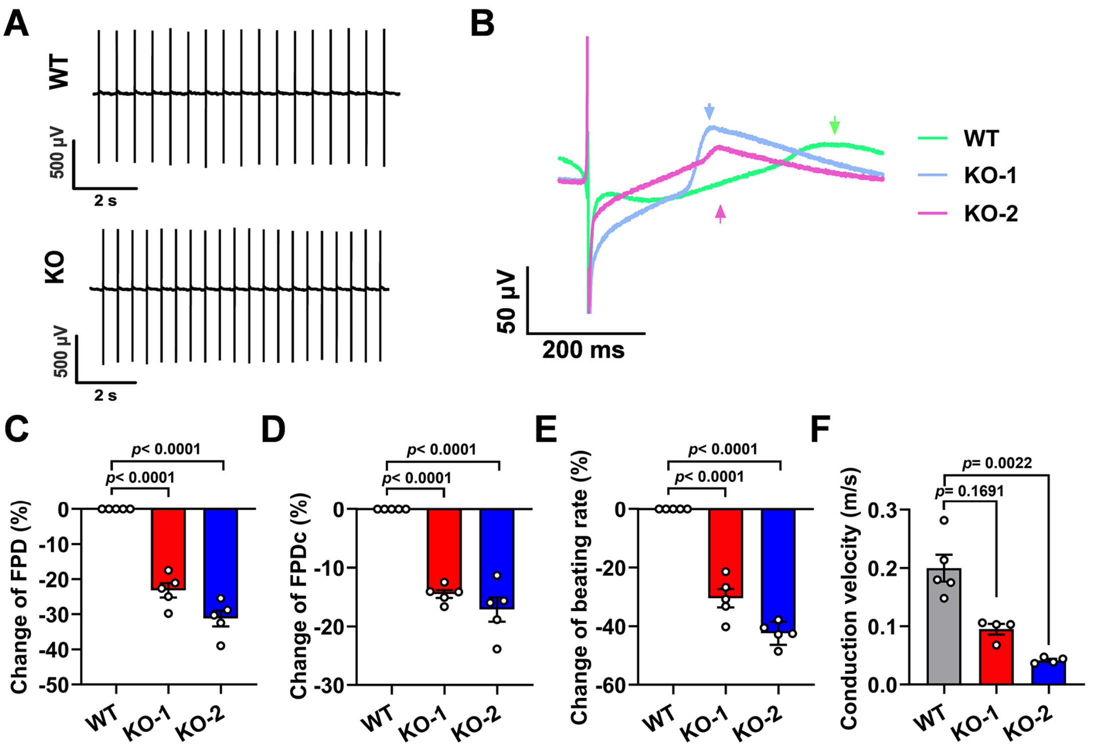

Supplemental Figure 4. Multi-electrode array (MEA) recordings in WT and Cx43-KO iPSC-CMs. A-B. Representative field potential waveforms recorded by MEA from WT and Cx43-KO iPSC-CMs. **C-F.** Bar graphs to compare the change of field potential duration (FPD), change of corrected FPD (FPDc), change of beating rate, and conduction velocity between WT and Cx43-KO iPSC-CMs. n= 4–5 independently biological repeats. p values were calculated using One-way ANOVA followed by Dunnett's multiple comparisons test (C-E) and Kruskal–Wallis test followed by Dunn's multiple comparisons test (F). Data were shown as mean ± sem.

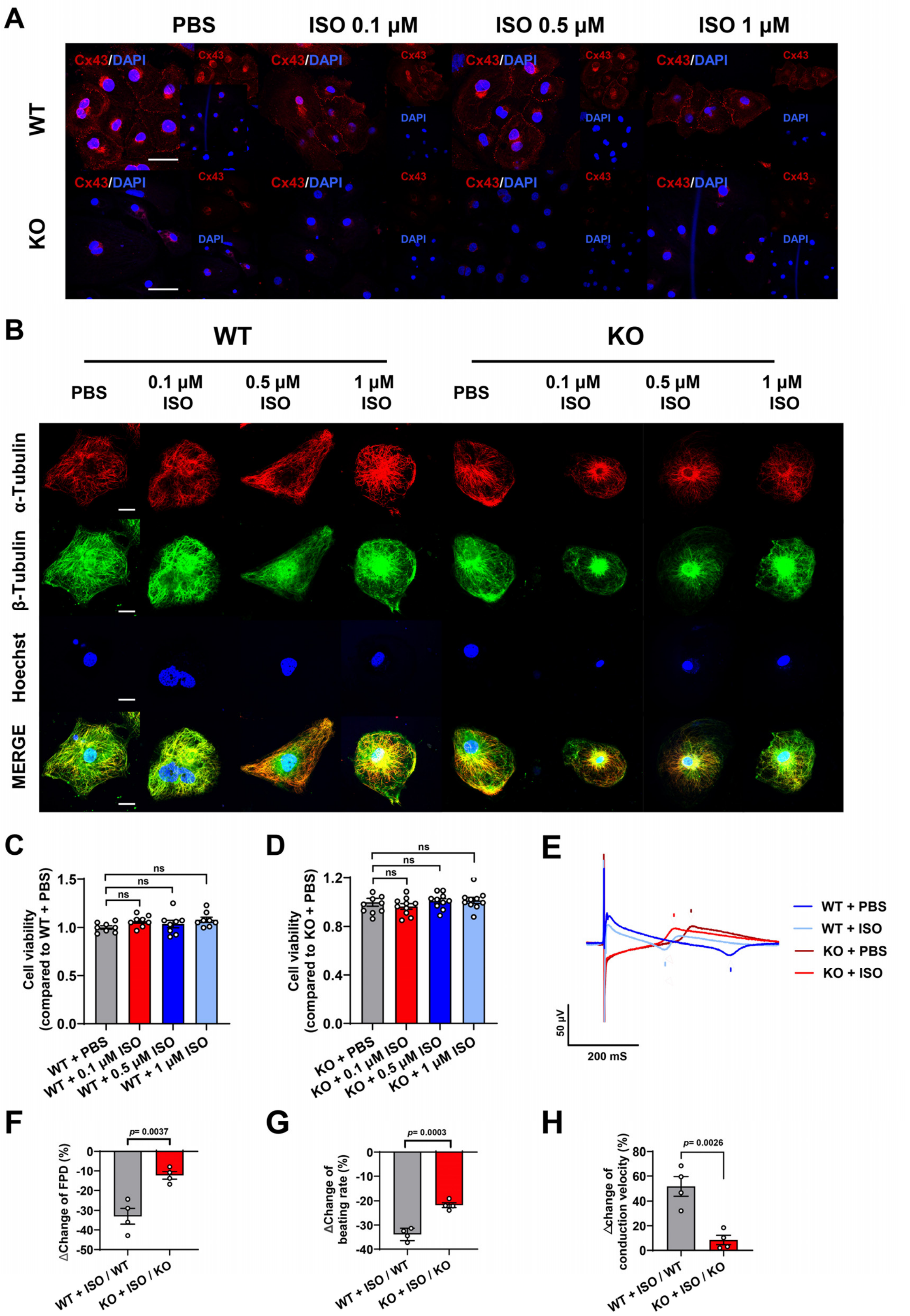

Supplemental Figure 5. Analysis of gap junction formation, microtubule system and cell viability in WT and Cx43-KO iPSC-CMs with or without isoproterenol (ISO) stimulation. A. Representative graphs of staining of Cx43 (red) in WT and Cx43-KO iPSC-CMs with or without ISO (0.1, 0.5 and 1 μM) stimulation. DAPI indicates nuclear staining (blue). Scale bar, 50 μm. **B.** Representative graphs of microtubule-specific staining of α-tubulin (red) and β-tubulin (green) in WT and Cx43-

KO iPSC-CMs with or without ISO (0.1, 0.5 and 1 μM) stimulation. DAPI indicates nuclear staining (blue). Scale bar, 10 μm. **C-D.** Bar graphs to compare the cell viability in WT and Cx43-KO iPSC-CMs with or without ISO (0.1, 0.5 and 1 μM) stimulation. n= 10 independently biological repeats. **E.** Representative field potential waveforms recorded by MEA from WT and Cx43-KO iPSC-CMs with or without ISO (0.1 μM) stimulation. **F-H.** Bar graphs to compare the △change of FPD, △change of FPDc, and △change of conduction velocity between WT and Cx43-KO iPSC-CMs with or without ISO stimulation. n= 4 independently biological repeats. "KO" in the figure panels refers to combined data from KO-1 and KO-2 analyzed in parallel (A-H). *p* values were calculated using One-way ANOVA followed by Sidak multiple comparisons test (C), One-way ANOVA followed by Bonferroni's multiple comparisons test (D), and unpaired two-tailed Student's t-test (F-H). Data were shown as mean ± sem.

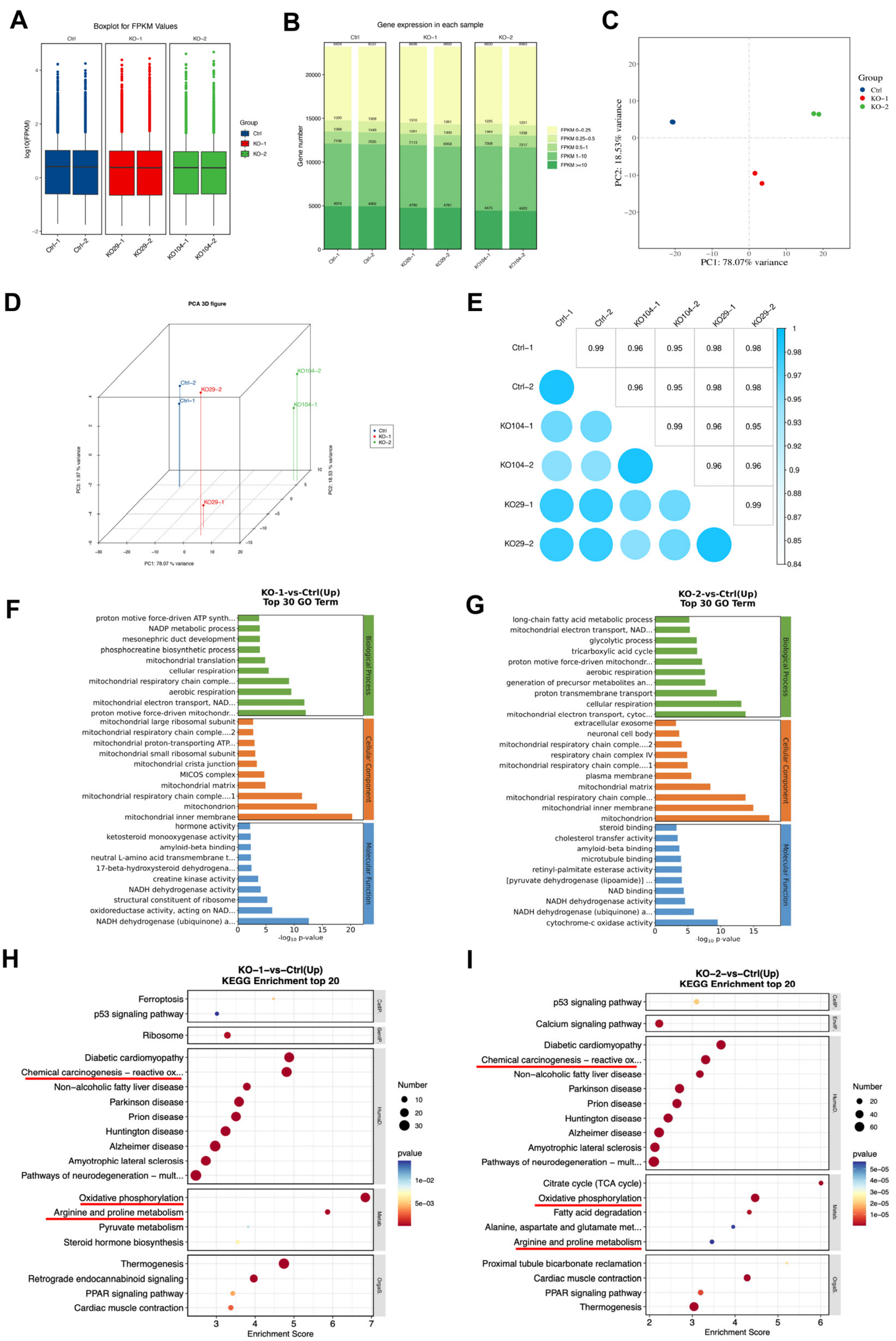

Supplemental Figure 6. Transcriptomic analysis of Cx43-KO iPSC-CMs. A-B. Bar graph and density distribution plots to compare the gene expression between WT and Cx43-KO iPSC-CMs. **C-D.** The principal component analysis (PCA) scatter plot showing showed significant differences in the variation among the groups. **E.** Clustering analysis of WT and Cx43 KO iPSC-CMs. **F-G.** Top 30 Gene ontology (GO) enrichment analysis of the upregulated differentially expressed genes (DEGs) between WT and Cx43-KO iPSC-CMs. The DEGs were positively enriched in "NADH

dehydrogenase activity", "Mitochondrial respiratory chain complex I/IV" and "Proton motive force- driven mitochondrial ATP synthesis". **H-I.** Top 20 Kyoto encyclopedia of genes and genomes (KEGG) enrichment analysis between WT and Cx43-KO iPSC-CMs. The DEGs were positively enriched in "Arginine and proline metabolism", "Chemical carcinogenesis reactive oxygen species" and "Oxidative phosphorylation (OXPHOS)" pathways. Ctr-1 and Ctr-2 represent two independent differentiations of WT iPSCs; KO29-1 and KO29-2 represent two independent differentiations of KO-1 iPSCs; K104-1 and K104-2 represent two independent differentiations of KO-2 iPSCs. No technical replicates were used in the omics datasets.

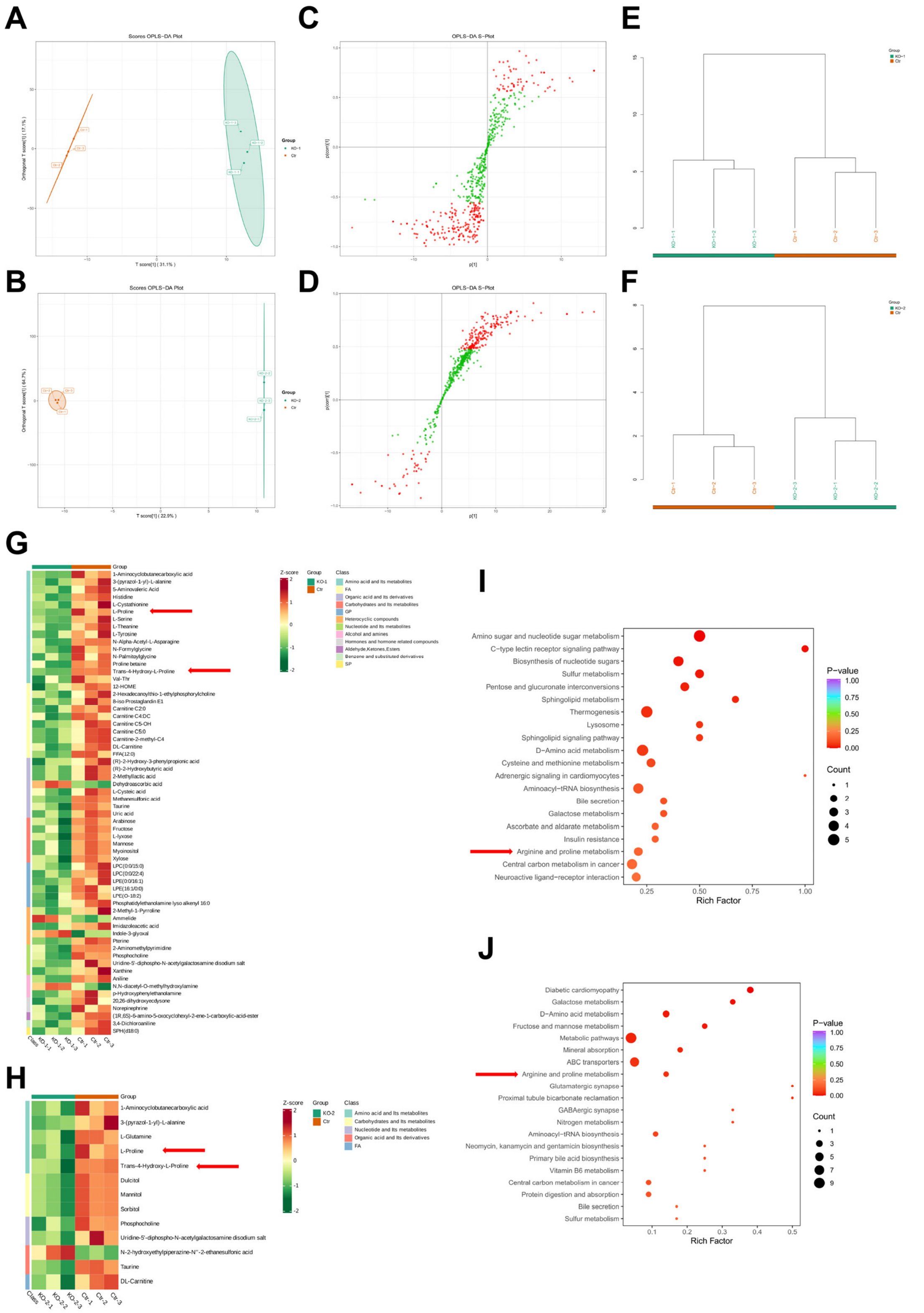

Supplemental Figure 7. Metabolomics analysis of Cx43-KO iPSC-CMs. A-B. Orthogonal partial least squares-discriminant analysis (OPLS-DA) of metabolome data for the comparisons between WT and KO-1 iPSC-CMs, as well as between WT and KO-2 iPSC-CMs. **C-D.** S-plots from OPLS-DA model between WT and KO-1 iPSC-CMs, as well as between WT and KO-2 iPSC-CMs. **E-F.** Hierarchical clustering dendrograms to compare the differences between WT and KO-1 iPSC-CMs, as well as between WT and KO-2 iPSC-CMs. **G-H.** Cluster heatmaps of differentially expressing

metabolites (DEMs) between WT and KO-1 iPSC-CMs, as well as between WT and KO-2 iPSC-CMs. Red arrows indicate “L-Proline” and “Trans-4-Hydroxy-L-Proline” involved in proline metabolism that were decreased in Cx43-KO iPSC-CMs. **I-J.** Human metabolome database (HMDB) enrichment analysis of DEMs between WT and KO-1 iPSC-CMs, as well as between WT and KO-2 iPSC-CMs. Red arrows indicate “Arginine and proline metabolism”. Ctr-1, Ctr-2 and Ctr-3 represent three independent differentiations of WT iPSCs; KO-1-1, KO-1-2 and KO-1-3 represent three independent differentiations of KO-1 iPSCs; KO-2-1, KO-2-2 and KO-2-3 represent three independent differentiations of KO-2 iPSCs. No technical replicates were used in the omics datasets.

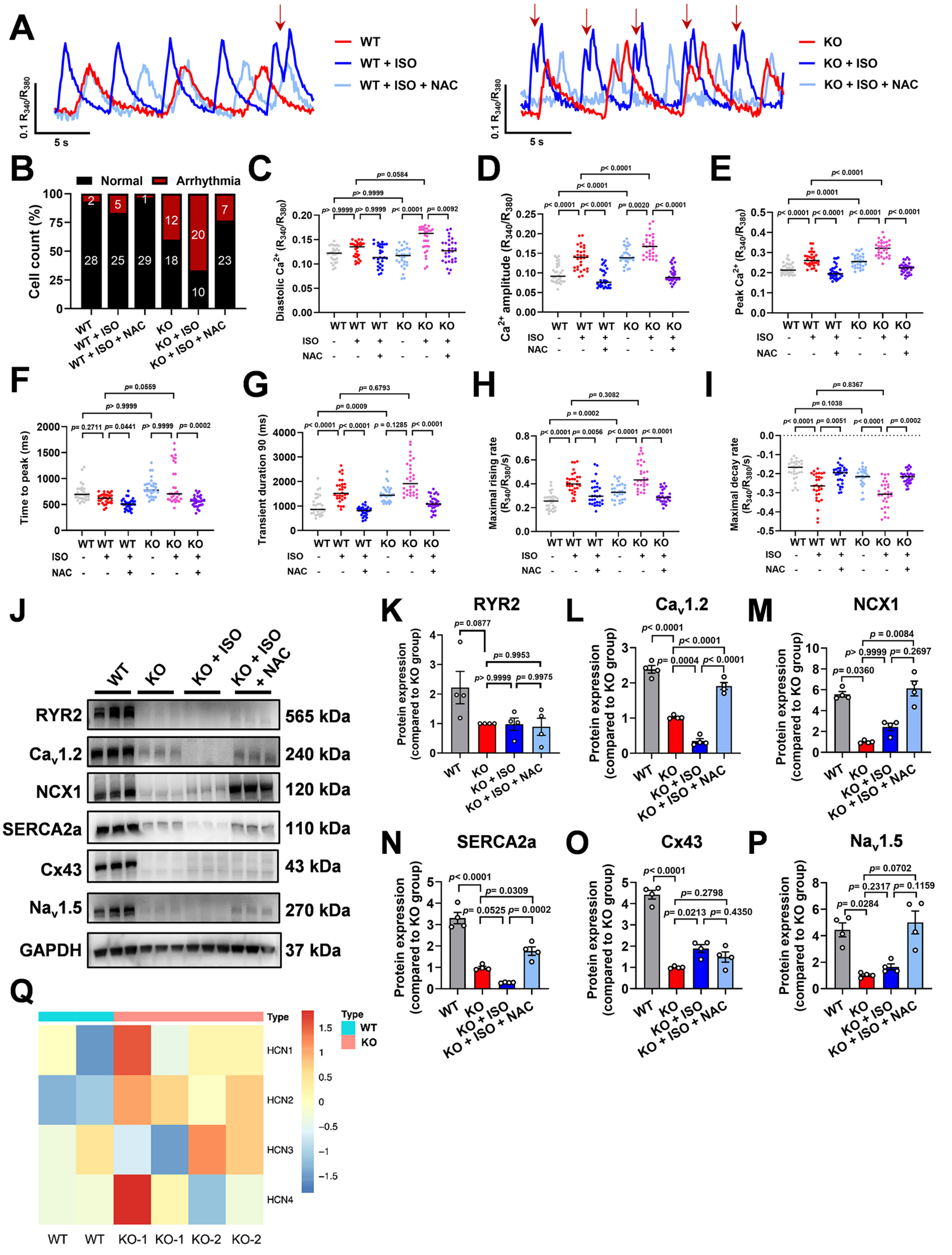

Supplemental Figure 8. NAC (N-acetyl-L-cysteine) treatment alleviates calcium handling abnormalities in Cx43-KO iPSC-CMs. A. Representative calcium (Ca^{2+}) transient waveforms recorded by ratiometric Fura-2 imaging from WT and Cx43-KO iPSC-CMs with or without ISO (100 nM, 2 hours) stimulation, and with or without treatment of NAC (5 mM, 4 hours). Red arrows indicate arrhythmia-like transients. **B.** Bar graph to compare the percentage of cells with arrhythmia in A. n= 30 cells. **C-I.** Scatter dot plots to compare diastolic calcium, calcium amplitude, peak calcium, time to peak, transient duration 90, maximal decay rate, and maximal rising rate among different groups in A. n= 30 cells. **J-P.** Western blot analysis of the protein expression of ryanodine receptor 2 (RYR2), L-type calcium channel $Ca_v1.2$, sodium-calcium

exchanger 1 (NCX1), sarco/endoplasmic reticulum calcium ATPase 2a (SERCA2a), Cx43, and cardiac sodium channel $Na_v1.5$ in WT and Cx43-KO iPSC-CMs with or without ISO (100 nM, 2 hours) stimulation, and with or without treatment of NAC (5 mM, 4 hours). GAPDH is used as the loading control. n= 4 independently biological repeats. **Q.** Heatmap demonstrating the gene expression of pacemaker channels (*HCN1*, *HCN2*, *HCN3* and *HCN4*) between WT and Cx43-KO iPSC-CMs. “KO” in the figure panels refers to combined data from KO-1 and KO-2 analyzed in parallel (A-Q). *p* values were calculated using Kruskal–Wallis test followed by Dunn’s multiple comparisons test (C, F-G, I, M), One-way ANOVA followed by Tukey’s multiple comparisons test (D-E, K-L, N-O), and Brown–Forsythe ANOVA test/Welch ANOVA test followed by Dunnett T3 multiple comparisons test (H, P). Data were shown as mean ± sem.

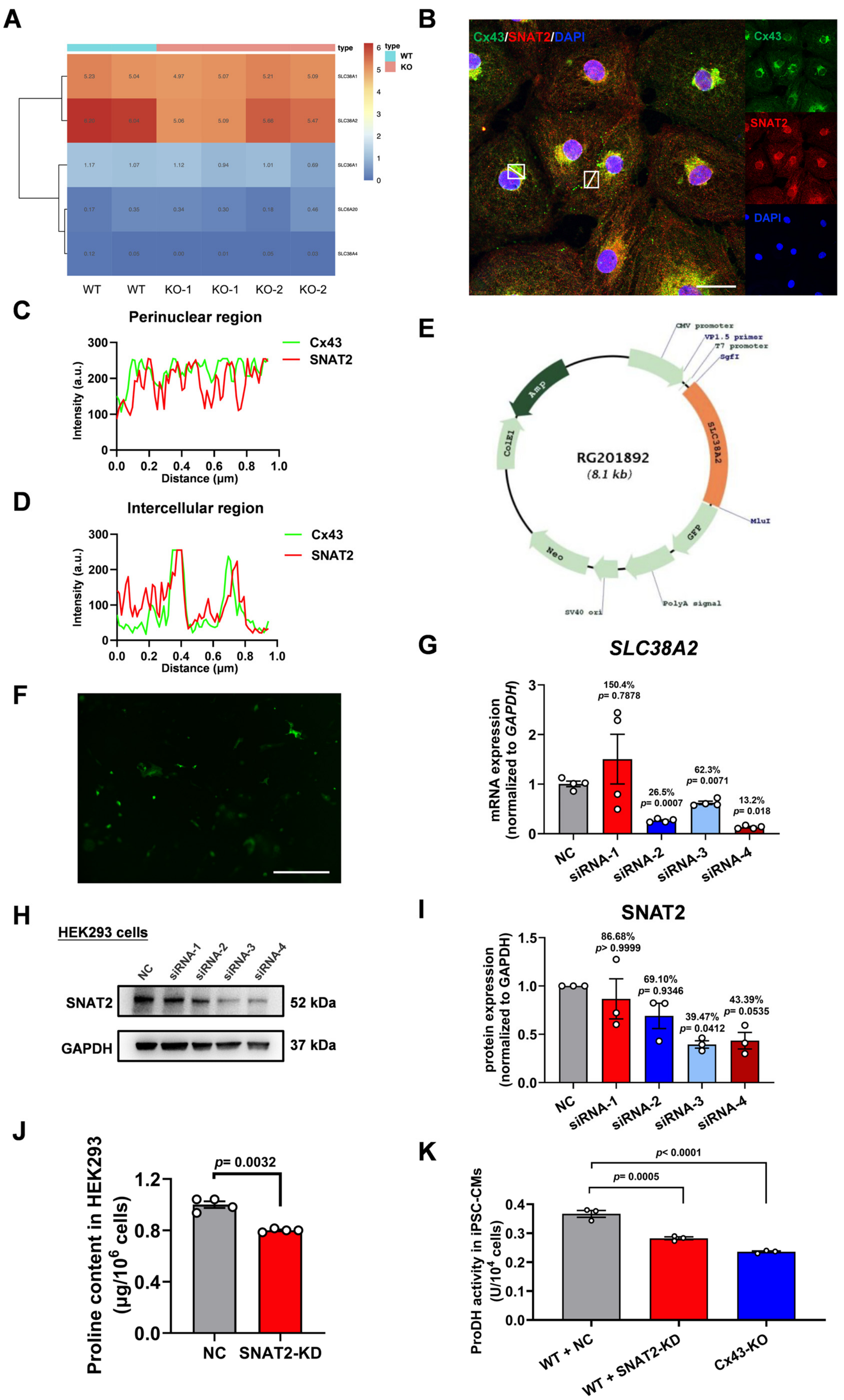

A
type
WT
KO
SLC38A1
SLC38A2
SLC36A1
SLC6A20
SLC38A4
WT
WT
KO-1
KO-1
KO-2
KO-2
B
Cx43/SNAT2/DAPI
Cx43
SNAT2
DAPI
C
Perinuclear region
Cx43
SNAT2
Intensity (a.u.)
Distance (μm)
D
Intercellular region
Cx43
SNAT2
Intensity (a.u.)
Distance (μm)
E
RG201892
(8.1 kb)
CMV promoter
VP1.5 primer
T7 promoter
SgfI
SLC38A2
MluI
GFP
PolyA signal
SV40 ori
Neo
ColE1
Amp
F
G
SLC38A2
mRNA expression (normalized to GAPDH)
150.4% p= 0.7878
26.5% p= 0.0007
62.3% p= 0.0071
13.2% p= 0.018
NC
siRNA-1
siRNA-2
siRNA-3
siRNA-4
H
HEK293 cells
NC
siRNA-1
siRNA-2
siRNA-3
siRNA-4
SNAT2
52 kDa
GAPDH
37 kDa
I
SNAT2
protein expression (normalized to GAPDH)
86.68% p> 0.9999
69.10% p= 0.9346
39.47% p= 0.0412
43.39% p= 0.0535
NC
siRNA-1
siRNA-2
siRNA-3
siRNA-4
J
Proline content in HEK293 (μg/10^6 cells)
p= 0.0032
NC
SNAT2-KD
K
ProDH activity in iPSC-CMs (U/10^4 cells)
p< 0.0001
p= 0.0005
WT + NC
WT + SNAT2-KD
Cx43-KO

Supplemental Figure 9. Genetic manipulation of sodium-dependent neutral amino acid transporter (SNAT2) in human embryonic kidney 293 (HEK293) cells and iPSC-CMs. A. Heatmap demonstrating the gene expression of proline transporters between WT and Cx43-KO iPSC-CMs. *SLC38A1* and *SLC38A2* had a high abundance in iPSC-CMs. The unit of the expression values in the heatmap is $\log_2$ (FPKM). n= 2 independently biological replicates for WT, KO-1 and KO-2. **B.** Representative graphs of staining of Cx43 (green) and SNAT2 (red) in WT iPSC-CMs. DAPI indicates nuclear staining (blue). Scale bar, 50 μm. **C-D.** Co-localization analysis of Cx43 and SNAT2 in B. **E.** Schematic diagram of the SNAT2 plasmid. **F.** The immunofluorescent image to verify the transfection efficiency in WT iPSC-CMs. The Green fluorescence indicates iPSC-CMs successfully transfected with SNAT2. Scale bar, 100 μm. **G.** Bar graph to compare the mRNA expression of *SLC38A2* among HEK293 (human embryonic kidney 293) cells transfected with scrambled small interfering RNA (siRNA) (negative control, NC) and four different SNAT2 siRNAs. n= 4 independent experiments. **H-I.** Western blot analysis of the SNAT2 protein expression in HEK293 cells transfected with scrambled siRNA (NC) and four different SNAT2 siRNAs. GAPDH is used as the loading control. n= 3 independently biological repeats. **J.** Bar graph to compare the proline content measured using a proline assay kit between HEK293 cells transfected with scrambled siRNA (NC) and SNAT2 siRNA-4 (SNAT2 knockdown, SNAT2-KD). n= 4 independently biological repeats. **K.** Bar graph to compare the ProDH (proline dehydrogenase) activity among WT iPSC-CMs transfected with scrambled siRNA (NC), WT iPSC-CMs transfected with SNAT2 siRNA-4 (SNAT2-KD), and Cx43-KO (KO-1) iPSC-CMs. n= 3 independently biological repeats. *p* values were calculated using Brown–Forsythe ANOVA test/Welch ANOVA test followed by Dunnett T3 multiple comparisons test (G), Kruskal–Wallis test followed by Dunn's multiple comparisons test (I), unpaired t test with Welch's correction (J), and One-way ANOVA followed by Tukey's multiple comparisons test (K). Data were shown as mean ± sem.

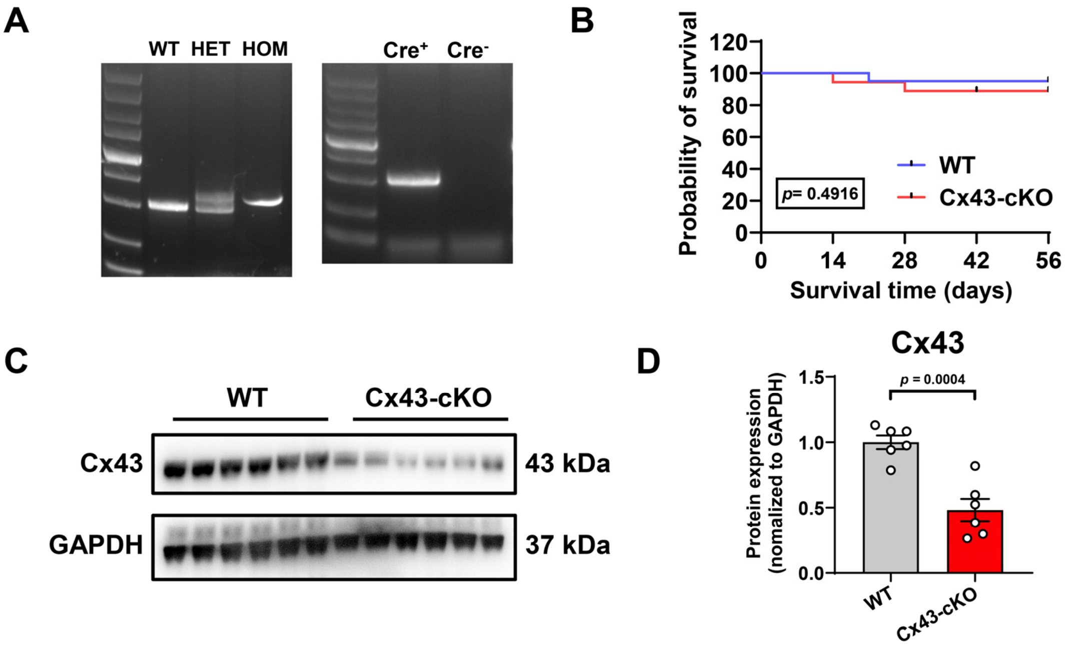

Supplemental Figure 10. The Cx43-interacting protein SNAT2 is downregulated in heterozygous cardiac-specific conditional Cx43-KO (Cx43-cKO) mouse ventricular tissues. A. Identification of WT, heterozygous (HET) and homozygous (HOM) Cx43-cKO mice. **B.** Survival curves of WT and HET Cx43-cKO mice during a 56-day observation window. n= 20 mice. **C-D.** Western blot analysis of the Cx43 protein expression in ventricular tissues collected from 2-month-old WT and HET Cx43-cKO mice. GAPDH is used as the loading control. n= 6 mice. *p* values were calculated using unpaired two-tailed Student's t-test (D). Data were shown as mean ± sem.

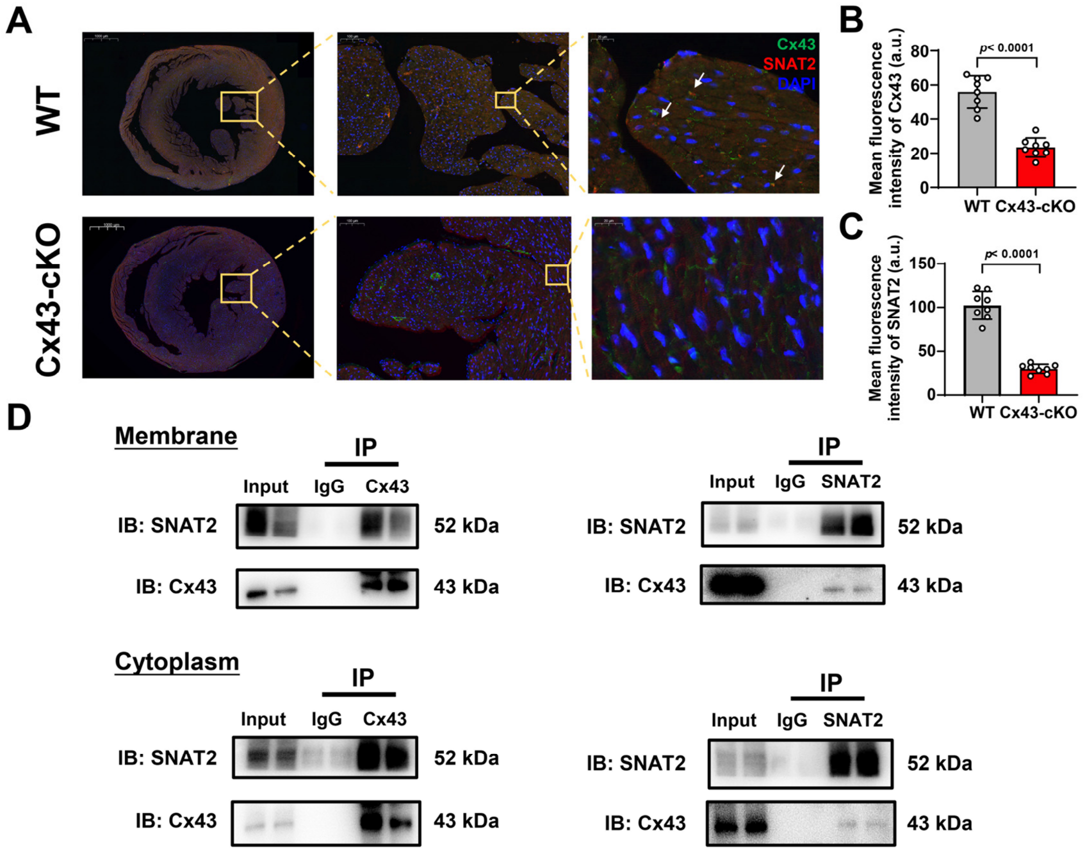

Supplemental Figure 11. SNAT2 interacts with Cx43 and exhibits reduced expression in mouse ventricular tissues with Cx43 deficiency. A. Representative graphs of Cx43 staining (green) and SNAT2 (red) in ventricular tissues collected from 2-month-old WT and HET Cx43-cKO mice. DAPI indicates nuclear staining (blue). Scale bar (left panel), 1000 µm. Scale bar (middle panel), 100 µm. Scale bar (right panel), 20 µm. White arrows indicate sites of co-localization. **B.** Bar graph to compare the mean fluorescence intensity of Cx43 between 2-month-old WT and HET Cx43-cKO mouse ventricular tissues. n= 8 views in 3 mice. **C.** Bar graph to compare the mean fluorescence intensity of SNAT2 between 2-month-old WT and HET Cx43-cKO mouse ventricular tissues. n= 8 views in 3 mice. **D.** Co-immunoprecipitation (co-IP) assays to detect interaction between Cx43 and SNAT2 on the cell membrane and in the cytoplasm of the 2-month-old WT mouse ventricular tissues using SNAT2 antibody, Cx43 antibody or IgG (negative control antibody). p values were calculated using unpaired two-tailed Student's t-test (B-C). Data were shown as mean ± sem.

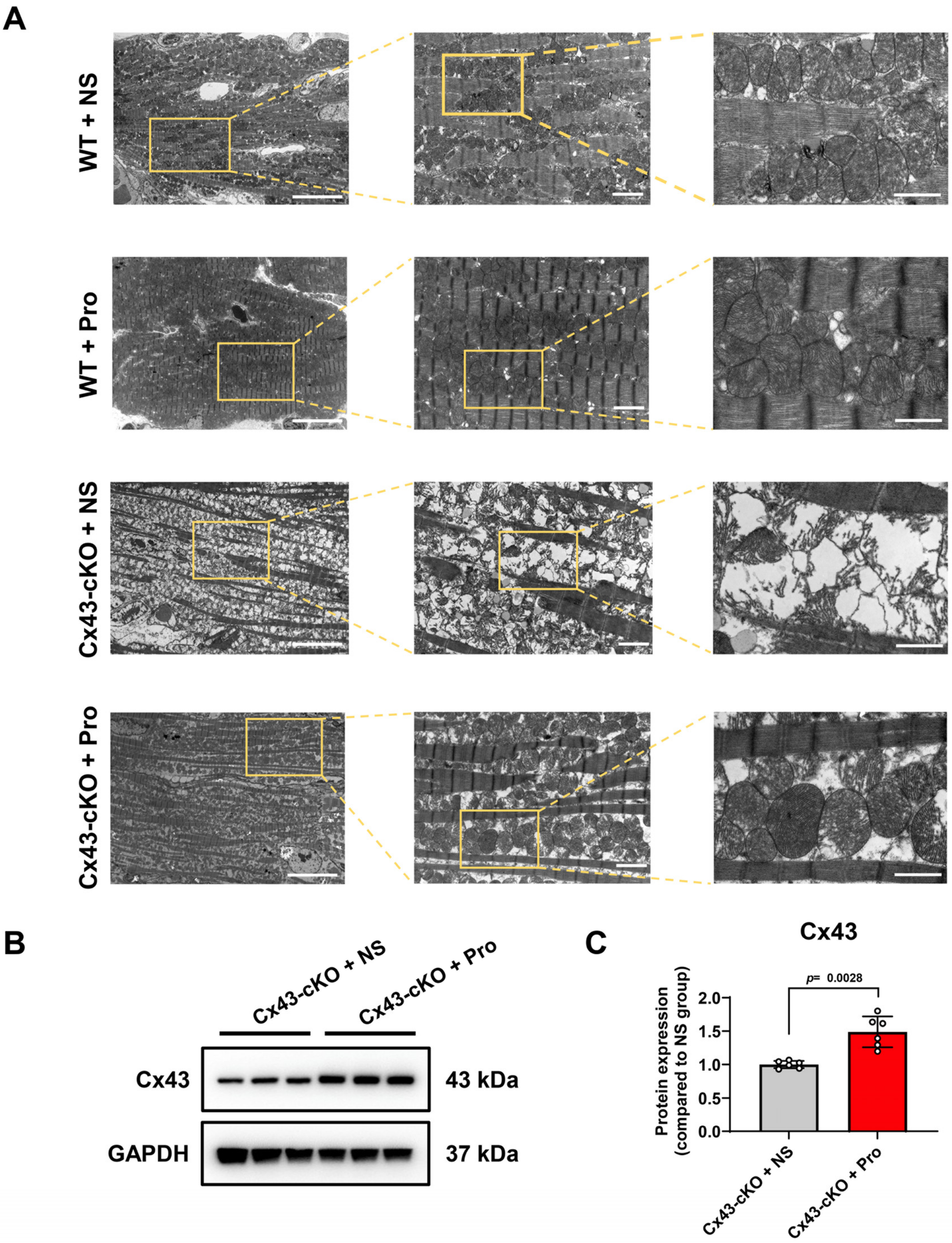

Supplemental Figure 12. Proline supplementation restores Cx43 expression and improves the mitochondrial ultrastructure in the HET Cx43-cKO mouse ventricular tissues. A. Representative transmission electron microscopy images of mitochondrial ultrastructure in ventricular tissues from 2-month-old WT and HET Cx43-cKO mice fed on normal or proline-supplemented diet. Scale bar (left panel), 200 µm. Scale bars (middle and right panels), 10 µm. "NS" denotes normal saline (NS), and "Pro" denotes proline. **B-C.** Western blot analysis of the Cx43 protein expression in ventricular tissues collected from 2-month-old HET Cx43-cKO mice fed on normal or proline-supplemented diet. GAPDH is used as the loading control. n= 6 mice. *p* value was calculated using unpaired two-tailed Student's t-test (C). Data were shown as mean ± sem.

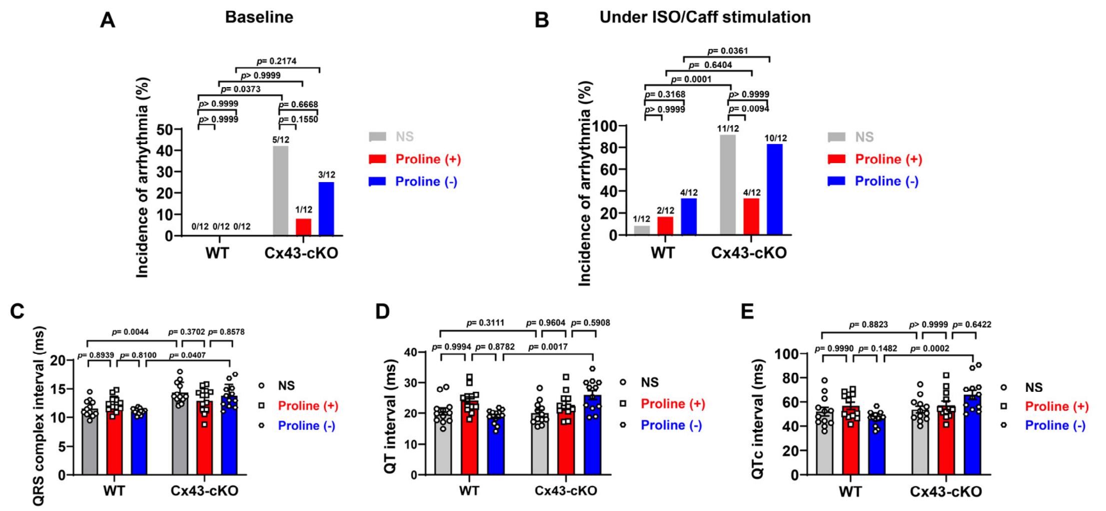

Supplemental Figure 13. Comparison of the incidence of arrhythmia and the key parameters of baseline electrocardiograms (ECGs) between WT and HET Cx43-cKO mice. A-B. Bar graphs to compare the incidence of arrhythmia among 2-month-old WT and HET Cx43-cKO mice fed on normal, proline-supplemented, or proline-deprived diet. **C-E.** Bar graphs to compare the intervals of QRS complex, QT, and corrected QT (QTc) among 2-month-old WT and HET Cx43-cKO mice fed on normal, proline-supplemented, or proline-deprived diet. n= 12 mice. *p* values were calculated using Fisher's exact tests (A-B) and Two-way ANOVA followed by Tukey's multiple comparisons test (C-E). Data were shown as mean ± sem.

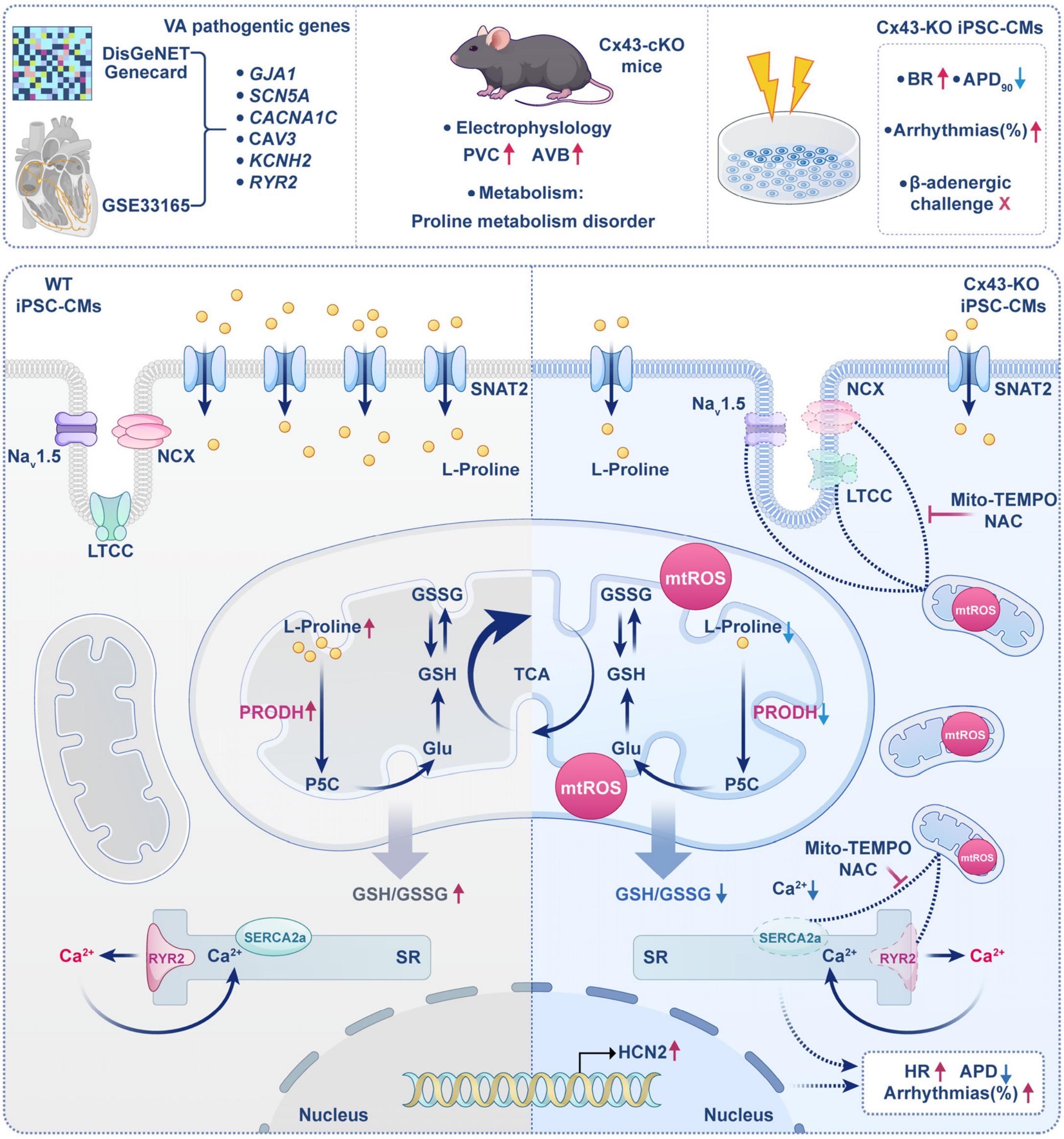

Supplemental Figure 14. Proposed work model. Through deep mining of the public databases, the *GJA1* gene, encoding the predominant ventricular gap junction protein Cx43, is identified as a crucial gene for ventricular arrhythmias (VAs). Utilizing human iPSC-CM and genetic mice as model systems, it has been demonstrated that Cx43 deficiency leads to arrhythmic phenotypes, along with reduced levels of proline. Mechanistically, Cx43 interacts with the amino acid transporter SNAT2 where Cx43 deficiency downregulates SNAT2 expression causing reduced proline transport into cardiomyocytes and resulting in proline metabolic dysfunction. This ultimately leads to disrupted mitochondrial dynamics and oxidative stress. On one hand, disrupted proline metabolism fails to provide additional energy supply for the accelerated heart rate and impairs oxidative stress tolerance, leading to impaired mitochondrial dynamics and function. On the other hand, excessive oxidative stress causes calcium homeostasis imbalance, ultimately leading to arrhythmogenesis. PVC, premature ventricular contraction; AVB, atrioventricular block; BR, beating rate; APD, action potential duration; mtROS, mitochondrial reactive oxygen species; SR, sarcoplasmic reticulum; LTCC, L-type calcium channel; HR, heart rate.

Supplemental Table 1. The list of genes included in the six algorithms.

Rank	Stress	EPC	Degree	MNC	Radiality	Betweenness
1	*ALB*	*SCN5A*	*ALB*	*ALB*	*ALB*	*ALB*
2	*INS*	*ALB*	*INS*	*INS*	*INS*	*INS*
3	*CTNNB1*	*TNNT2*	*SCN5A*	*SCN5A*	*SCN5A*	*CTNNB1*
4	*MYH6*	*MYH6*	*IL6*	*IL6*	*MYH6*	*SCN5A*
5	*TNF*	*RYR2*	*TNF*	*TNF*	*IL6*	*MYH6*
6	*SCN5A*	*INS*	*RYR2*	*RYR2*	*TNF*	*IL6*
7	*IL6*	*CACNA1C*	*MYH6*	*MYH6*	*RYR2*	*TNF*
8	*TNNI3*	*CASQ2*	*TNNT2*	*TNNT2*	*TNNT2*	*RYR2*
9	*GJA1*	*TTN*	*CACNA1C*	*CACNA1C*	*CTNNB1*	*TNNI3*
10	*TNNT2*	*CAV3*	*CTNNB1*	*CTNNB1*	*TNNI3*	*CAV3*
11	*RYR2*	*GJA1*	*CAV3*	*CAV3*	*ACTC1*	*GJA1*
12	*CAV3*	*IL6*	*IL1B*	*IL1B*	*KCNH2*	*LMNA*
13	*CACNA1C*	*TNF*	*TNNI3*	*TNNI3*	*CACNA1C*	*TNNT2*
14	*ACTC1*	*TNNI3*	*KCNH2*	*KCNH2*	*GJA1*	*ACTC1*
15	*IL1B*	*KCNH2*	*TTN*	*TTN*	*IL1B*	*CACNA1C*
16	*LMNA*	*ACTN2*	*ACTC1*	*ACTC1*	*TTN*	*KCNH2*
17	*KCNH2*	*ACTC1*	*CASQ2*	*CASQ2*	*NOS3*	*MT-ND1*
18	*NOS3*	*MYH7*	*GJA1*	*MYH7*	*EDN1*	*PRKAG2*
19	*NPPA*	*CTNNB1*	*MYH7*	*ACTN2*	*NPPA*	*IL1B*
20	*CALM1*	*ACTA2*	*ACTN2*	*GJA1*	*CAV3*	*HRAS*

The *GJA1* gene is highlighted in red and underlined.

Supplemental Table 2. Clinical characteristics of the failing hearts.

Number	Gender	Age	Devices	LVEDD (cm)	EF (%)	Arrhythmia history	Sample
**Ischemic cardiomyopathy**							
1	Female	65	ICD	6.4	15	NSVT, VF	LV, LA
2	Female	49	ICD	5.9	25-30	VT	LV, LA
3	Female	53	ICD	6.5	20	No arrhythmias	LV, LA
4	Female	67	ICD	7	10	No arrhythmias	LV, LA
5	Male	50	PPM, ICD, BiV	7	< 15	Refractory VT	LV, LA
6	Male	44	ICD, LVAD	7	< 15	No arrhythmias	LV, LA
7	Male	69	PPM, ICD, BiV	7.13	39	VT, AF	LV, LA
8	Male	63	PPM, ICD, BiV	7.6	15	No arrhythmias	LV, LA
9	Male	50	ICD	6.6	30	NSVT, VF	LV, LA
10	Male	67	ICD, LVAD	7.9	10	No arrhythmias	LV, LA
**Nonischemic cardiomyopathy**							
11	Female	54	ICD	6.1	< 15	VT, AF	LV (Epicardium, Endocardium)
12	Male	47	LVAD	6.4	25	VT	LV (Epicardium, Endocardium)
13	Male	53	ICD	8.2	16	VT	LV (Epicardium, Endocardium)

ICD: implantable cardioverter-defibrillator; PPM: permanent pacemaker; BiV: biventricular pacing device; LVAD: left ventricular assist device; LVEDD: left ventricular end diastolic diameter; EF: ejection fraction; NSVT: non-sustained ventricular tachycardia; VF: ventricular fibrillation; VT: ventricular tachycardia; AF: atrial fibrillation; LV: left ventricle; LA: left atria.

Supplemental Table 3. Comparison of key action potential parameters between WT and Cx43-KO iPSC-CMs.

	MDP (mV)	APA (mV)	Overshoot (mV)	APD_{50} (ms)	V_{max} (V/s)
WT (n= 17)	-52.9 ± 1.1	100.2 ± 1.5	47.1 ± 1.2	248.5 ± 13.4	9.2 ± 0.5
KO-1 (n= 17)	-57.0 ± 0.7 (p= 0.0017)	100.8 ±1.4 (p= 0.9508)	43.8 ± 1.0 (p= 0.0281)	177.3 ± 13.1 (p= 0.0007)	11.5 ± 1.0 (p= 0.1298)
KO-2 (n= 16)	-56.2 ± 0.7 (p= 0.0133)	100.0 ± 1.4 (p= 0.9895)	43.8 ± 1.4 (p= 0.0322)	181.7 ± 13.0 (p= 0.0017)	11.2 ± 1.1 (p= 0.2161)

Supplemental Table 4. Molecular docking analysis displays the interaction between Cx43 and SNAT2.

Receptor	Ligand	Hydrogen bond Interaction
GJA1 (7XQF)	SLC38A2 (Swiss model)	VAL24-SER280<br>SER27-SER280<br>VAL28-SER280<br>GLY38-THR433<br>PHE70-LYS186

Supplemental Table 5. Primer sequences for qPCR.

Gene	Forward	Reverse
SOX2 (human)	GGGAAATGGGAGGGGTGCAAAAGAGG	TTGCGTGAGTGTGGATGGGATTGGTG
OCT4 (human)	GACAGGGGGAGGGGAGGAGCTAGG	CTTCCCTCCAACCAGTTGCCCCAAAC
GAPDH (human)	GGTCGGAGTCAACGGATTTG	CGGTGCCATGGAATTTGCC
SLC36A1 (human)	ACCTACGCACTCCAGTTCTAC	GGTCCACCACTAACTCACAGT
SLC36A2 (human)	GGAGCCGTTGCCATCAAATTG	CCATGTTGCCTTTCACCAGGT
SLC38A1 (human)	CACCACAGGGAAGTTCGTAATC	CATCCACGTACCAGGCTGAAA
SLC38A2 (human)	ATGAGTTGCCTTTGGTGATCC	ACAGGACACGGAACCTGAAAT
