# Supplementary material for: Connexin43 Deficiency Leads to Ventricular Arrhythmias by Reprogramming Proline Metabolism
Source: Adv Sci (Weinh). 2026 Jan 31;13(19):e16090. doi: 10.1002/advs.202516090 (PMC13045320; doi:10.1002/advs.202516090)
Supplement: Supplementary file 2 — Supporting File 2: advs74099‐sup‐0002‐Data.zip. [file ADVS-13-e16090-s001.zip › Unedited blot and gel images_Final.pdf]

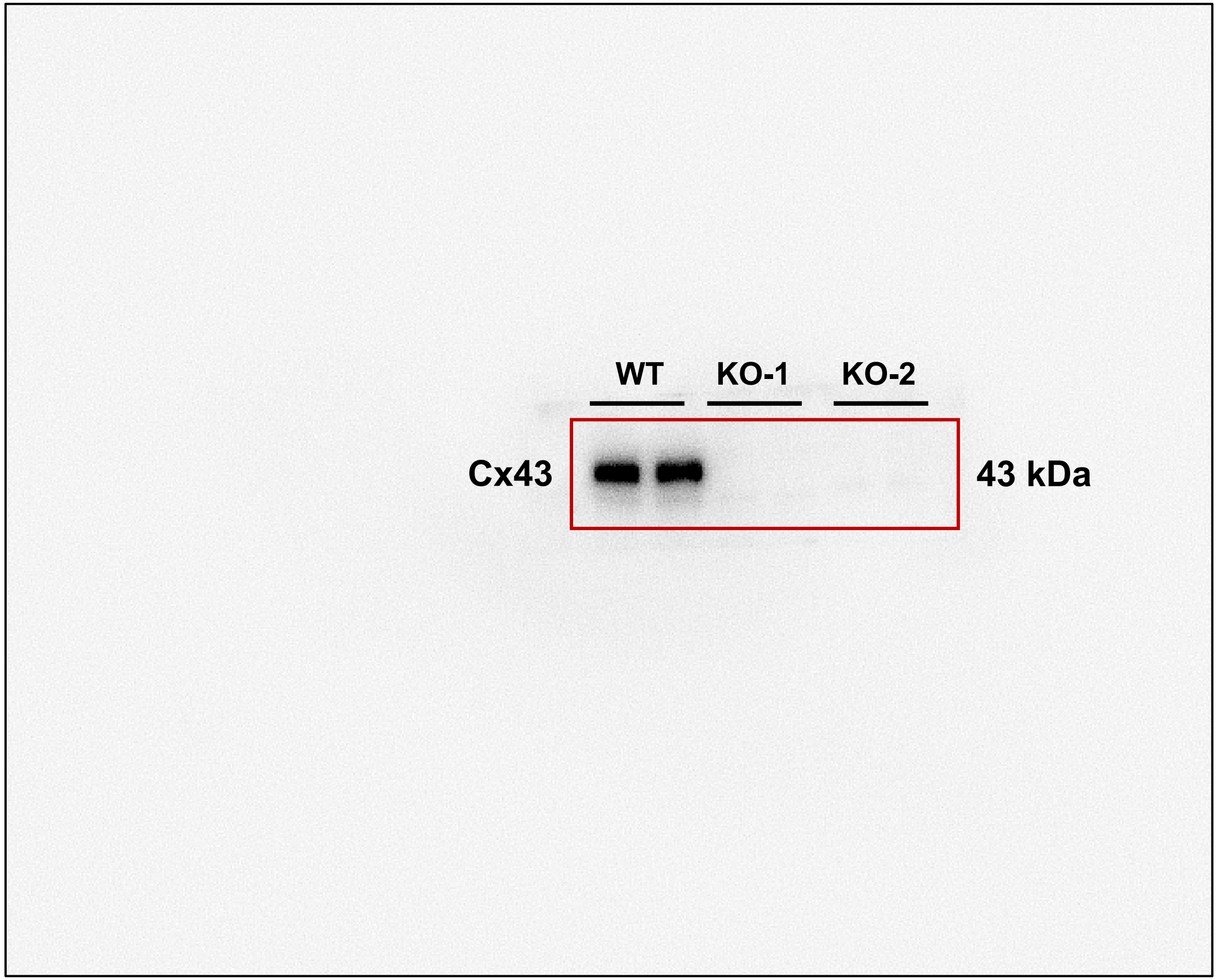

Full length blots of the Cx43 expression in WT and Cx43-KO iPSC-CMs. Red box indicates the cropped blots shown in Figure 2C.

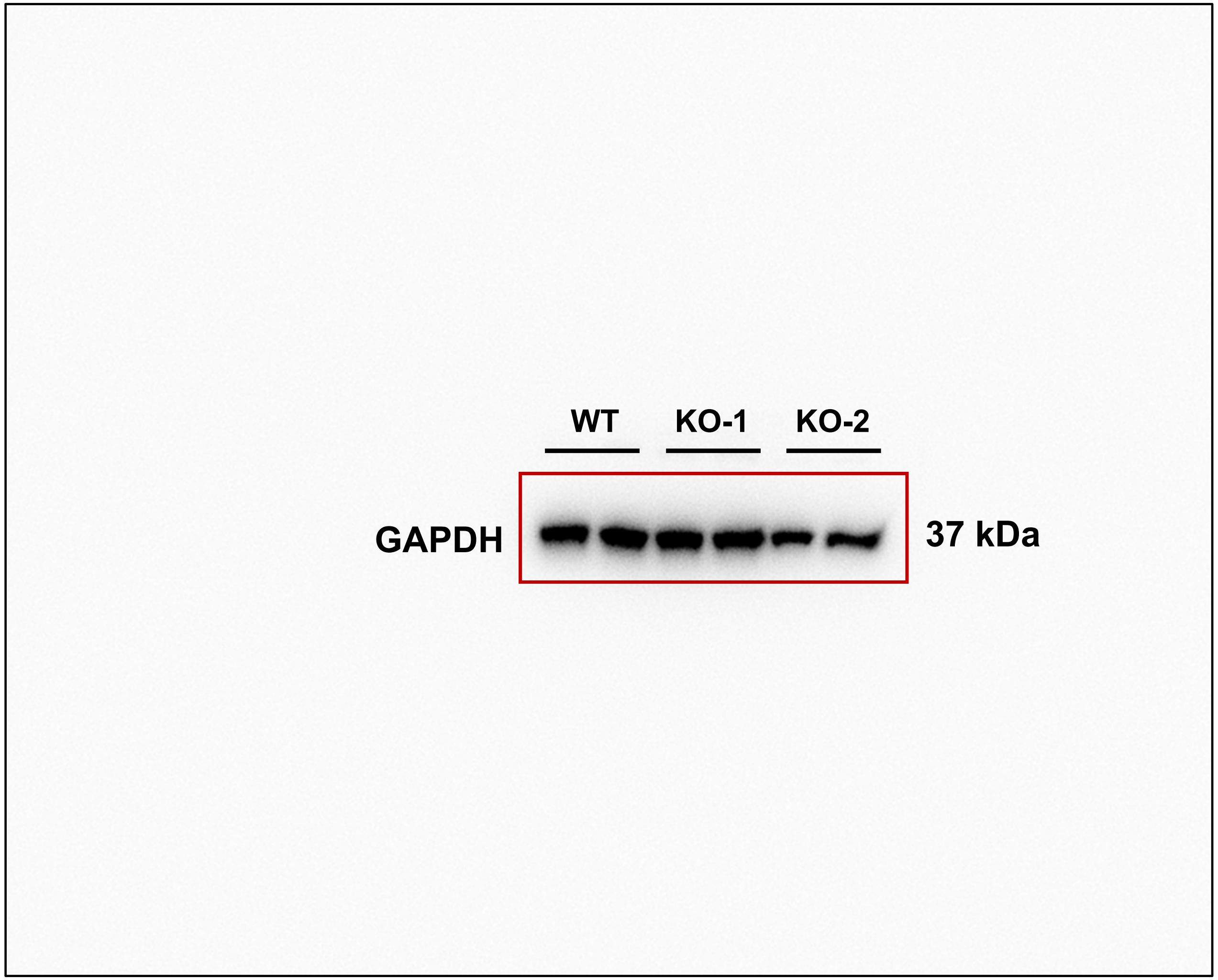

Full length blots of the GAPDH expression in WT and Cx43-KO iPSC-CMs. Red box indicates the cropped blots shown in Figure 2C.

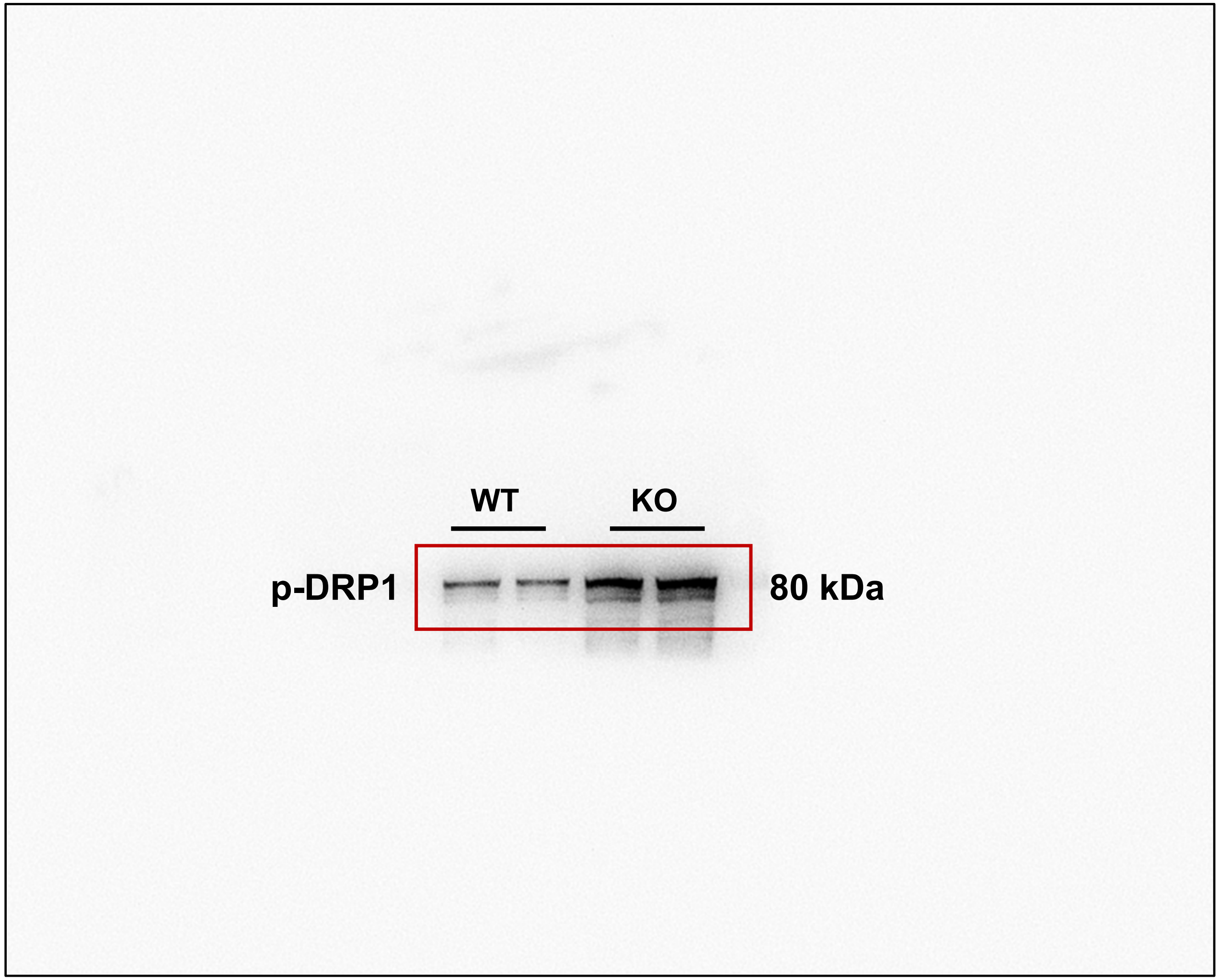

Full length blots of the phosphorylated DRP1 (DRP1-Ser616) expression in WT and Cx43-KO iPSC-CMs. Red box indicates the cropped blots shown in Figure 4H.

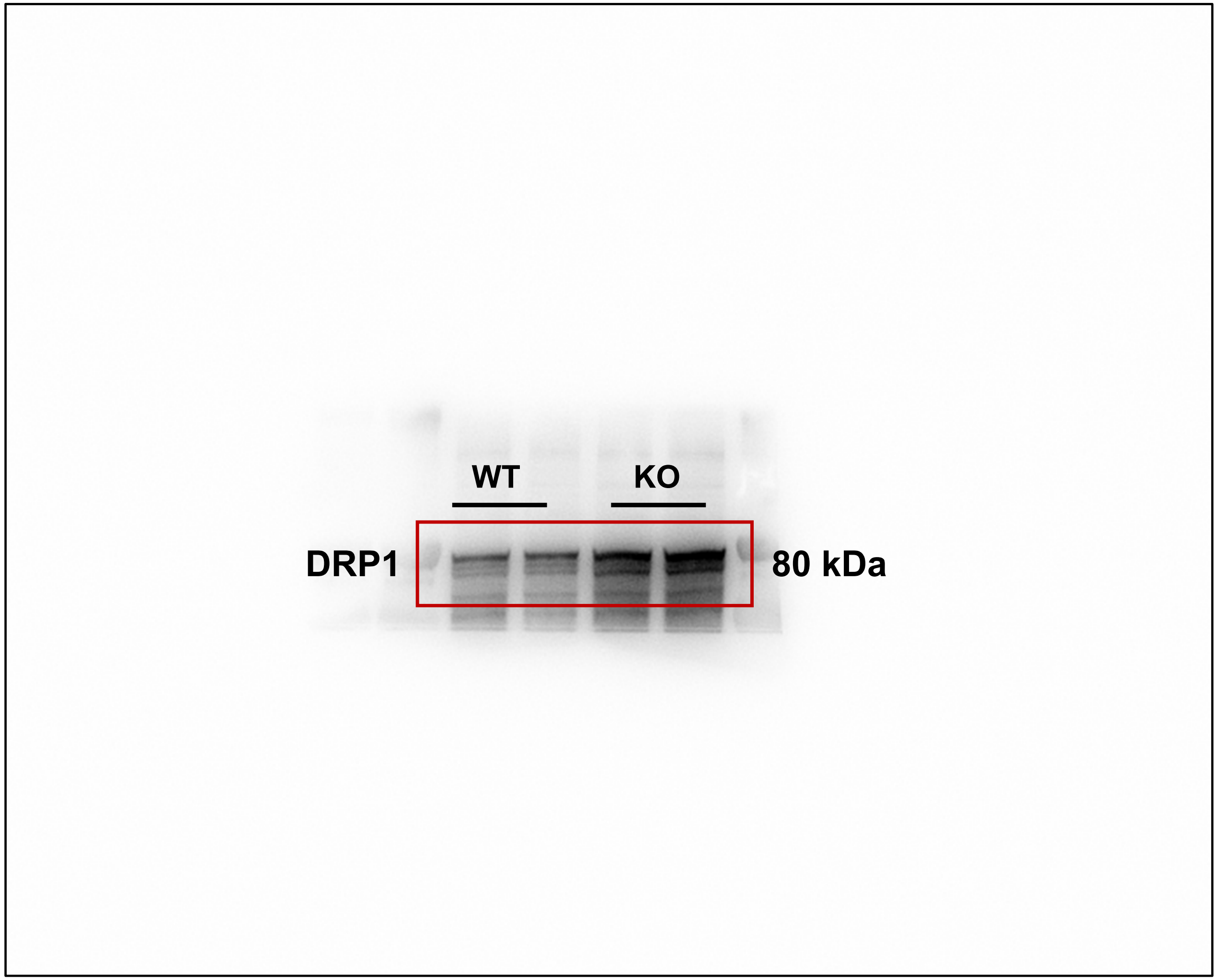

Full length blots of the total DRP1 expression in WT and Cx43-KO iPSC-CMs. Red box indicates the cropped blots shown in Figure 4H.

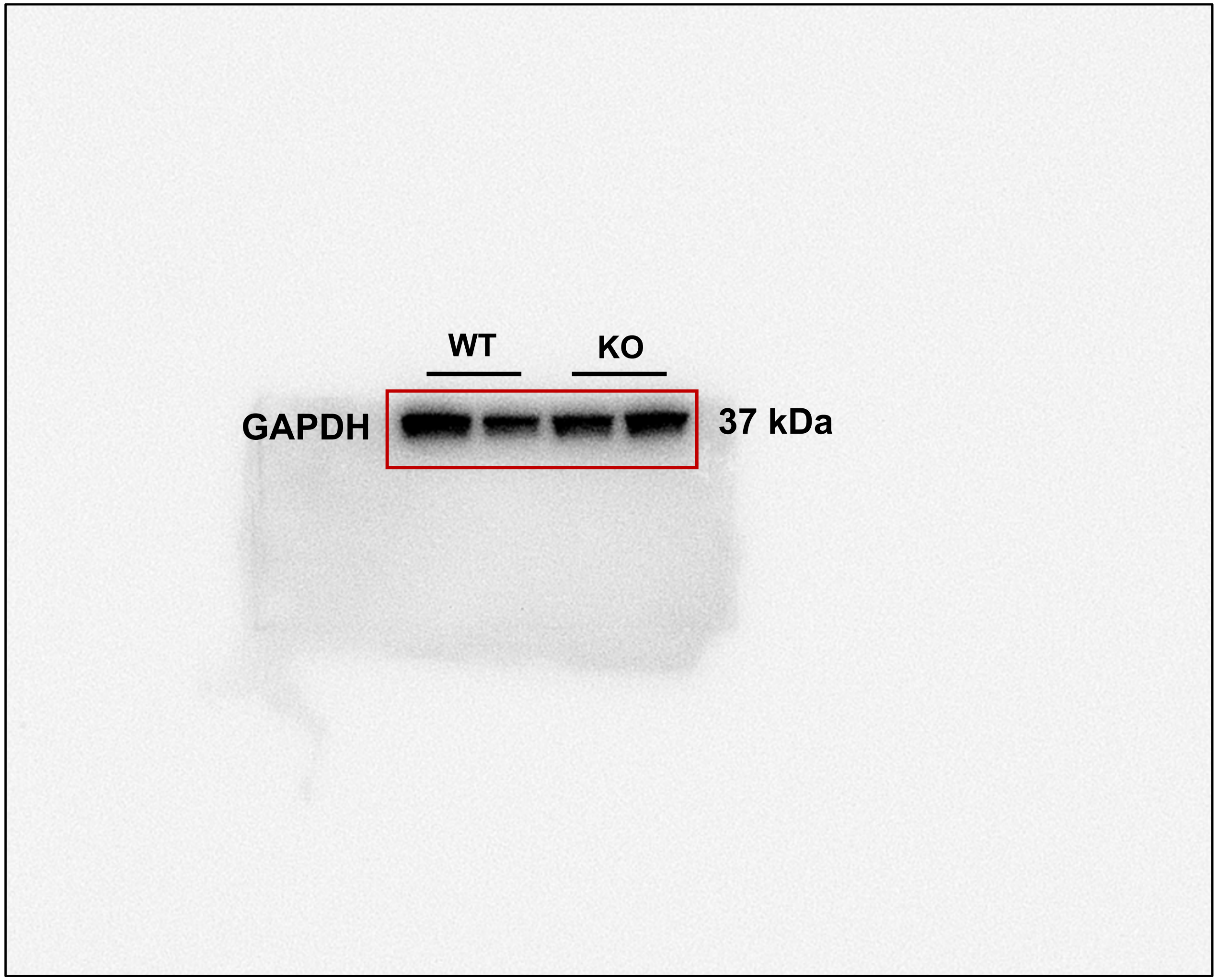

Full length blots of the GAPDH expression in WT and Cx43-KO iPSC-CMs. Red box indicates the cropped blots shown in Figure 4H.

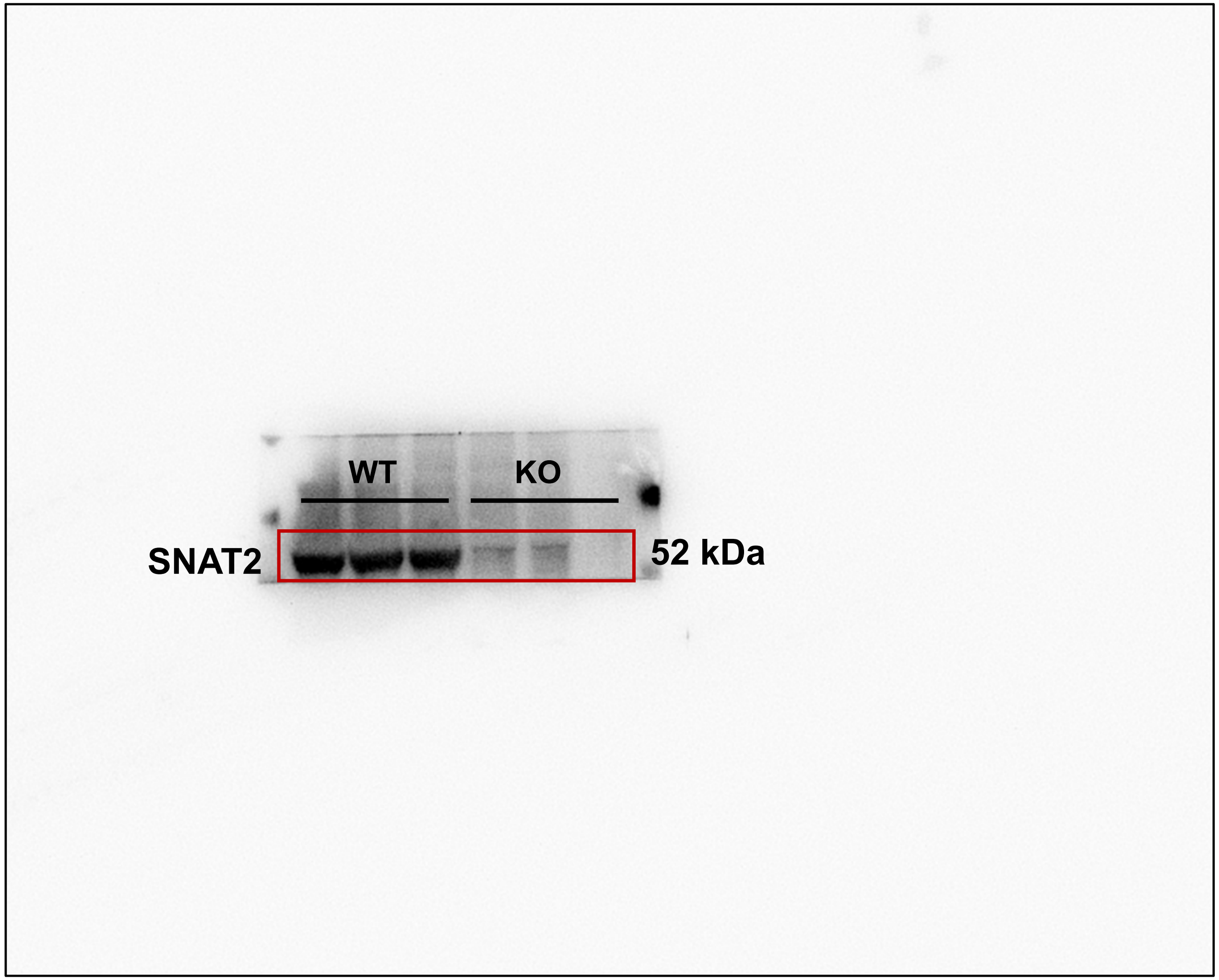

Full length blots of the SNAT2 expression in WT and Cx43-KO iPSC-CMs. Red box indicates the cropped blots shown in Figure 6B.

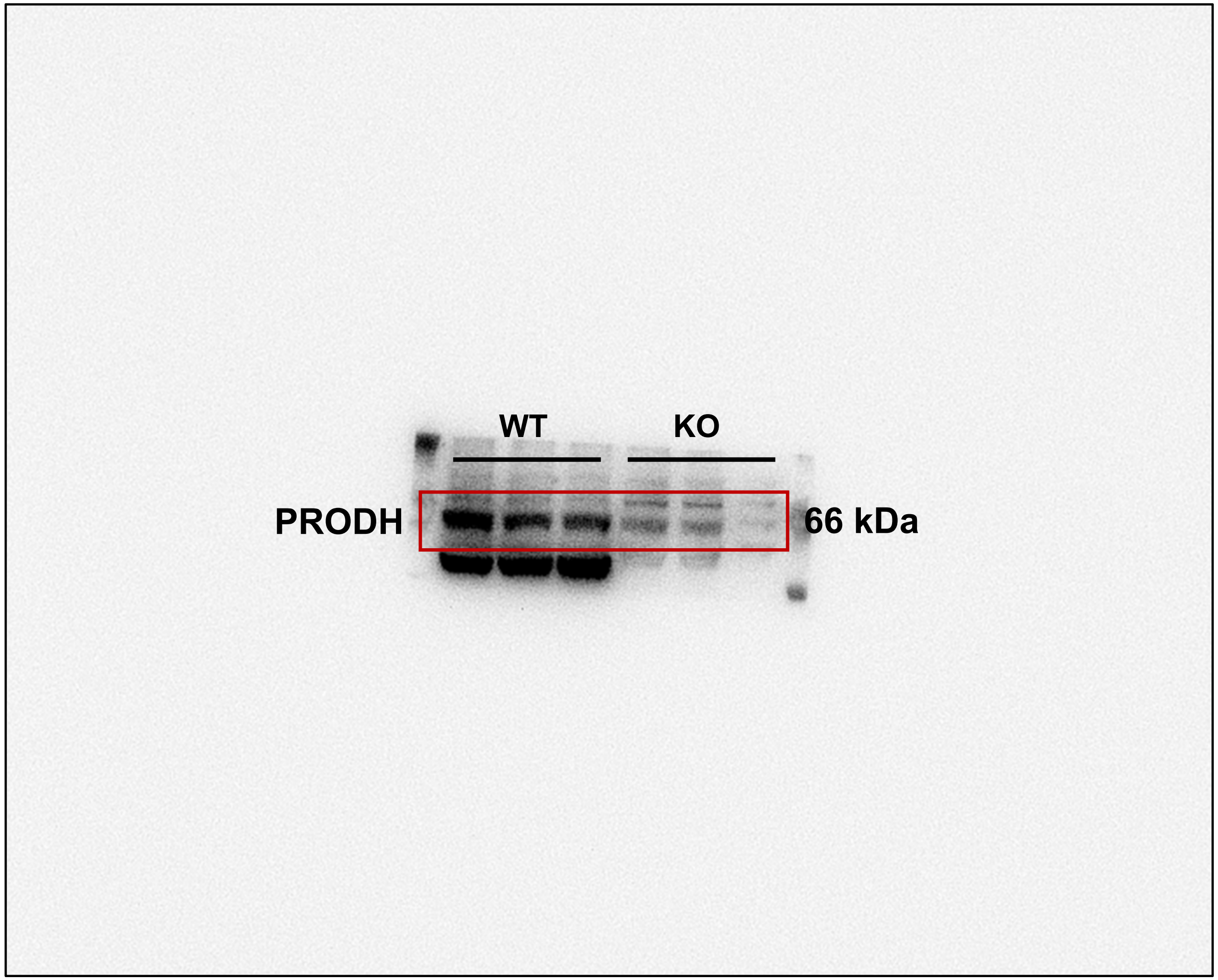

Full length blots of the PRODH expression in WT and Cx43-KO iPSC-CMs. Red box indicates the cropped blots shown in Figure 6B.

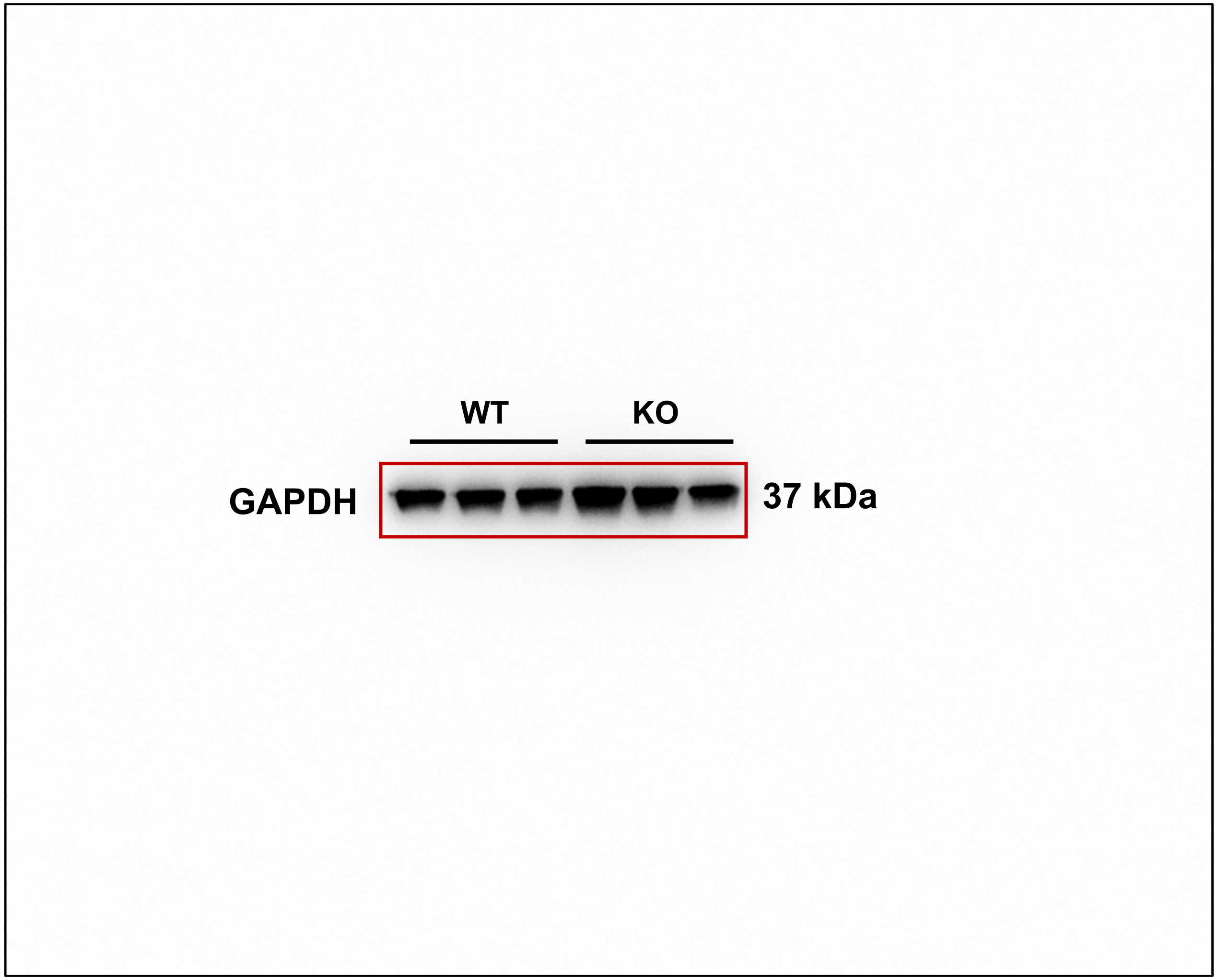

Full length blots of the GAPDH expression in WT and Cx43-KO iPSC-CMs. Red box indicates the cropped blots shown in Figure 6B.

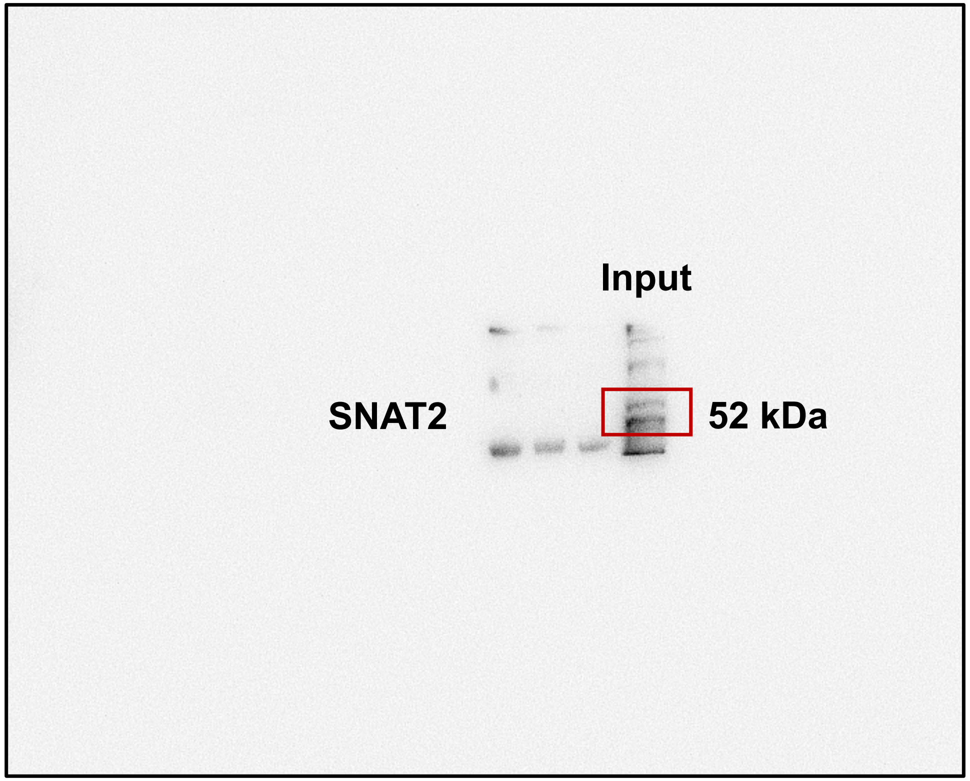

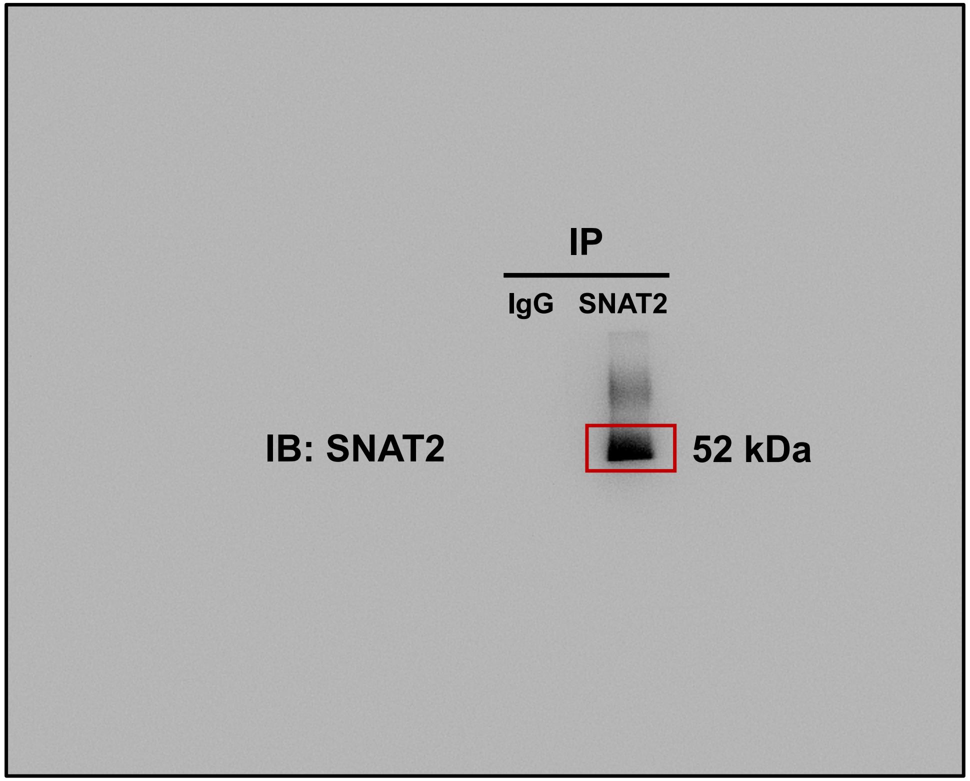

Full length blots of the SNAT2 expression in WT iPSC-CMs. IgG served as the negative control. Red boxes indicate the cropped blots shown in Figure 6E.

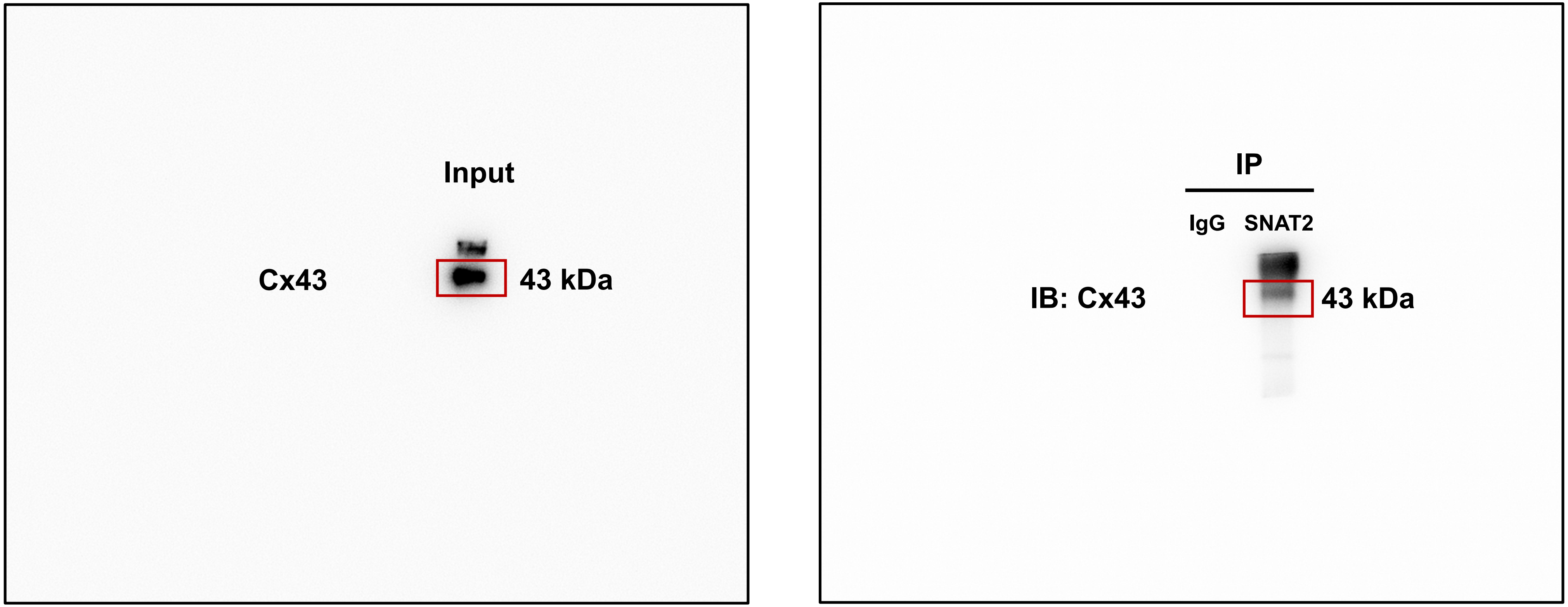

Full length blots of the Cx43 expression in WT iPSC-CMs. IgG served as the negative control. Red boxes indicate the cropped blots shown in Figure 6E.

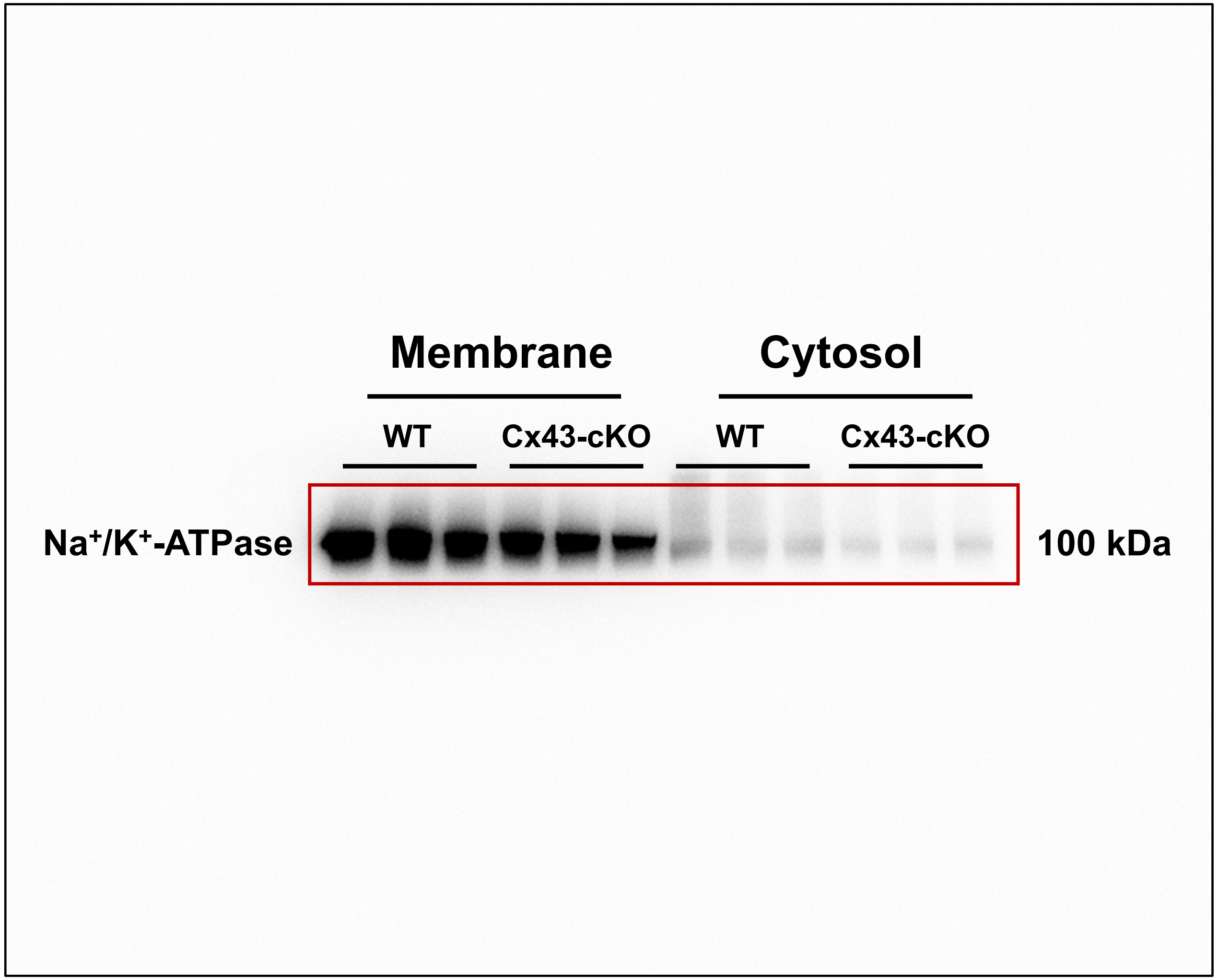

Full length blots of the membrane and cytosol Na^+/K^+-ATPase expression in ventricular tissues collected from 2-month-old WT and HET Cx43-cKO mice. Red box indicates the cropped blots shown in Figure 7A.

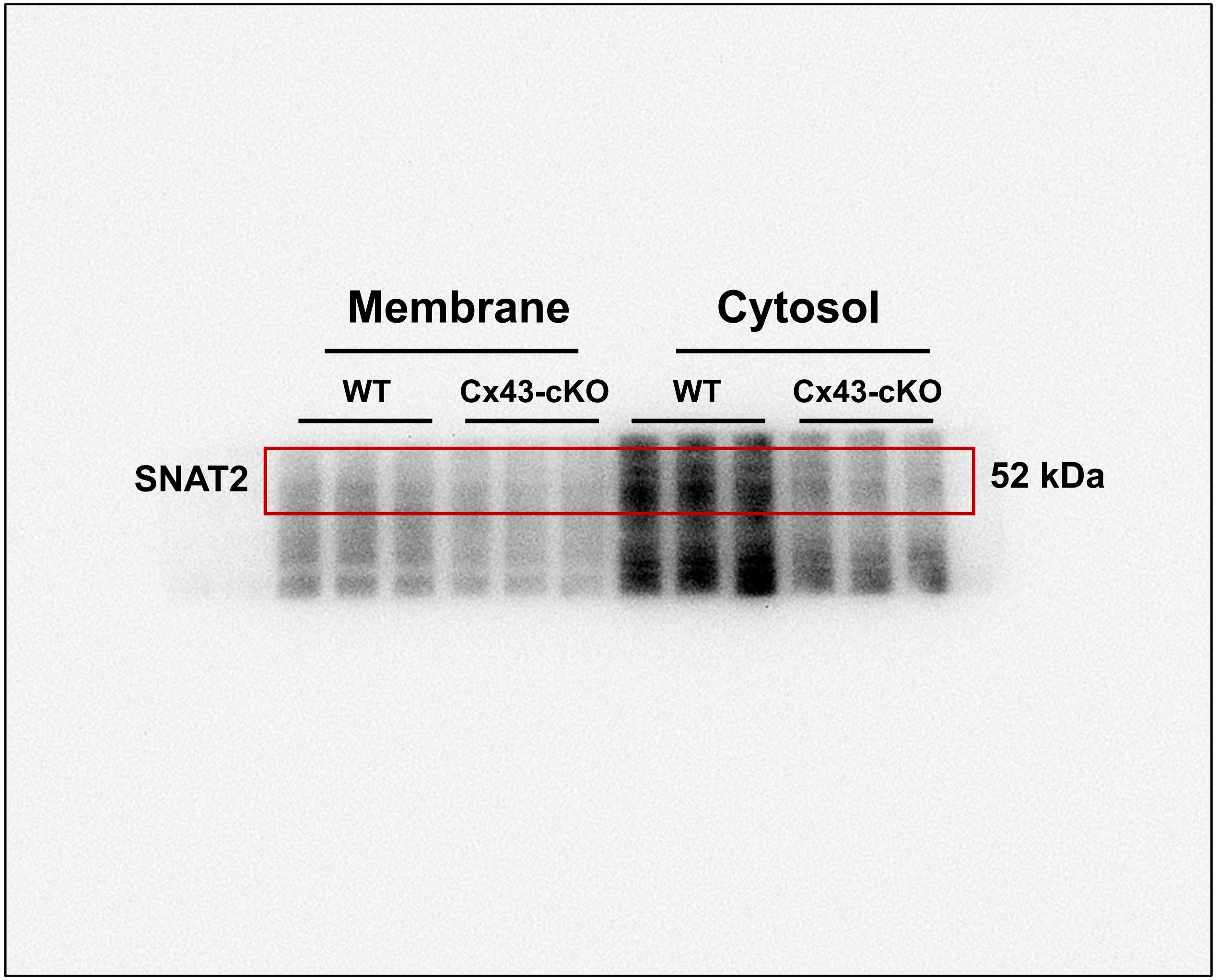

Full length blots of the membrane and cytosol SNAT2 expression in ventricular tissues collected from 2-month-old WT and HET Cx43-cKO mice. Red box indicates the cropped blots shown in Figure 7A.

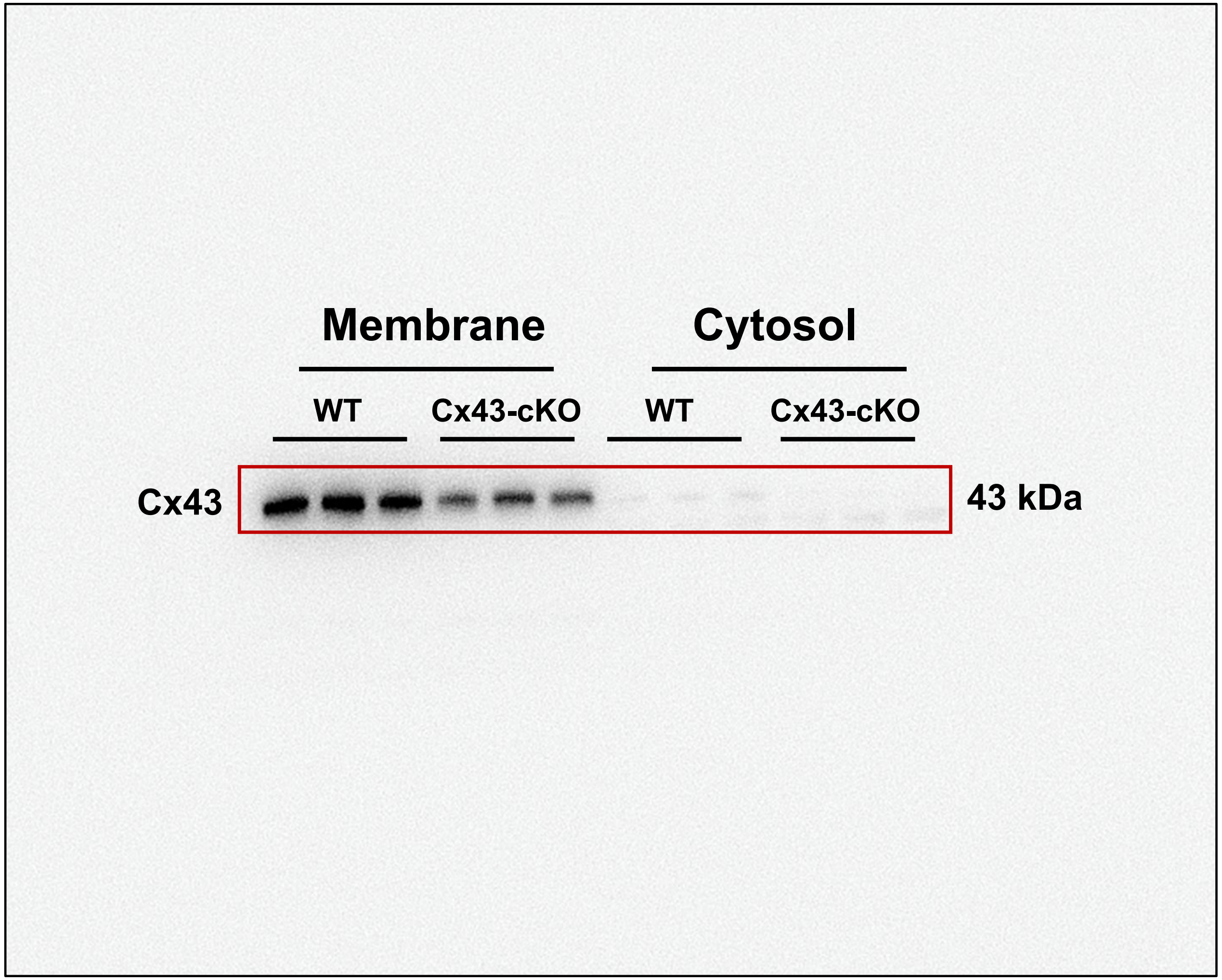

Full length blots of the membrane and cytosol Cx43 expression in ventricular tissues collected from 2-month-old WT and HET Cx43-cKO mice. Red box indicates the cropped blots shown in Figure 7A.

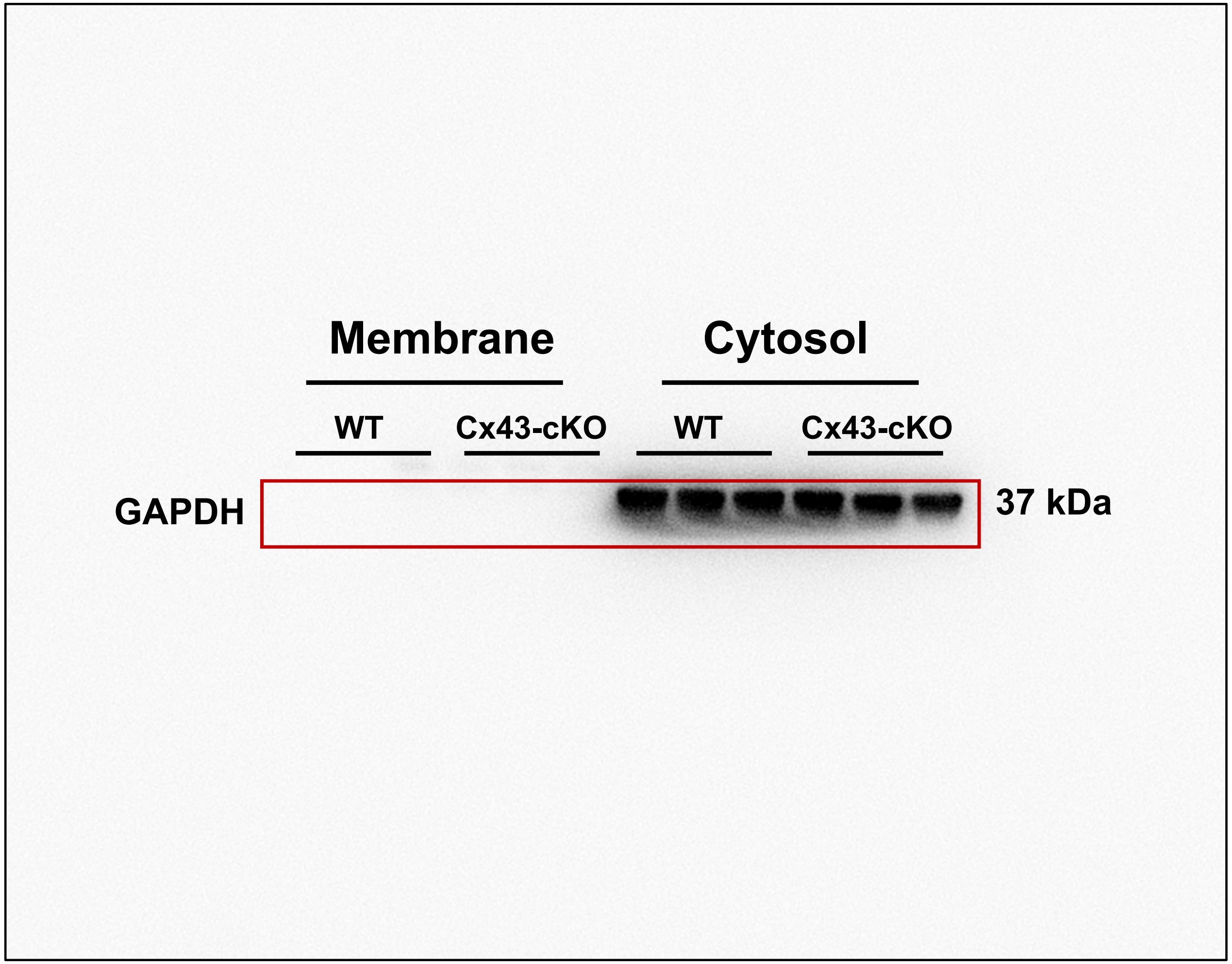

Full length blots of the membrane and cytosol GAPDH expression in ventricular tissues collected from 2-month-old WT and HET Cx43-cKO mice. Red box indicates the cropped blots shown in Figure 7A.

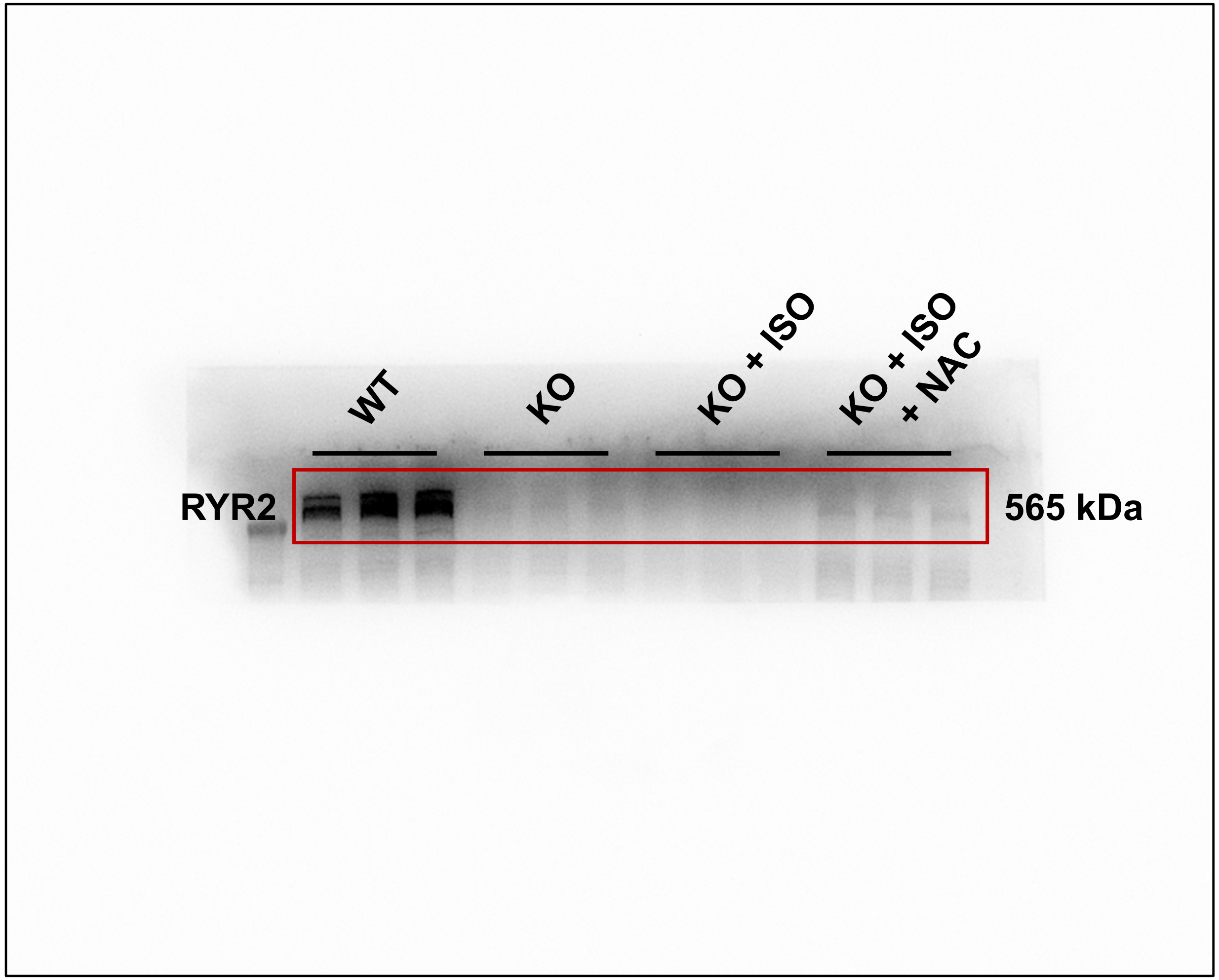

Full length blots of the RYR2 expression in WT and Cx43-KO iPSC-CMs with or without ISO stimulation, and with or without NAC treatment. Red box indicates the cropped blots shown in Supplemental Figure 8J.

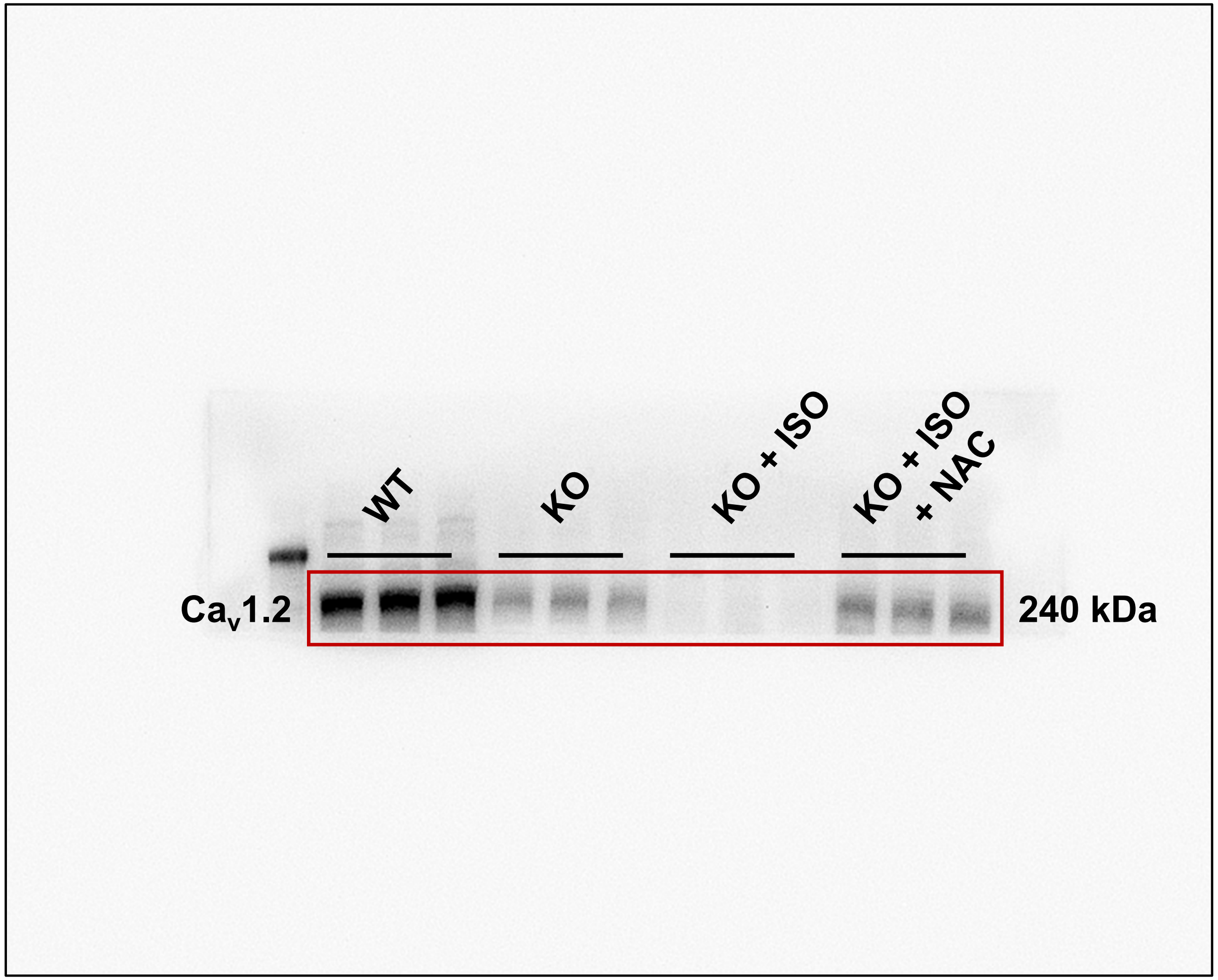

Full length blots of the $Ca_v1.2$ expression in WT and Cx43-KO iPSC-CMs with or without ISO stimulation, and with or without NAC treatment. Red box indicates the cropped blots shown in Supplemental Figure 8J.

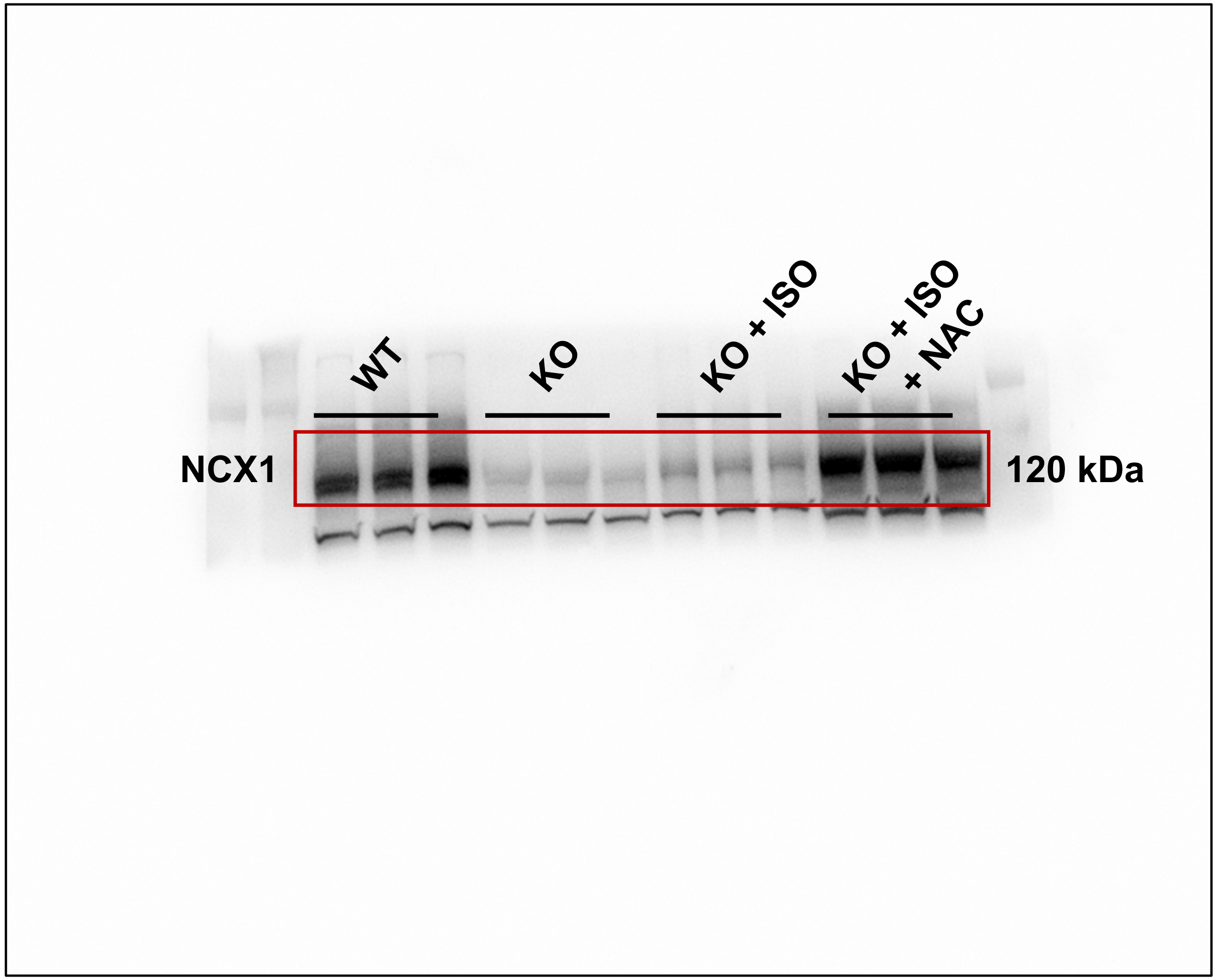

Full length blots of the NCX1 expression in WT and Cx43-KO iPSC-CMs with or without ISO stimulation, and with or without NAC treatment. Red box indicates the cropped blots shown in Supplemental Figure 8J.

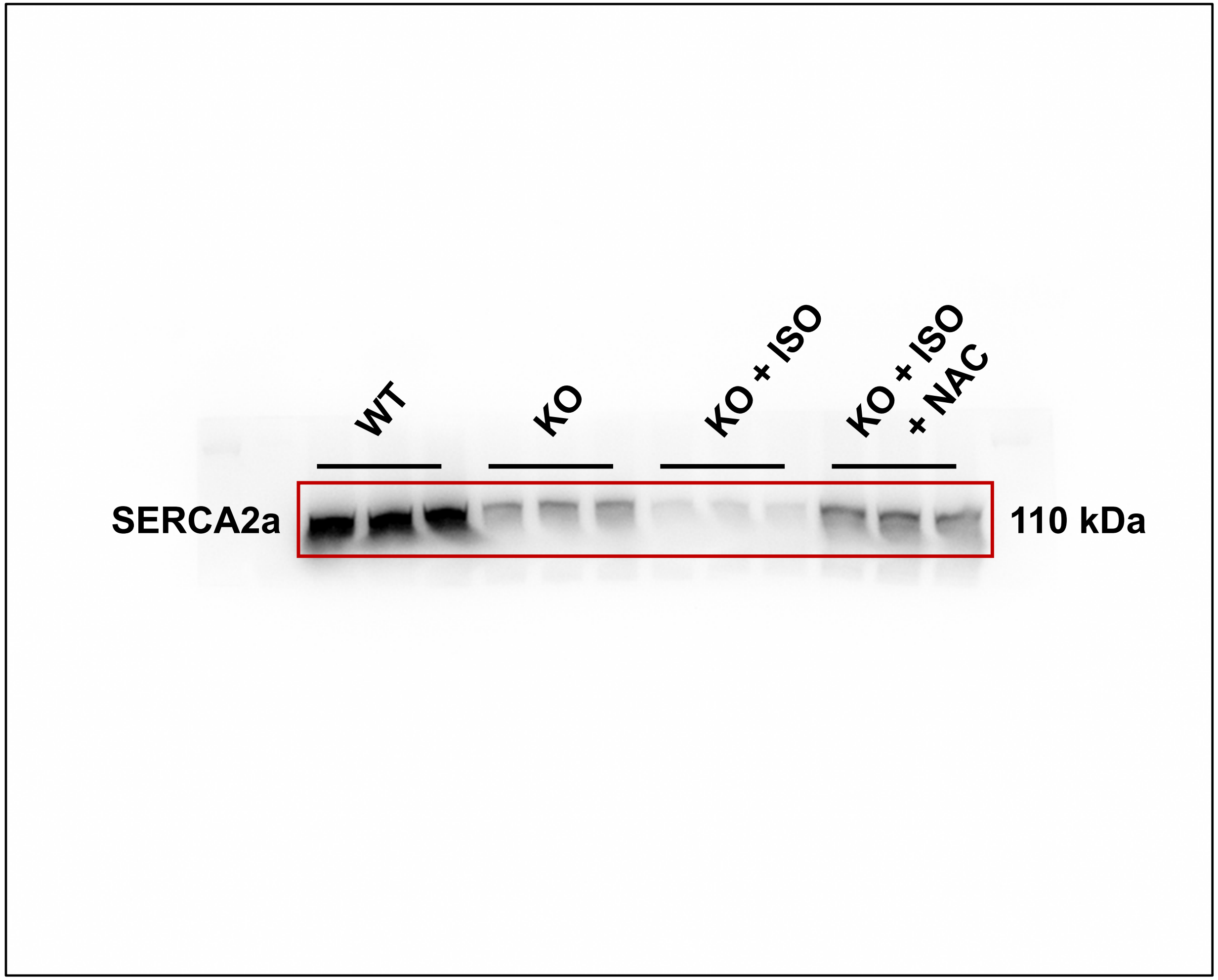

Full length blots of the SERCA2a expression in WT and Cx43-KO iPSC-CMs with or without ISO stimulation, and with or without NAC treatment. Red box indicates the cropped blots shown in Supplemental Figure 8J.

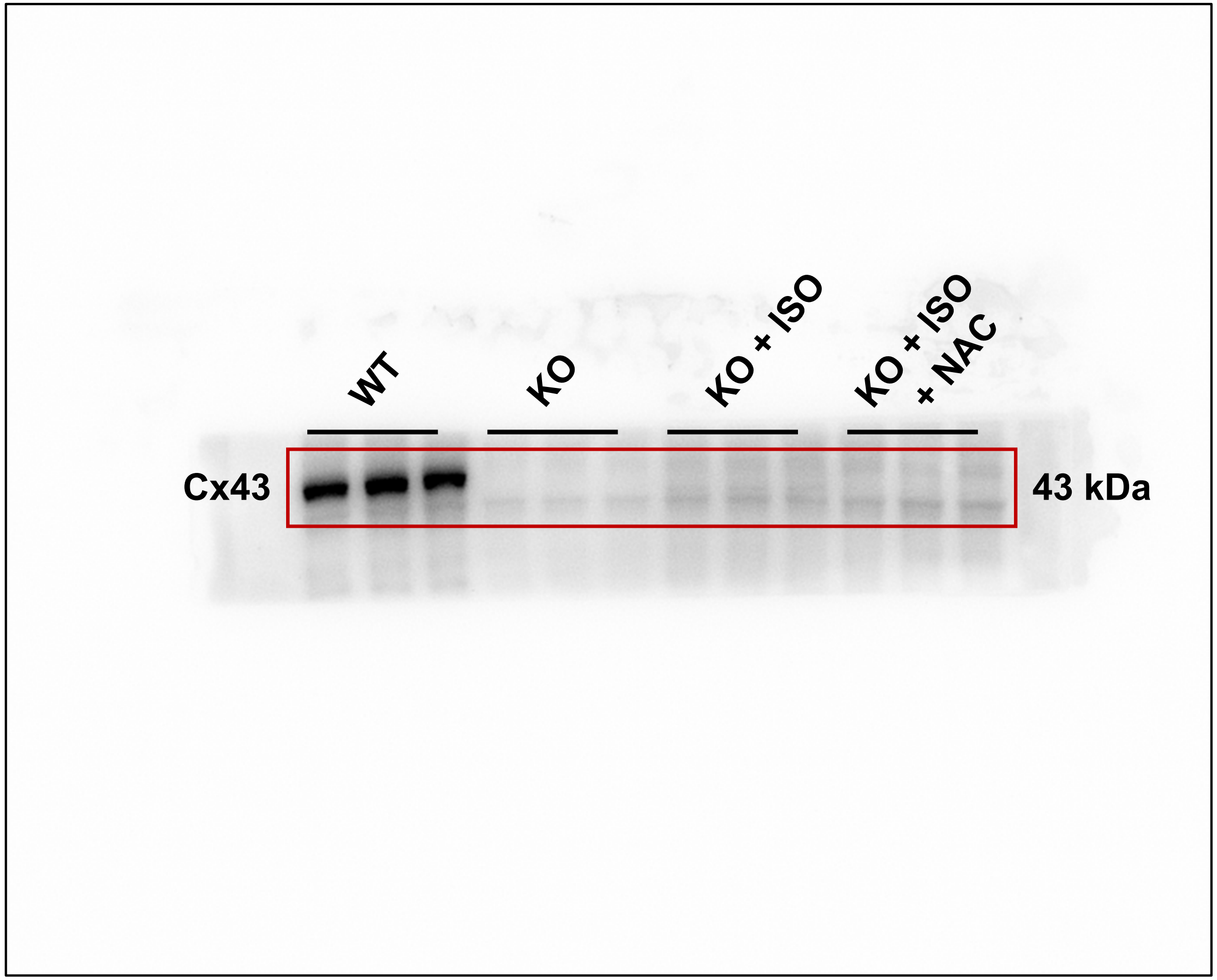

Full length blots of the Cx43 expression in WT and Cx43-KO iPSC-CMs with or without ISO stimulation, and with or without NAC treatment. Red box indicates the cropped blots shown in Supplemental Figure 8J.

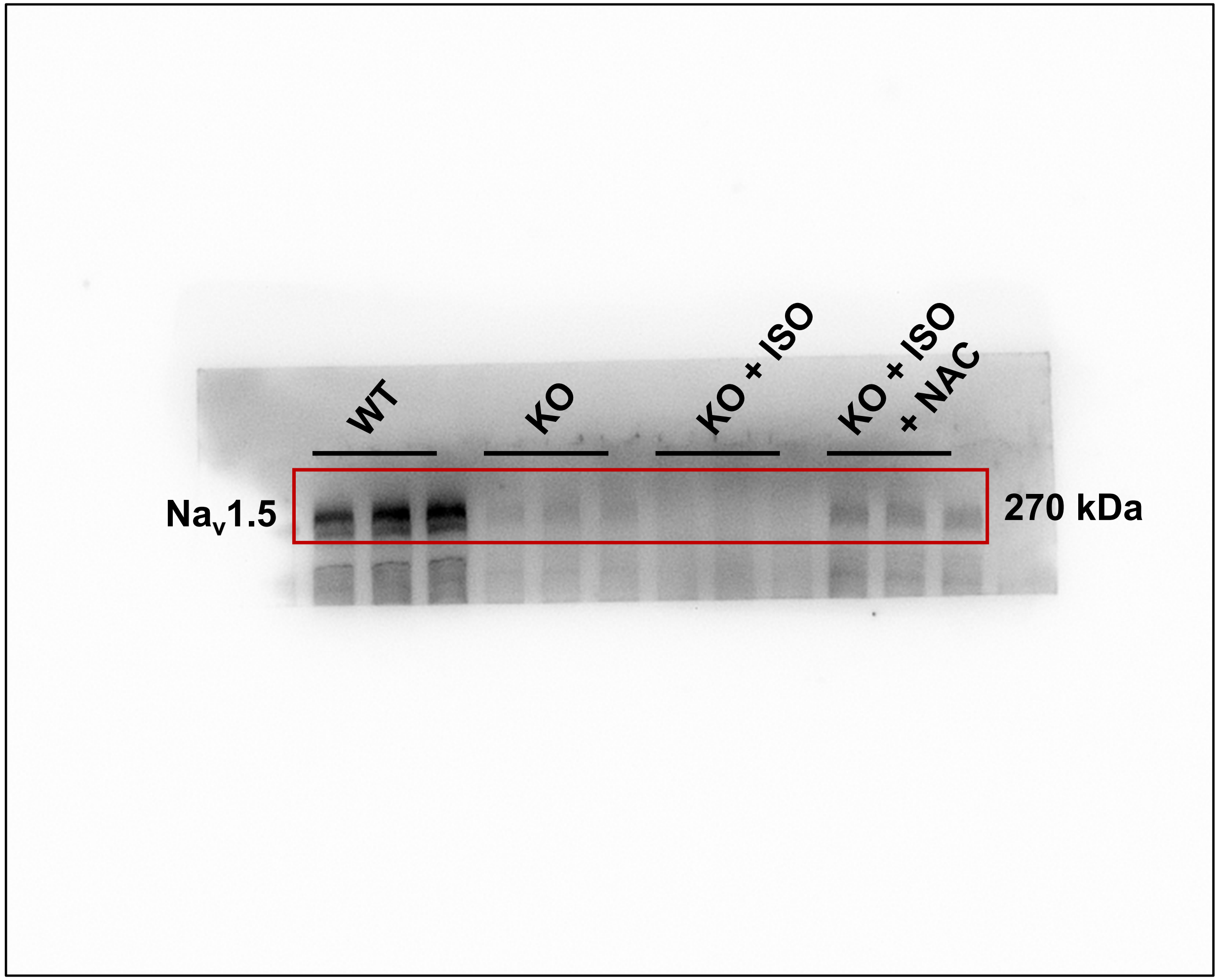

Full length blots of the $Na_v1.5$ expression in WT and Cx43-KO iPSC-CMs with or without ISO stimulation, and with or without NAC treatment. Red box indicates the cropped blots shown in Supplemental Figure 8J.

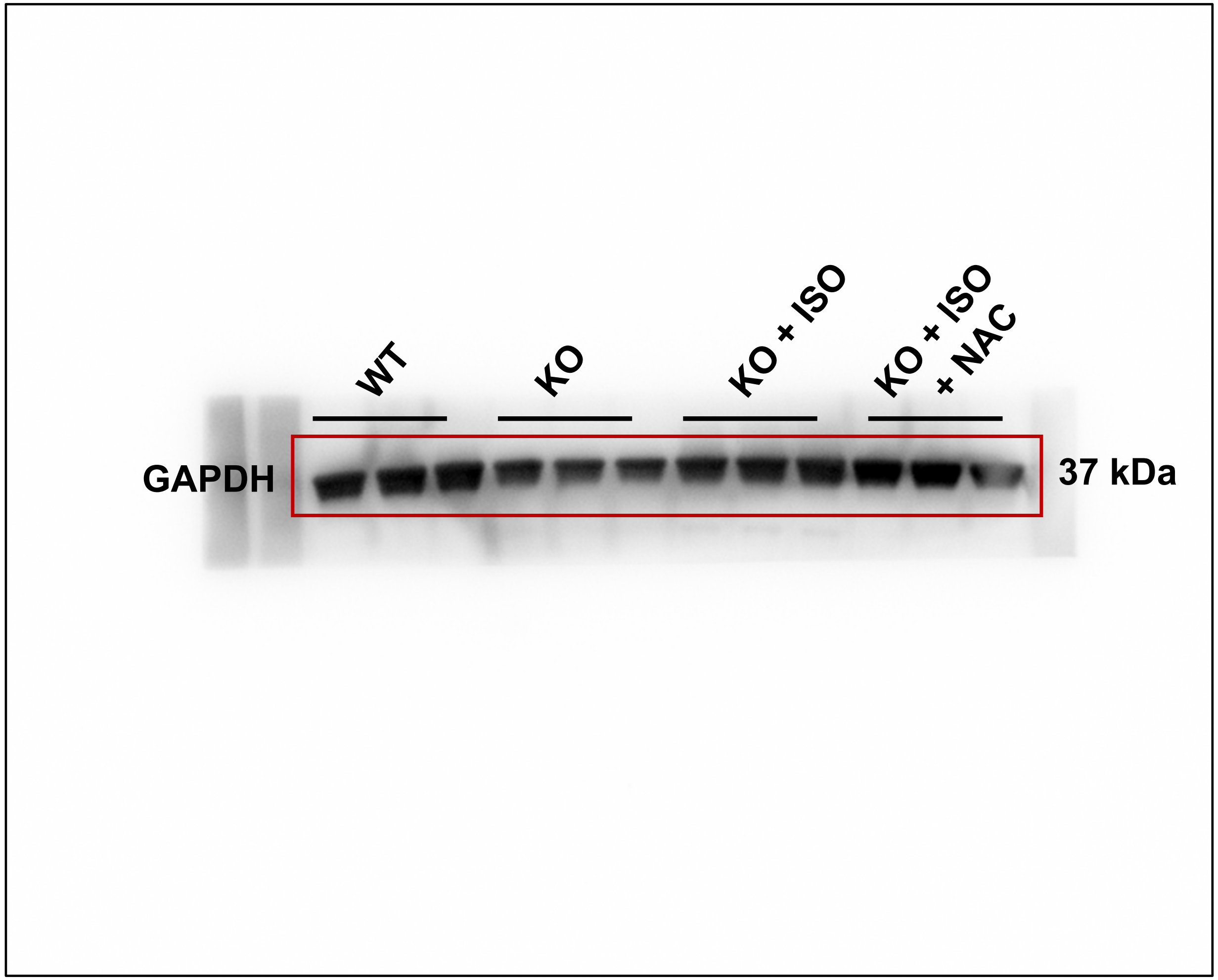

Full length blots of the GAPDH expression in WT and Cx43-KO iPSC-CMs with or without ISO stimulation, and with or without NAC treatment. Red box indicates the cropped blots shown in Supplemental Figure 8J.

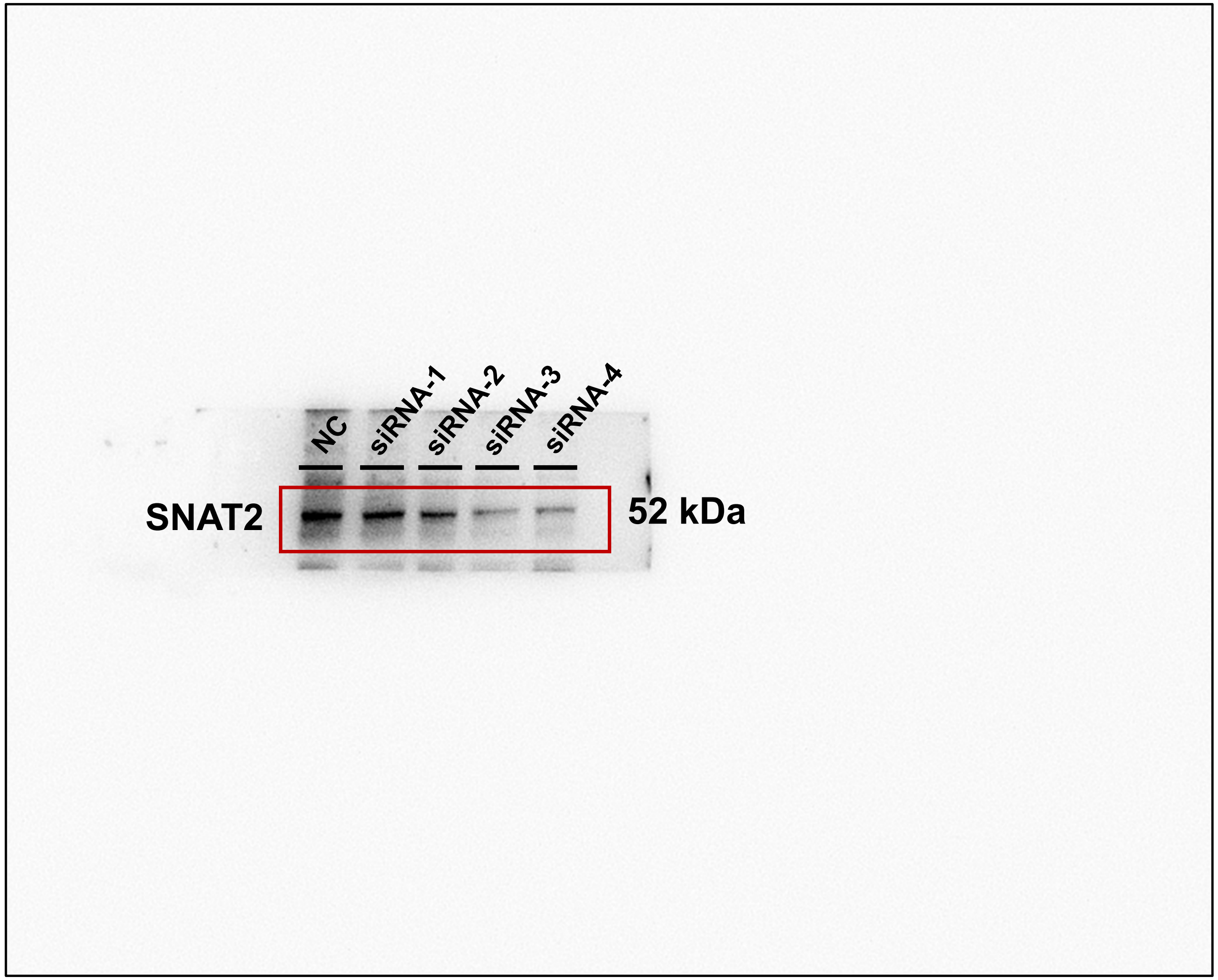

Full length blots of the SNAT2 expression in HEK293 cells transfected with scrambled siRNA (NC) and four different SNAT2 siRNAs. Red box indicates the cropped blots shown in Supplemental Figure 9H.

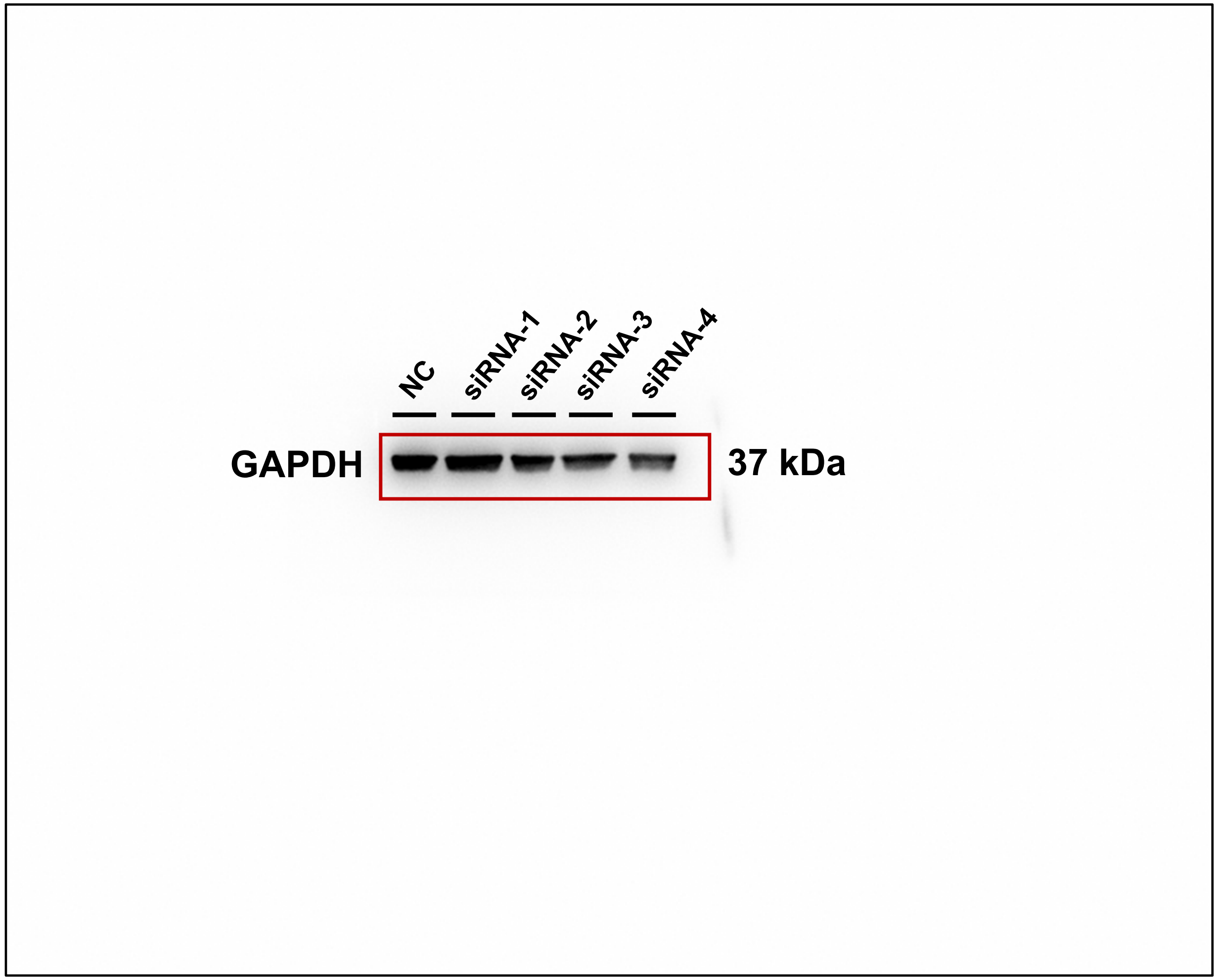

Full length blots of the GAPDH expression in HEK293 cells transfected with scrambled siRNA (NC) and four different SNAT2 siRNAs. Red box indicates the cropped blots shown in Supplemental Figure 9H.

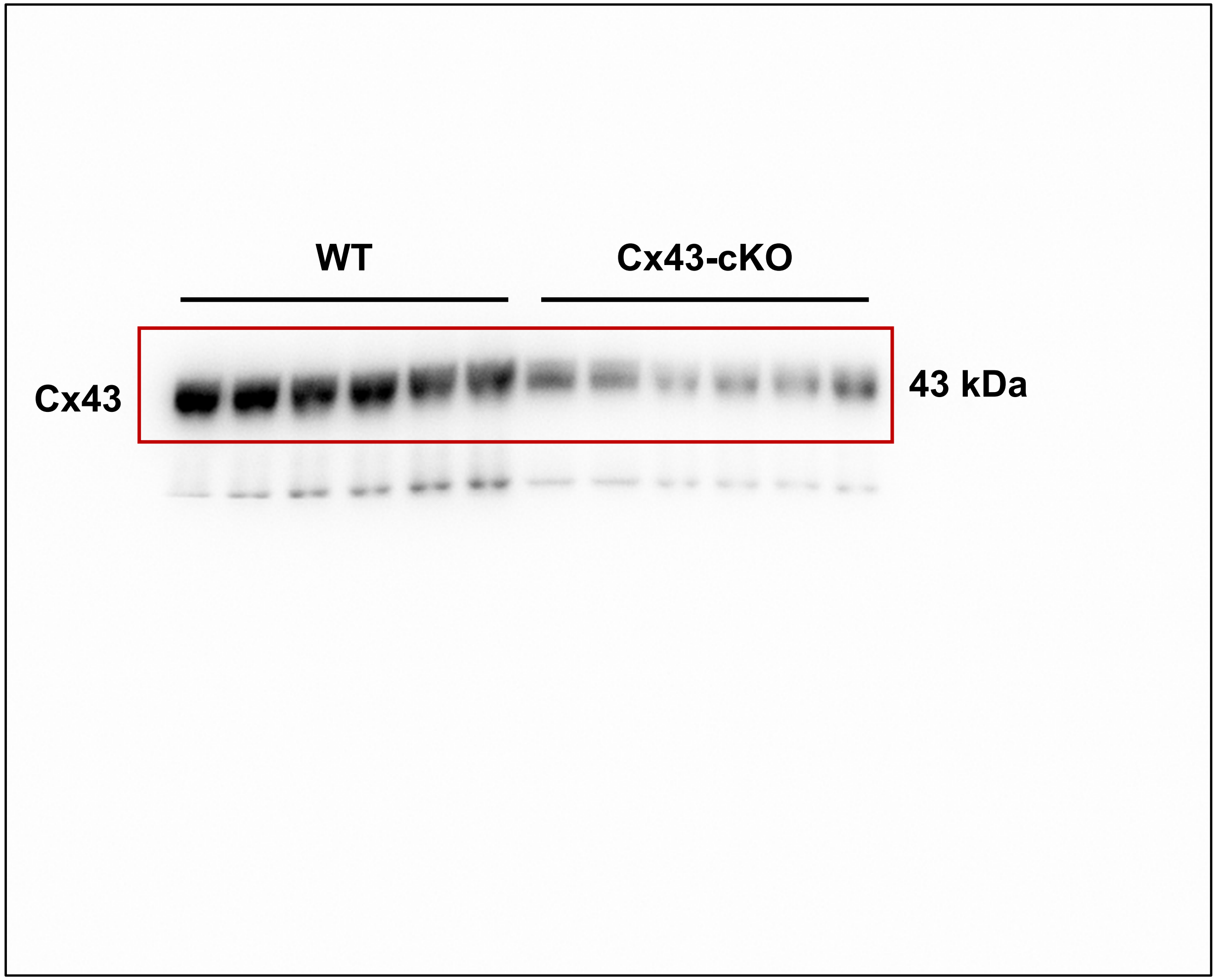

Full length blots of the Cx43 expression in ventricular tissues collected from 2-month-old WT and HET Cx43-cKO mice. Red box indicates the cropped blots shown in Supplemental Figure 10C.

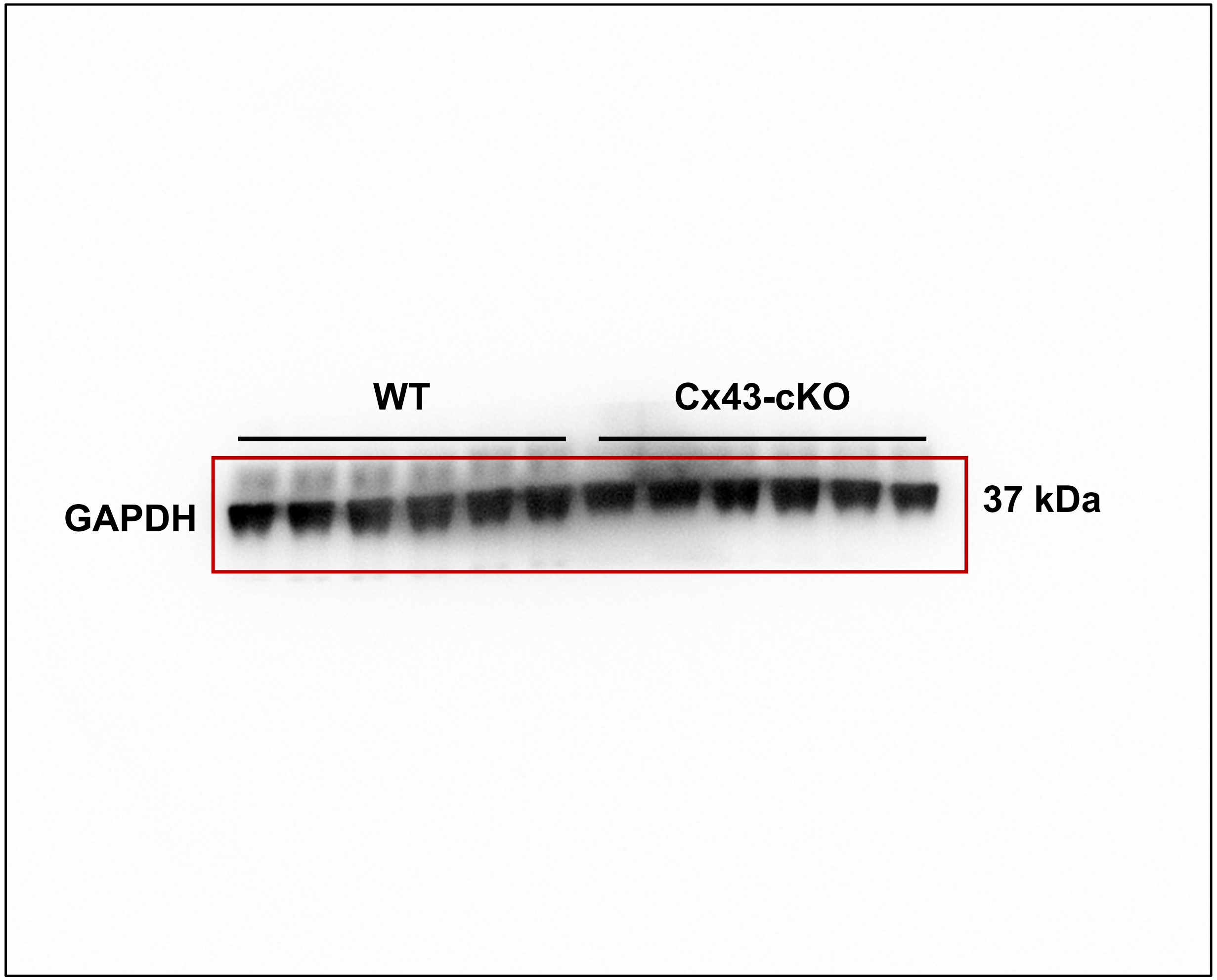

Full length blots of the GAPDH expression in ventricular tissues collected from 2-month-old WT and HET Cx43-cKO mice. Red box indicates the cropped blots shown in Supplemental Figure 10C.

Membrane

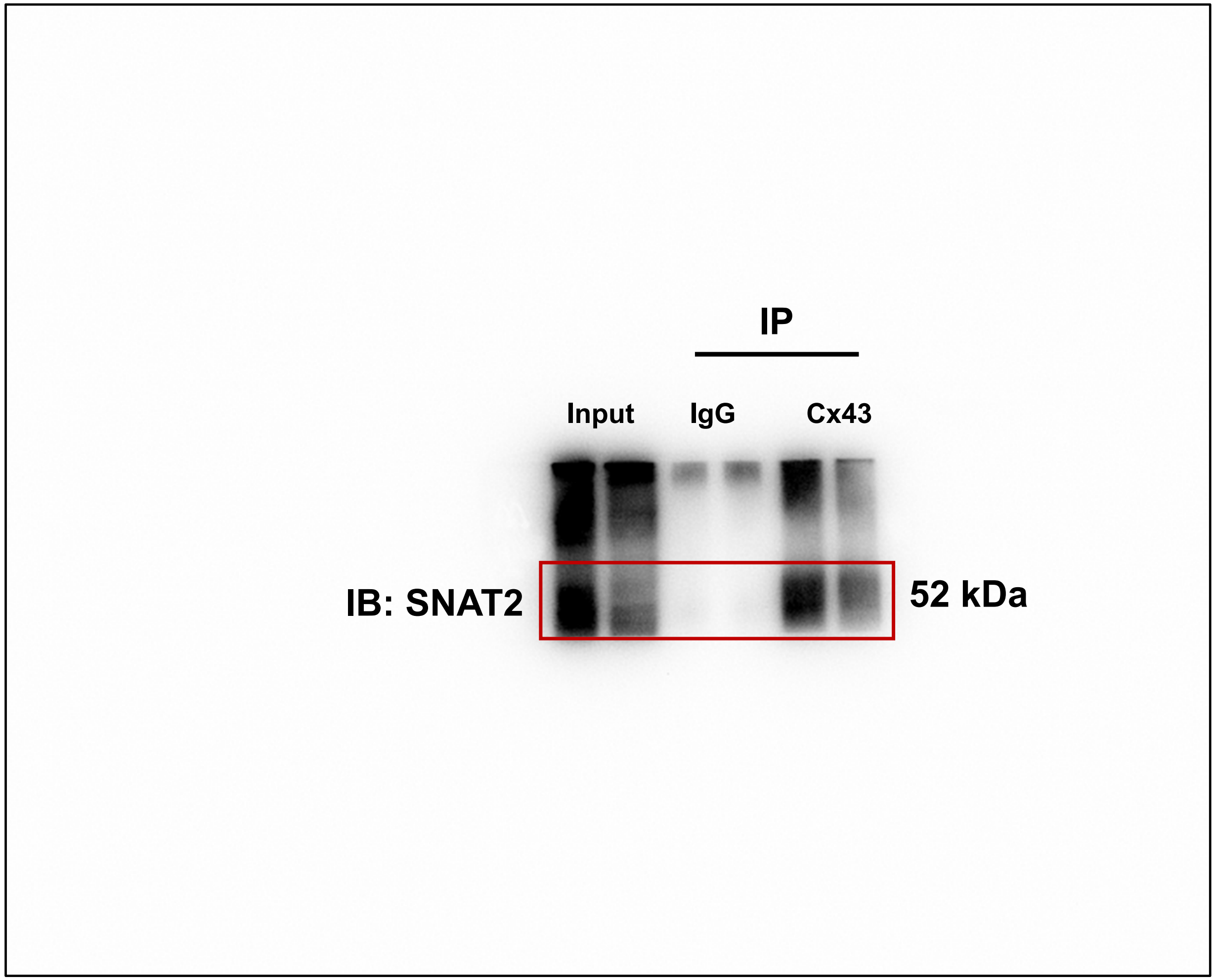

Full length blots of the SNAT2 expression on the cell membrane of the 2-month-old WT mouse ventricular tissues. IgG served as the negative control. Red box indicates the cropped blots shown in Supplemental Figure 11D.

Membrane

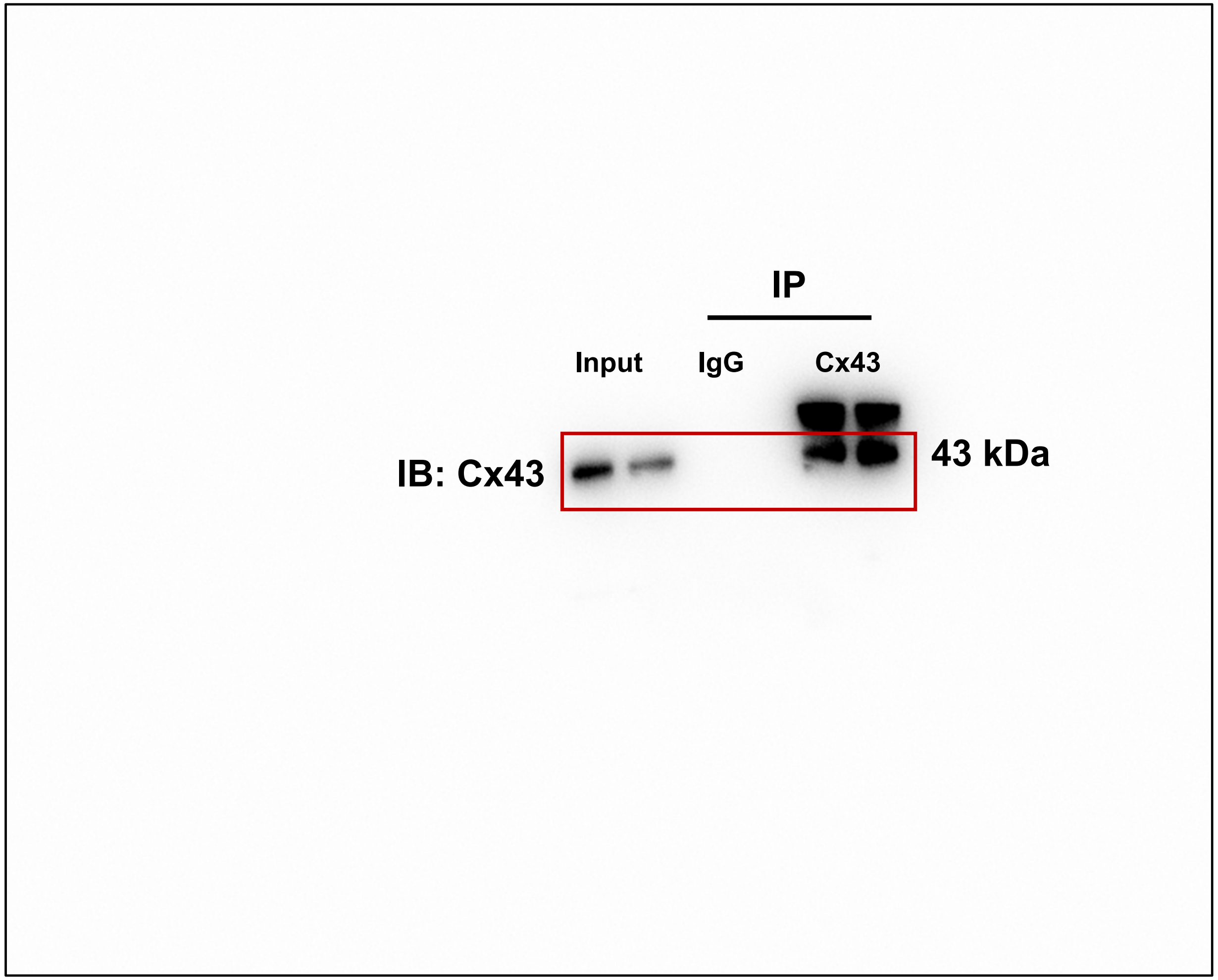

Full length blots of the Cx43 expression on the cell membrane of the 2-month-old WT mouse ventricular tissues. IgG served as the negative control. Red box indicates the cropped blots shown in Supplemental Figure 11D.

Membrane

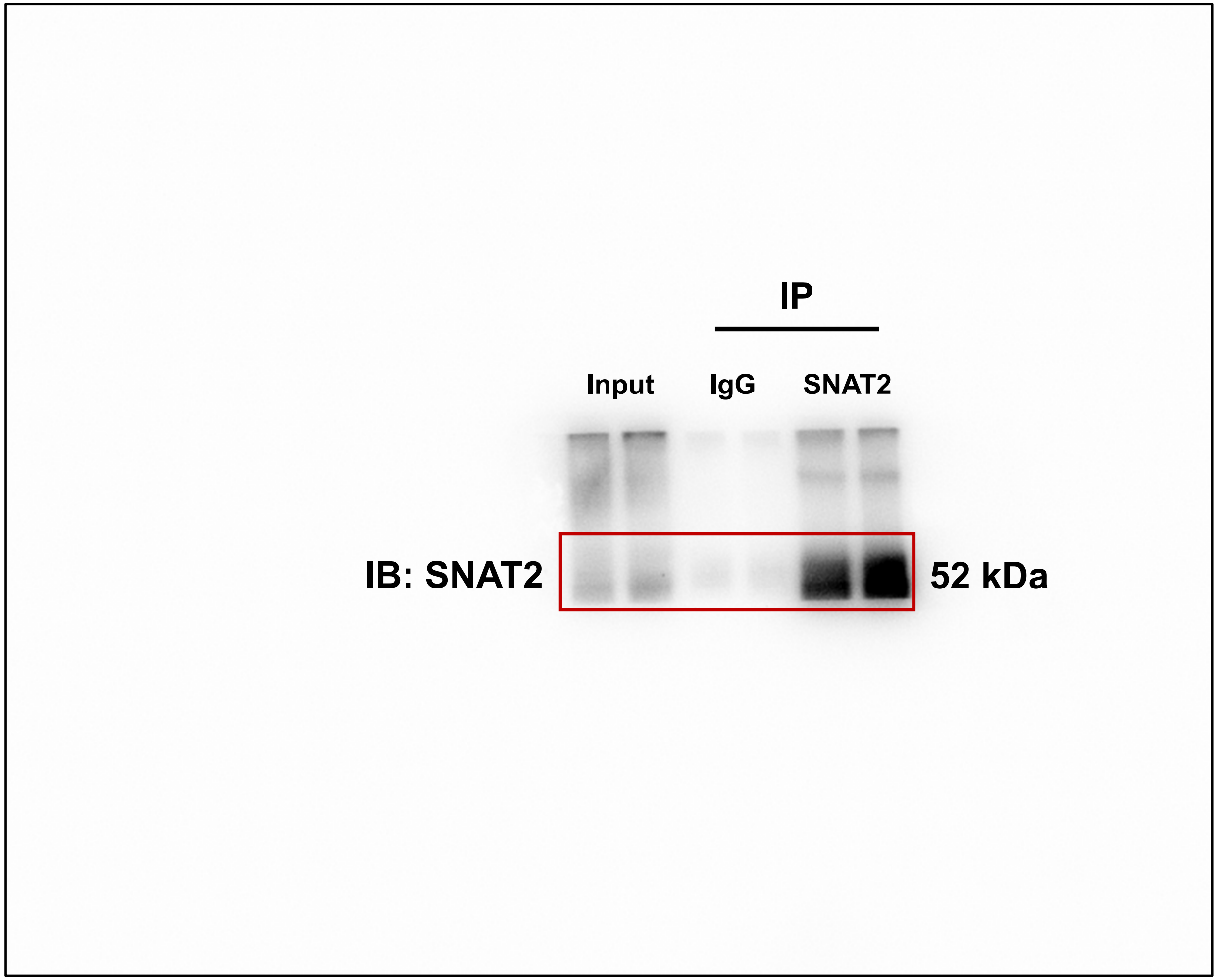

Full length blots of the SNAT2 expression on the cell membrane of the 2-month-old WT mouse ventricular tissues. IgG served as the negative control. Red box indicates the cropped blots shown in Supplemental Figure 11D.

Membrane

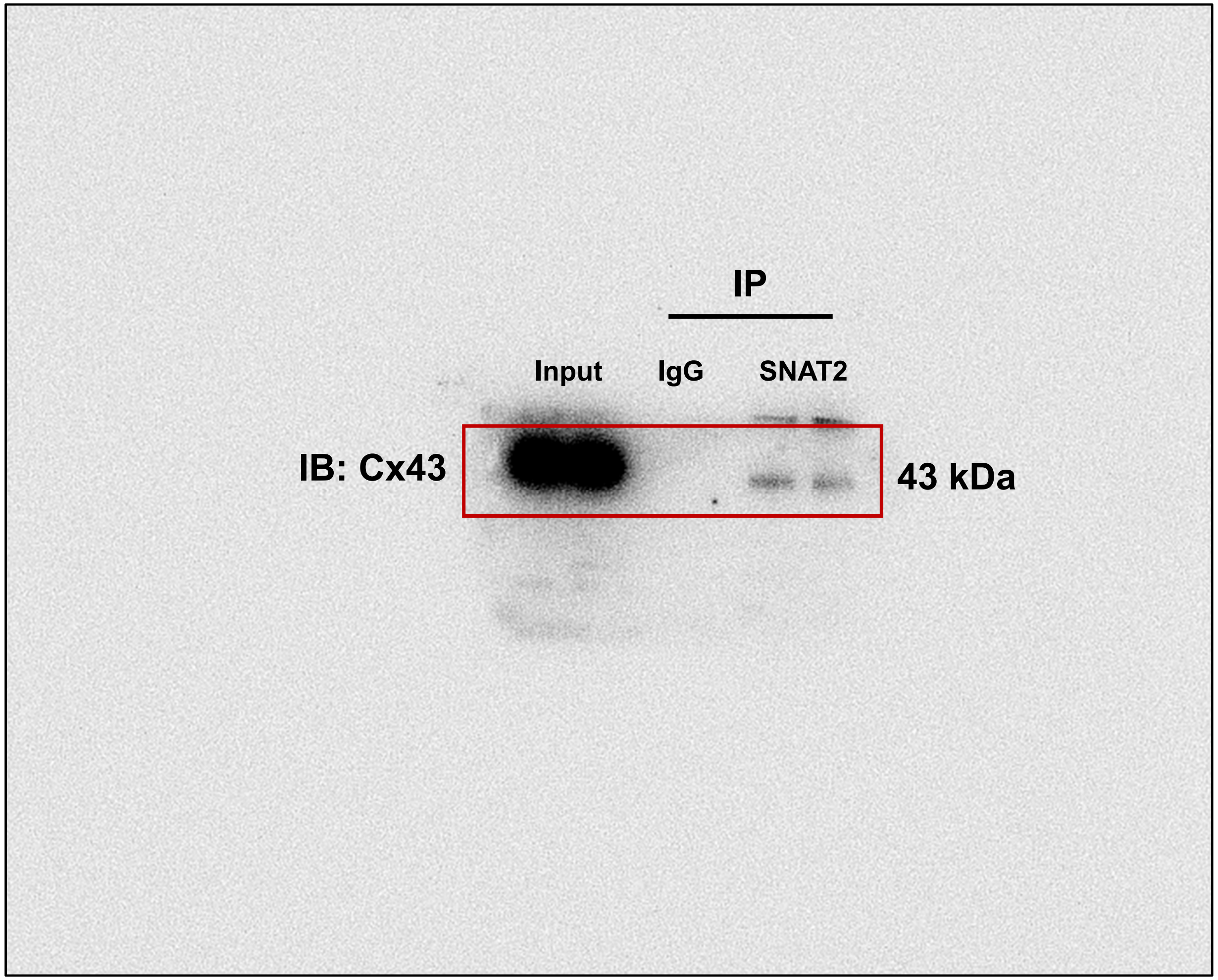

Full length blots of the Cx43 expression on the cell membrane of the 2-month-old WT mouse ventricular tissues. IgG served as the negative control. Red box indicates the cropped blots shown in Supplemental Figure 11D.

Cytoplasm

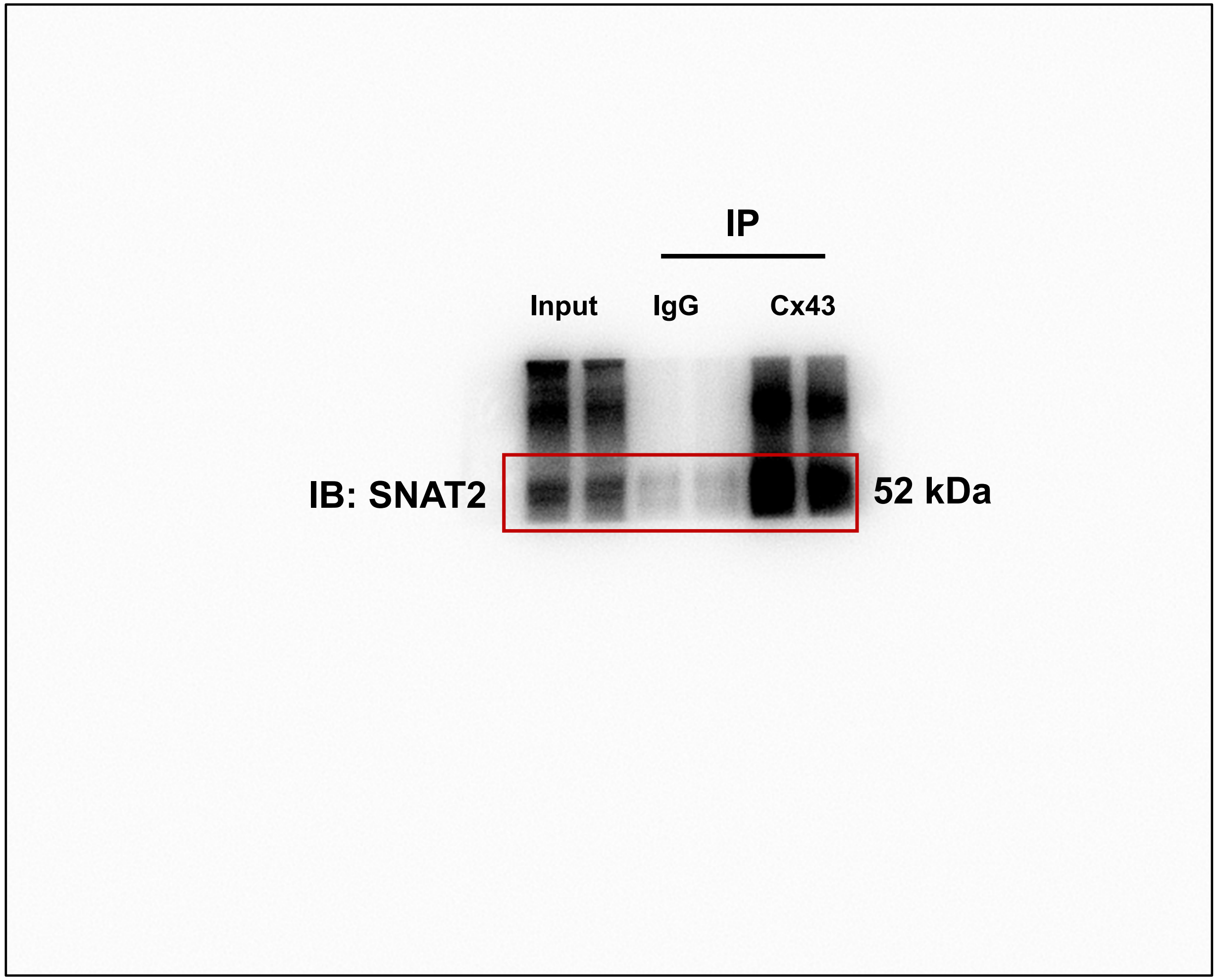

Full length blots of the SNAT2 expression in the cytoplasm of the 2-month-old WT mouse ventricular tissues. IgG served as the negative control. Red box indicates the cropped blots shown in Supplemental Figure 11D.

Cytoplasm

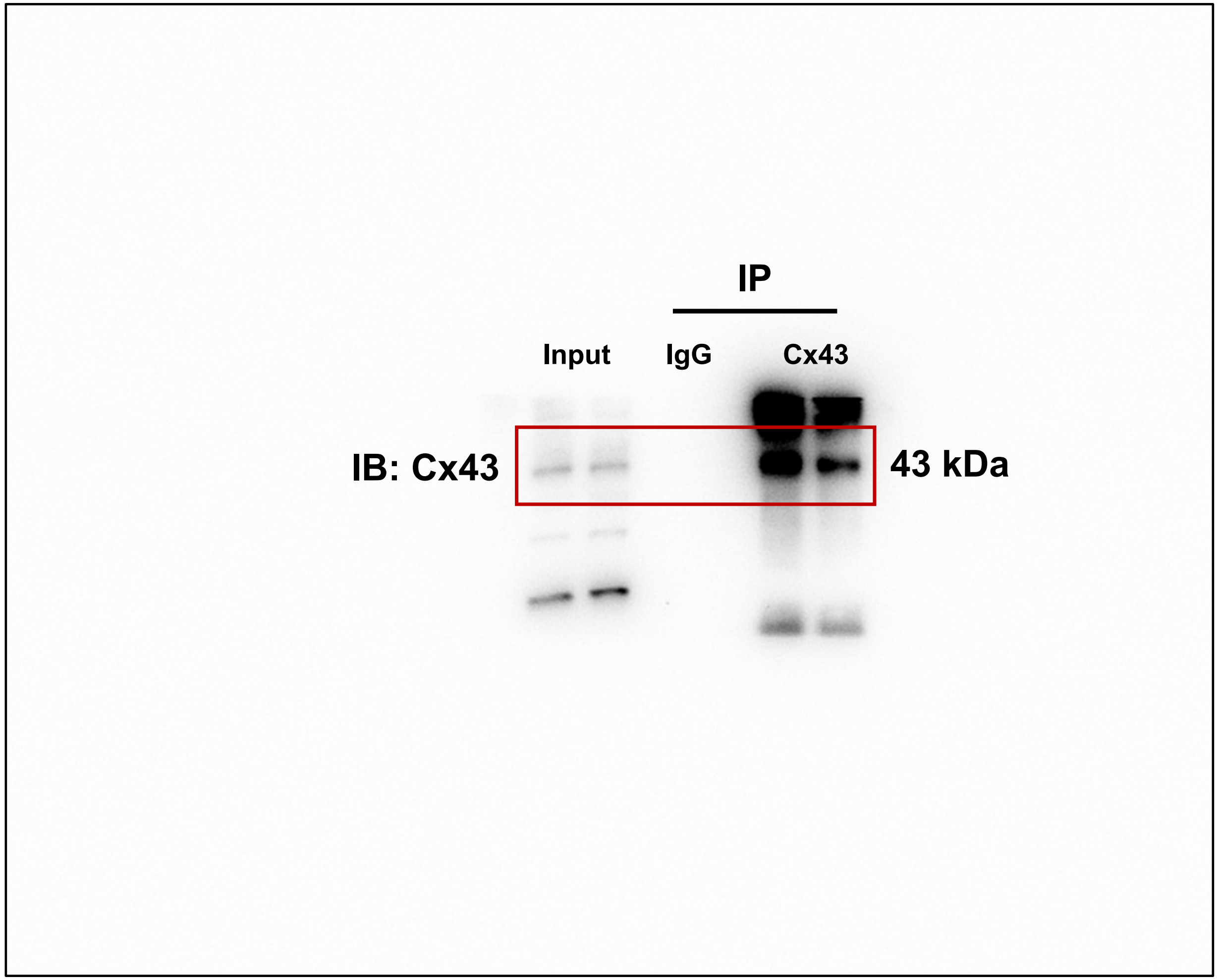

Full length blots of the Cx43 expression in the cytoplasm of the 2-month-old WT mouse ventricular tissues. IgG served as the negative control. Red box indicates the cropped blots shown in Supplemental Figure 11D.

Cytoplasm

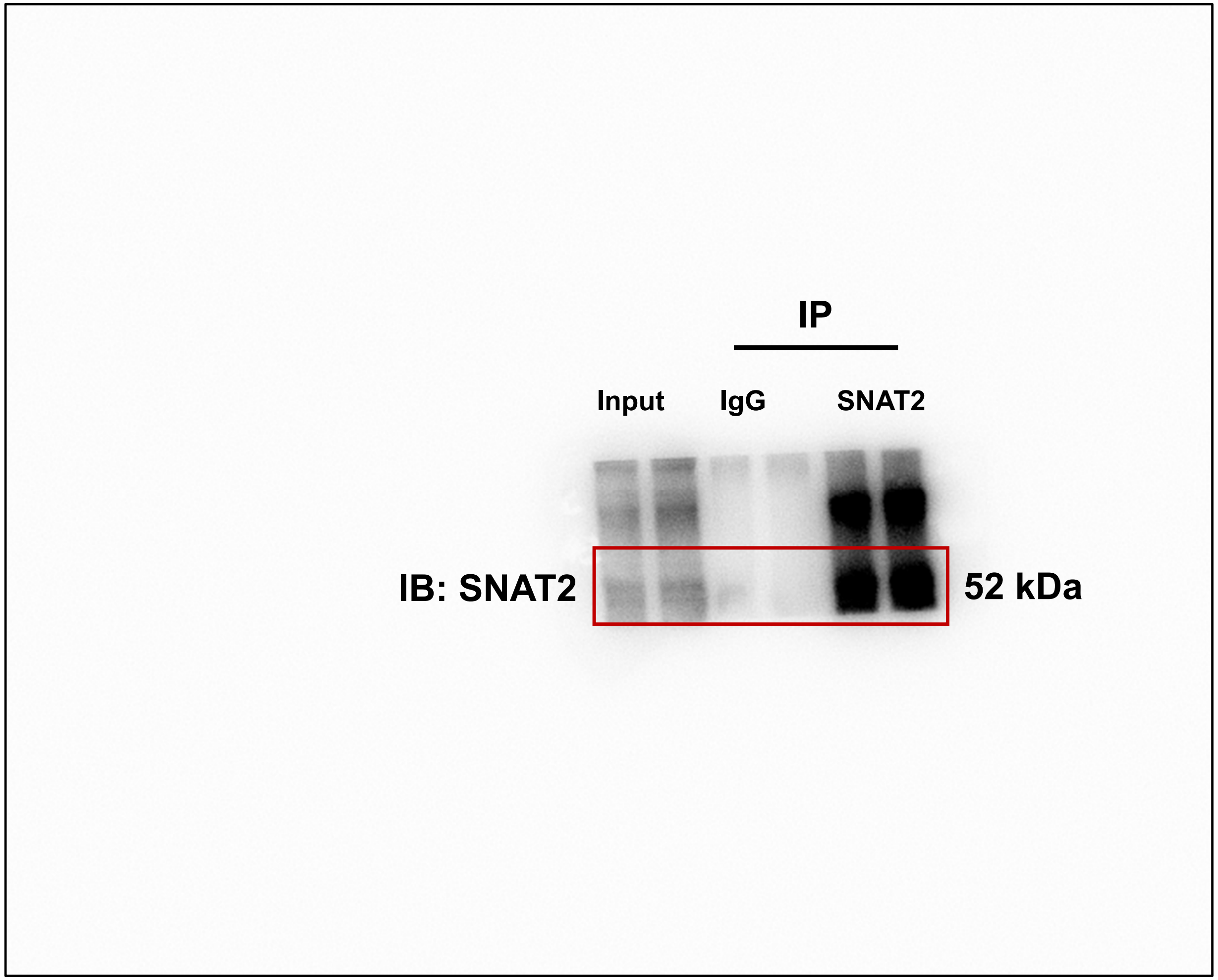

Full length blots of the SNAT2 expression in the cytoplasm of the 2-month-old WT mouse ventricular tissues. IgG served as the negative control. Red box indicates the cropped blots shown in Supplemental Figure 11D.

Cytoplasm

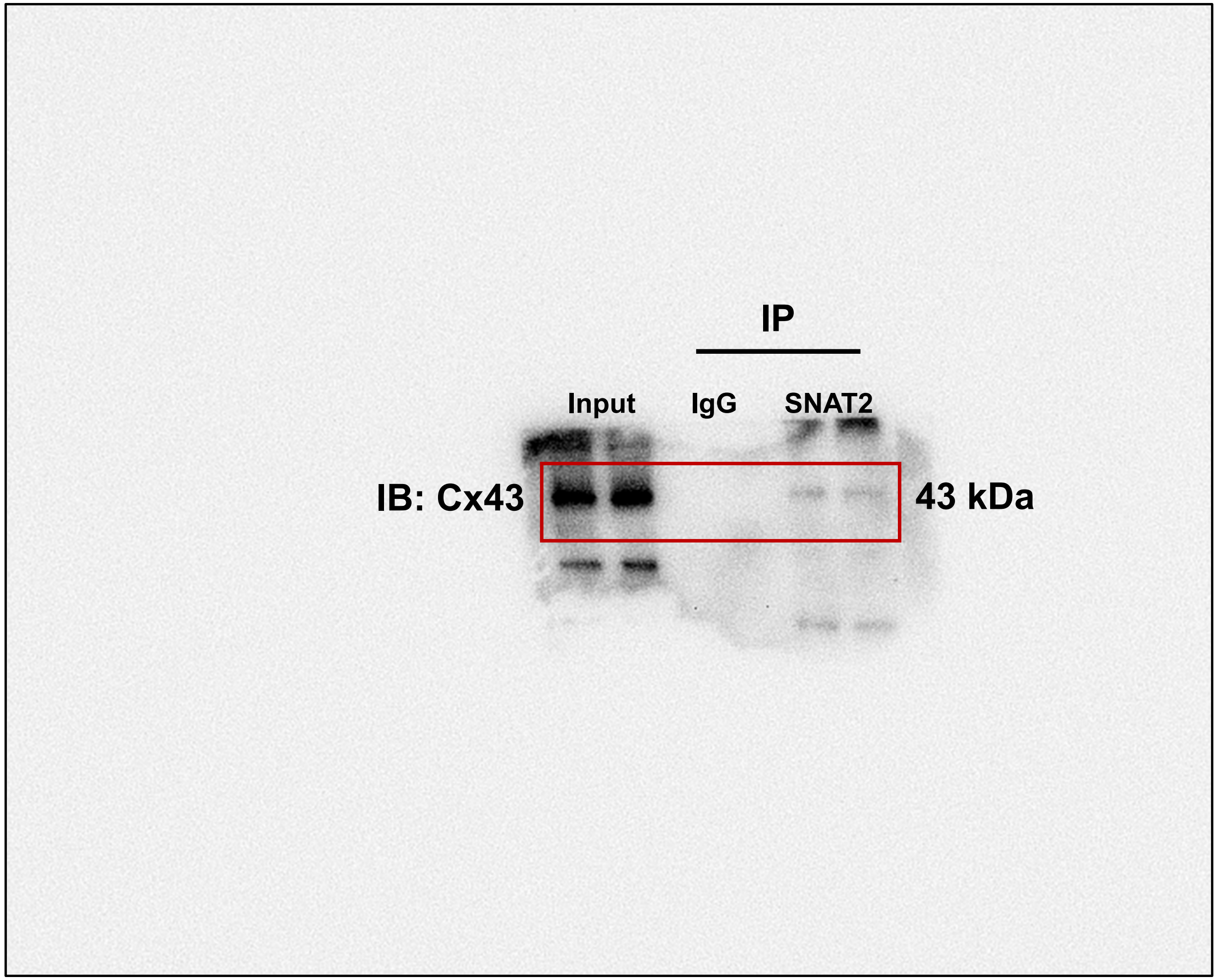

Full length blots of the Cx43 expression in the cytoplasm of the 2-month-old WT mouse ventricular tissues. IgG served as the negative control. Red box indicates the cropped blots shown in Supplemental Figure 11D.

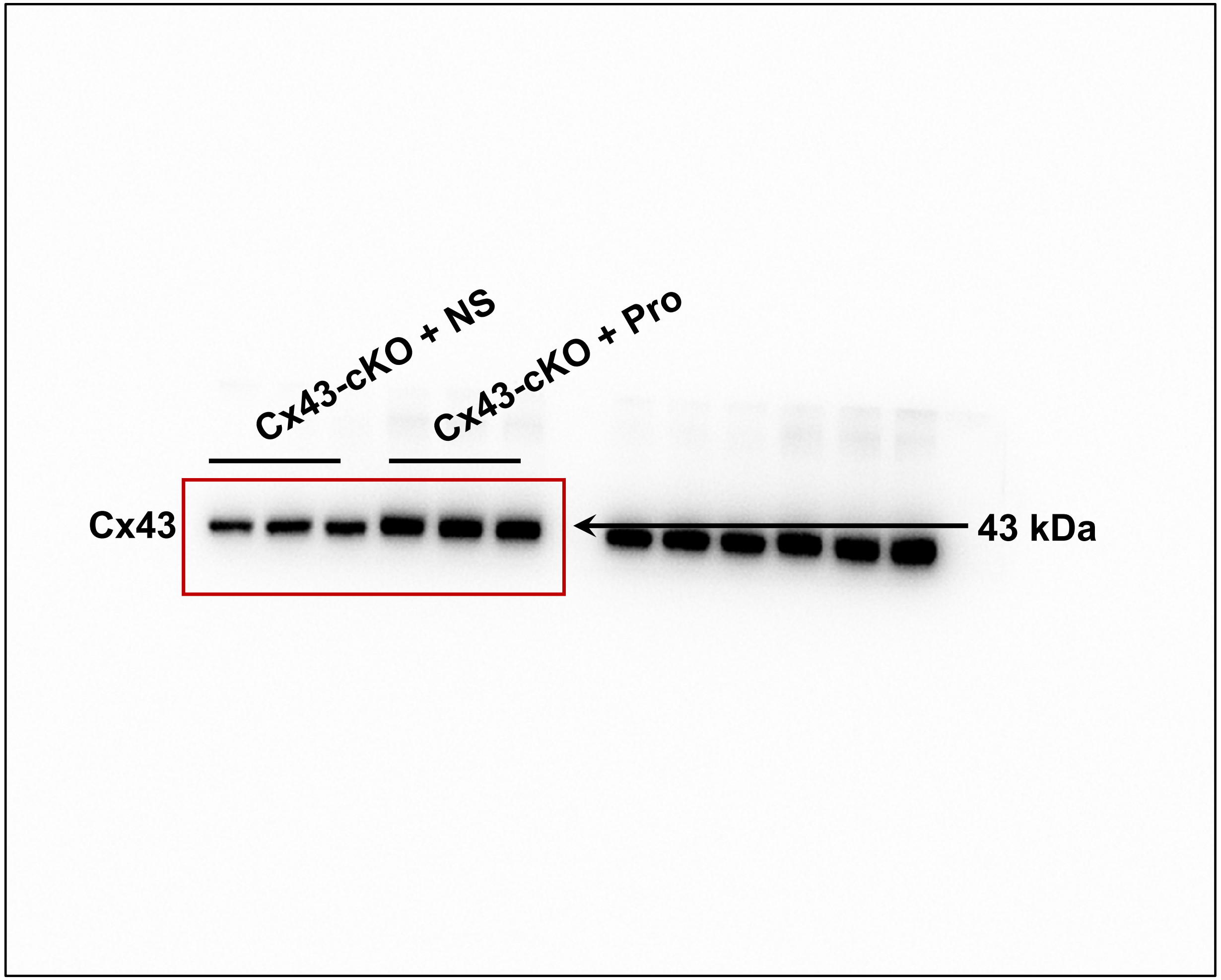

Full length blots of the Cx43 expression in ventricular tissues collected from 2-month-old HET Cx43-cKO mice fed on normal or proline-supplemented diet. Red box indicates the cropped blots shown in Supplemental Figure 12B.

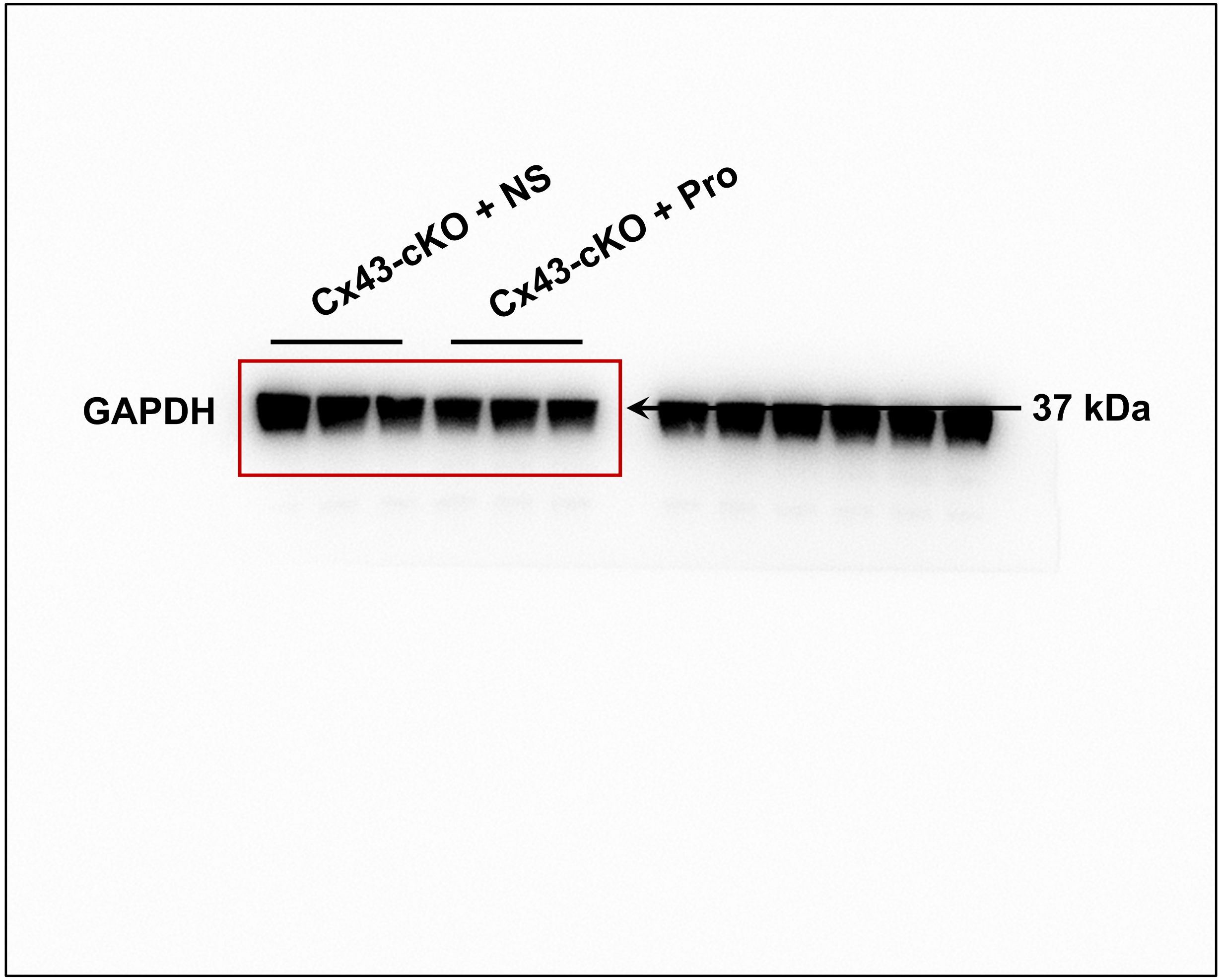

Full length blots of the GAPDH expression in ventricular tissues collected from 2-month-old HET Cx43-cKO mice fed on normal or proline-supplemented diet. Red box indicates the cropped blots shown in Supplemental Figure 12B.
